# Cardiometabolic trade-offs of carbohydrate- and fat-focused diets: a network meta-analysis of randomised clinical trials

**DOI:** 10.1007/s00394-026-04028-1

**Published:** 2026-07-08

**Authors:** Katerina Nikitara, Meropi D. Kontogianni, Anna-Bettina Haidich, Vasiliki Bountziouka

**Affiliations:** 1 Computer Simulations, Genomics and Data Analysis Lab, Department of Food Science and Nutrition, School of the Environment, University of the Aegean, Ierou Lochou 10 & Makrygianni, 81400 Lemnos, Greece; 2 Department of Nutrition & Dietetics, School of Health Sciences and Education, Harokopio University of Athens, 70 El. Venizelou St., 17676 Athens, Greece; 3 Laboratory of Hygiene, Social & Preventive Medicine and Medical Statistics, School of Medicine, Aristotle University of Thessaloniki, 54124 Thessaloniki, Greece; 4 Population, Policy and Practice Department, GOS Institute of Child Health, UCL, 30 Guildford Str., WC1N 1EH London, UK; 5 Dept. of Cardiovascular Sciences and Dept. of Population Health Sciences, School of Healthcare, University of Leicester, Cardiovascular Research Centre, Glenfield Hospital, LE3 9QP Leicester, UK

**Keywords:** Macronutrient composition, Dietary patterns, Cardiovascular risk factors, Lipid markers, Adiposity, Network meta-analysis

## Abstract

**Purpose:**

This study compared the cardiometabolic effects of carbohydrate- and fat-focused dietary patterns in adults at increased cardiovascular disease (CVD) risk, with emphasis on trade-offs across lipid profiles, adiposity, blood pressure, and glycaemic outcomes.

**Methods:**

A systematic literature search (January 2013-October 2025) identified randomised clinical trials enrolling adults aged 35–75 years with at least one CVD risk factor and evaluating dietary interventions with explicit macronutrient targets. Diets were classified based on fat and carbohydrate distribution, with monounsaturated fat content considered a marker of dietary quality and the low-fat—moderate-carbohydrate diet serving as the reference category. A random-effects frequentist network meta-analysis was conducted to estimate mean differences (with 95% confidence intervals) in CVD markers between dietary patterns.

**Results:**

Forty-seven studies (n = 3,450) were included. High-fat—low-carbohydrate diets were associated with modest reductions in body mass index compared with low-fat—moderate-carbohydrate diets [− 0.48 kg/m (− 0.74; − 0.22)]. Ketogenic diets showed the largest increases in LDL cholesterol [0.43 mmol/L (0.13; 0.74)] total cholesterol [0.55 mmol/L (0.13; 0.96)] and HDL cholesterol [0.12 mmol/L (0.06; 0.18)], compared with the reference diet, whereas triglyceride responses differed, with high-carbohydrate diets increasing them [0.24 mmol/L (0.04; 0.43)]. Macronutrient composition had limited effects on waist circumference, blood pressure and glycaemic markers.

**Conclusion:**

Findings demonstrate distinct cardiometabolic trade-offs between carbohydrate- and fat-focused dietary patterns, whereby carbohydrate restriction improves triglycerides, HDL cholesterol, and adiposity but is not consistently favourable for LDL cholesterol. These findings should be interpreted within the context of broad macronutrient targets rather than macronutrient quality and food-based dietary patterns. No single macronutrient distribution is universally optimal and dietary interventions should consider baseline metabolic profiles and macronutrient quality and quantity.

**Supplementary Information:**

The online version contains supplementary material available at 10.1007/s00394-026-04028-1.

## Introduction

Cardiovascular diseases (CVDs) remain the leading cause of mortality globally, with a substantial public health and socioeconomic burden [1]. The critical role of nutrition is well established, as diet-related factors account for approximately 36.7% of all CVD deaths, underscoring its importance for CVD prevention [2]. Earlier research was primarily directed towards single nutrients or individual foods [3]. However, the synergistic activity among macronutrients has necessitated a more complex analysis to capture the combined and interactive effects of diets [4].

Despite extensive research, evidence on the relationship between macronutrient composition and CVD risk markers remains heterogeneous, particularly for carbohydrate and fat intake, with conflicting findings across epidemiological and interventional studies [5–8]. This uncertainty is reflected in ongoing debates surrounding dietary recommendations. Specifically, while the World Health Organization advocates lowering total fat intake in favour of greater carbohydrate consumption [9], the recent guidelines from the U.S. Department of Health and Human Services identify fat and protein-containing foods as central components of a healthy dietary pattern [10]. In this context, network meta-analysis (NMA) provides a comparative framework for addressing inconsistencies by simultaneously evaluating the multidimensional effects of differing macronutrient distributions on cardiovascular risk outcomes.

In recent years, a growing number of NMAs have been published to investigate the multidimensional aspects of dietary interventions and their effects on CVD risk. Most of these NMAs have focused on broad dietary patterns (e.g., Mediterranean, DASH) [11–14] or on specific food categories [15, 16], whereas relatively few have examined the role of macronutrient composition and quality [17]. Moreover, many analyses rely on comparisons with usual or control diets that are poorly defined and heterogeneous across studies, limiting the interpretability of their findings [11–14]. Existing evidence also predominantly comprises trials conducted before 2013, reflecting a different landscape in nutritional science, and often includes populations with established CVD [11–14], raising concerns about the generalisability of results to contemporary populations and primary prevention settings. In addition, methodological challenges remain substantial, with inadequate transparency and inconsistent quality assessment undermining confidence in the validity of findings [18]. These issues are further compounded by the lack of standardised definitions for dietary interventions, leading many studies to adopt inductive approaches to intervention classification. Such approaches can result in overlapping and overly broad nodes that group heterogeneous dietary interventions, thereby obscuring differences between diets and potentially influencing effect estimates and intervention rankings [19].

Given these challenges, the aim of this NMA was to compare the effects of dietary interventions differing in macronutrient composition and quality on cardiovascular risk factors among adults at increased risk of CVD but without established disease. To improve interpretability and minimise overlap between dietary categories, intervention nodes were defined a priori using a prespecified framework based on clinically relevant macronutrient distribution targets. The resulting comparisons therefore reflect broad macronutrient-based dietary strategies rather than food-based dietary patterns, and do not fully capture qualitative dietary characteristics such as food sources, fibre content, glycaemic index, added sugars, or food matrix effects.

## Methods

In brief, randomised controlled trials (RCTs) enrolling adults aged 35–75 years with at least one cardiometabolic risk factor were eligible. Interventions involved dietary modifications with explicit quantitative targets in macronutrient composition and/or quality. Trials including participants with established cardiovascular disease, diabetes, major chronic conditions, or pregnancy were excluded. Primary outcomes included changes in adiposity (body mass index (BMI), waist circumference (WC)), lipid profile (low-density lipoprotein (LDL), high-density lipoprotein (HDL), total cholesterol (TC), triglycerides (TG)), systolic (SBP) and diastolic (DBP) blood pressure, and glycaemic markers (fasting glucose (FG), fasting insulin (FI)). Studies published in English from 2013 onwards were eligible. Five electronic databases were searched from January 2013 to October 2025. Study selection and data extraction followed prespecified criteria (Supplementary Table 1). The systematic review and NMA were conducted and reported in accordance with the PRISMA-NMA [20] (Supplementary Table 8) and prospectively registered in the PROSPERO (CRD42023427216) [21]. Detailed methodology is provided in the Supplement.

Diets were classified according to fat and carbohydrate distribution, with monounsaturated fat selected as the single quality marker because it was the most prevalent quality characteristic actively targeted as a primary intervention aim across the included studies. Fibre content and sodium reduction were also explored as additional quality-based classification dimensions but were not retained as the resulting network became too sparse to support meaningful comparative estimation. Full details of the node-making process are provided in the Supplement. Accordingly, the dietary interventions included in this NMA were categorised as follows, with macronutrient intake expressed as a percentage of daily energy intake (DEI):

1. High-MUFA diet (hMUFA): ≥ 20% of DEI from MUFA, irrespective of total fat/carbohydrate/protein distribution
2. Ketogenic (very-high-fat, very-low-carbohydrate): fat > 70%, carbohydrate ≤ 10%
3. High-fat—low-carbohydrate (HFLC): fat 36–70%, carbohydrate 11–44%
4. High-fat—moderate-carbohydrate (HFMC): fat 36–70%, carbohydrate 45–60%
5. Moderate-fat—low-carbohydrate (MFLC): fat 31–35%, carbohydrate 11–44%
6. Moderate-fat—moderate-carbohydrate (MFMC): fat 31–35%, carbohydrate 45–60%
7. Low-fat—moderate-carbohydrate (LFMC): fat ≤ 30%, carbohydrate 45–60%
8. Low-fat—high-carbohydrate (LFHC): fat ≤ 30%, carbohydrate > 60%

The LFMC diet was used as the reference comparator to maximise network connectivity.

### Statistical analysis

Network meta-analyses were performed in R (v4.5.1) [22] using a frequentist random-effects framework, with between-study variance estimated using the DerSimonian-Laird method employed within the netmeta package [23]. Relative treatment effects were estimated as mean differences (MDs) with 95% confidence intervals (CIs) under a consistency model. Heterogeneity and inconsistency were assessed using standard approaches. Intervention ranking was summarised using P-scores. Additional analyses, including meta-regression and assessment of small-study effects, are described in the Supplement.

Risk of bias was assessed using the Cochrane Risk of Bias 2 (RoB 2) tool [24], and the Confidence in Network Meta-Analysis (CINeMA) framework [25] was used to estimate the certainty of evidence across outcomes. Certainty ratings reflect the totality of network evidence; comparisons informed solely by indirect evidence were assessed using the same six-domain framework, with incoherence evaluated via the global design-by-treatment interaction test.

## Results

### Descriptive characteristics

Following screening, a total of 47 publications were included, reporting data from 40 unique RCTs (Supplementary Fig. 1), comprising 31 parallel-group trials (35 publications) [26–60] and 9 crossover trials (12 publications) [61–72], with a total sample size of 3,450 participants. Mean participant age ranged from 39.2 to 62.4 years, and mean BMI ranged from 27.0 to 49.0 kg/m^2^. Smoking status was reported in 37 publications [26–29, 31–39, 41–43, 46–50, 52, 53, 55, 56, 58–60, 62, 64–71], and medication use was reported in 44 publications [26–40, 42–53, 55–60, 62–72].

Recruitment periods spanned from 2005 to 2022, and all studies were conducted in community or outpatient settings. Most trials were conducted in North America and Europe, with additional studies from Asia and multinational cohorts. Intervention duration varied widely, ranging from 2 to 156 weeks. A run-in period, lasting approximately 1–10 weeks, during which participants followed habitual or standardised diets, was reported in twenty-two publications [30, 31, 34, 35, 37–40, 43, 50–52, 55, 56, 62, 64–67, 69–71]. Among crossover trials, washout periods were incorporated in most studies [62–71] and ranged from approximately 10 days to 10 weeks. Energy restriction was prescribed in at least one intervention arm and reported in nineteen publications [27, 28, 33, 41–43, 45, 49, 50, 52, 54, 56–61, 65, 66], whereas the remaining studies reported isocaloric diets across comparison groups. Controlled-feeding designs were reported in twenty publications [8, 30–32, 35, 37–39, 42, 43, 45, 47, 62–64, 67–71], in which all or most foods were provided, while self-prepared meals were reported in the remaining publications. Dietary interventions with structured moderate-intensity exercise programmes were reported in two publications [41, 60]. Dietary adherence was commonly monitored using food records or diaries, checklists and counselling. Additional study characteristics are summarised in Table 1.

**Table 1:** Descriptive characteristics of included studies (n = 47)

F. Author, Year	Study Design	Country	Interv. duration	Wash-out period	Preparation period	Interv. compliance method	Medication	Smoking	Excersise	Completion Rate	Age_Mean (SD)	BMI (kg/m2) (SD)	Sex_ Female	Energy-restrictive	Isocaloric_Between Groups
Andersson et al., 2016	Parallel-arm, blind	Sweden	24 months	N/A	Not reported	4-day estimated self-reported food records collected at baseline, monthly for 6 months, and at 9, 12, 18, and 24 months. Nitrogen excretion in urine (NU) was used as a biomarker for protein intake	No	No	Usual	70%	60 (1.3)	33 (0.9)	100%	No	Yes
Azadbakht et al., 2013	Parallel-arm	Iran	3 months	N/A	Not reported	3-day food record once a month, follow up once a month, Maroni formula along with urinary urea nitrogen (UUN)	Yes—dietary supplements, weight loss agents	No	Usual	100%	42 (2.8)	27 (1.15)	100%	Yes	Yes
Bajerska et al., 2018	Parallel-arm, double-blind	Poland	16 weeks	N/A	Not reported	Daily compliance questionnaire, follow up every 4 weeks	No	No	Usual	90%	60.5 (4.7)	33.7 (1.03)	100%	Yes	Yes
Duś-Żuchowska et al., 2018	Parallel-arm, double-blind	Poland	16 weeks	N/A	Not reported	Daily compliance questionnaire, follow up every 4 weeks	No	No	Usual	90%	60.5 (4.7)	33.6 (5.2)	100%	Yes	Yes
Bazzano et al., 2014	Parallel-arm	USA	12 months	N/A	Not reported	24-h dietary recalls at 0, 3, 6, and 12 months, weekly individual counselling sessions for the first 4 weeks, followed by small group counselling sessions every other week for the next 5 months (a total of 10 sessions) and monthly for the last 6 months of the intervention period	Yes—antihypertensive, lipid-lowering	Yes	Usual	80%	46.8 (10.1)	35.4 (4.2)	88%	No	Yes
Bergia et al., 2022	Parallel-arm	Multi-centred: Italy, Sweden, USA	12 weeks	N/A	3-week baseline period, when all subjects consumed their habitual, self-chosen, unrestricted diets	Daily diet record at weeks 4, 8, and 12 and 3 months after the intervention	Yes—antihypertensive, lipid-lowering	Yes	Usual	75%	55 (11)	30.7 (3)	56%	No	Yes
Boers et al., 2014	Parallel-arm	Netherlands	2 weeks	N/A	Not reported	Subjects were requested to keep records of the food consumed	Yes—antihypertensive, lipid-lowering	No	Usual	#REF!	53 (10)	32 (5.57)	73%	No	Yes
Chiu et al. 2017	Parallel-arm	California	3 weeks	N/A	3 week baseline diet	Food records on a weekly basis	No	No	Usual	98%	43 (13)	29 (2.72)	17%	No	Yes
Buscemi et al., 2013	Parallel-arm	Italy	12 weeks	N/A	Not reported	3-day food diary every 2 weeks and weekly visits	Yes—antihypertensive	Yes	Usual	85%	50 (7.96)	34.4 (5.83)	52.50%	Yes	Yes
Guo et al., 2022	Parallel-arm	China	10 weeks	N/A	Not reported	Snacks were provided to increase adherence to carbohydrates and proteins	Undefined	Yes	Moderate intensity	85%	39.2 (1)	29.5 (1.86)	72.50%	Yes	Yes
Tricò et al., 2021	Parallel-arm	Italy	4 weeks	N/A	Not reported	All participants underwent two sessions of behavioral dietary counseling	Undefined	Undefined	Usual	88,90%	44 (10.66)	49 (7.18)	68%	Yes	Yes
Veum et al., 2017	Parallel-arm	Norway	12 weeks	N/A	8 week run in period with vitamin and mineral supplementation	5-day food records each month	No	Undefined	Usual	82.6%%	40.3 (5.2)	33.7 (3.03)	0%	Yes	Yes
Belanger et al., 2023	Parallel-arm	Multi-centered	12 weeks	N/A	2-week run in period	Proided meals + daily food diary	Yes—lipid lowering	Undefined	Usual	92,50%	48 (10.02)	29.5 (4.85)	56.5%	No	Yes
Anderson-Vasquez et al., 2015	Crossover	Venezuela	28 days	No washout period	Not reported	24-h recall at home visits and 3 phone calls were made weekly	No	Undefined	Usual	100%	56 (5)	29.8 (3.1)	100%	No	Yes
Brassard et al., 2017	Crossover	Canada	4 weeks	≥ 24 days	4 week pre-intervention period	Checklists that were filled out by participants on a weekly basis	No	No	Usual	62%	39.2 (13.5)	30.8 (6)	53%	No	Yes
Chiu et al. 2014	Parallel-arm	USA	4 weeks	N/A	4 week baseline run-in diet	A 5-point compliance score was assigned by the dietitian using weekly interviews, menu checklists, and grocery receipts	No	No	Usual	91%	38 (12)	33.9 (3.8)	70.3%	No	Yes
Dorans et al., 2022	Parallel-arm	USA	6 months	N/A	Not reported	24-h dietary recalls from participants at baseline, 3 months, and 6 months & ketones in spot urine collected at baseline and follow-up visits	No	Yes	Usual	95%	58.9 (7.9)	35.9 (6.7)	72%	No	Yes
Ebbeling et al., 2021	Parallel-arm	USA	20 weeks	N/A	9–10 week run-in phase during which energy intake was restricted to promote 12 ± 2% weight loss	Meals provided by researchers	No	No	Usual	89.6%	35.7 (24–51.2)	32.2 (4.8)	69.4%	No	Yes
Gadgil et al., 2013	Crossover	USA	6 weeks	2–4 weeks	6 day run-in feeding period	Meals provided by researchers	No	Yes	Usual	98%	53.6 (10.9)	30.2 (6.1)	45%	No	Yes
Giacco et al., 2014	Parallel-arm	Italy	12 weeks	N/A	4 week run-in period	Meals provided by researchers	No	Undefined	Usual	84%	57 (8.3)	31.7 (5.59)	57.4%	No	Yes
Jackson et al., 2014	Parallel-arm	USA	12 weeks	N/A	Not reported	Meals provided by researchers	No	No	Usual	83%	46.1 (5.95)	33.2 (3.76)	50%	Yes	Yes
Kim et al., 2017	Parallel-arm	USA	4 weeks	N/A	Not reported	Meals provided by researchers + dietary record + point-and-shoot digital camera to record meal consumption	Yes—antihypertensive, lipid-lowering	Undefined	Usual	100%	62.4 (3.6)	37.5 (2)	58%	No	Yes
Jenkins et al., 2014	Parallel-arm	Canada	7 months	N/A	Not reported	7-day diet and exercise histories were recorded in the week prior to each monthly visit	Yes—antihypertensive	No	Usual	46%	56.5 (8.2)	37.1 (1.25)	61.5%	Yes	Yes
Juraschek et al., 2013	Crossover	USA	6 weeks	2–4 weeks	6 day run-in feeding period	Meals provided by researchers	No	18%	Usual	98%	53.6 (10.9)	30.2 (6.1)	45%	No	Yes
Krishnan et al., 2018	Parallel-arm	USA	8 weeks	N/A	Not reported	Daily meal checklist	No	No	Usual	85%	46.9 (12.6)	32.1 (3.9)	100%	No	Yes
Schroeder et al., 2015	Crossover	USA	4 weeks	10 days	Not reported	Daily questionnaires	No	No	Usual	87%	55.3 (8.7)	31.3 (4.4)	62%	No	Yes
Kirwan et al., 2016	Crossover	USA	8 weeks	10 weeks	Not reported	Weekly weigh-backs of food containers and calculated as the percentage of difference between prescribed and actual caloric intake	Yes—antihypertensive	No	Usual	83%	40 (7)	32.9 (4.5)	82%	No	Yes
Kitabchi et al., 2013	Parallel-arm	USA	6 months	N/A	Not reported	Weekly food diary	No	Undefined	Usual	75%	35.7 (2.05)	39.2 (2.7)	100%	Yes	Yes
Krishnan et al., 2012	Parallel-arm	USA	8 weeks	N/A	Not reported	Daily meal checklist	No	No	Usual	85%	46.9 (12.6)	32.1 (3.9)	100%	No	Yes
Uusitupa et al., 2013	Parallel-arm	Multi-centered: Finland, Sweden, Denmark, Iceland	18 weeks	N/A	4 week run-in period	4-day food record at weeks 2, 11, 17 or 23	Yes—antihypertensive, lipid-lowering	Yes	Usual	77,90%	55 (8.7)	31.6 (3.18)	67%	No	Yes
Mousavi et al., 2023	Parallel-arm	Iran	3 months	N/A	Not reported	6 dietary food records throughout the intervention period (once every 2 weeks)	No	No	Usual	88,60%	40 (8.1)	32 (3.61)	100%	Yes	Yes
Poulsen et al., 2014	Parallel-arm	Denmark	26 weeks	N/A	1 week run-in period	3 day food records at weeks 12 and 26	Yes—antihypertensive, lipid-lowering	Undefined	Usual	81%	42 (20–66)	30.2 (4.87)	71%	No	Yes
Raben et al., 2020	Parallel-arm	Multi-centered: Denmark, Finland, Netherlands, UK, Spain, Bulgaria, Australia, New Zealand	156 weeks	N/A	Not reported	4-day dietary records at 26, 52, 104 and 156 weeks	No	Yes	Moderate intensity	74%	51.4 (11.5)	35.3 (6.6)	68%	Yes	Yes
Rajaie et al., 2014	Crossover	Iran	6 weeks	2 weeks	2 week run-in period	3 day food records and 2 day physical activity record once every 2 weeks	Yes—antihypertensive, lipid-lowering	No	Usual	76,90%	42.4 (6.4)	33 (5)	100%	Yes	Yes
Rajaie et al., 2013	Crossover	Iran	6 weeks	2 weeks	2 week run-in period	3 day food records and 2 day physical activity record once every 2 weeks	Yes—antihypertensive, lipid-lowering	No	Usual	76,90%	42.4 (6.4)	33 (5)	100%	Yes	Yes
Sacks et al., 2014	Crossover	USA	5 weeks	2 weeks	8 day run-in phase	Daily food diary	No	Yes	Usual	86,20%	53 (11)	32 (6)	52%	No	Yes
Fechner et al., 2020 (a)	Parallel-arm	Maastricht, London	6 weeks	N/A	2 week run-in period	Participants were asked to monitor their daily food intake and to fill in a detailed 3-day food dairy during the run-in period and in the final week of the intervention	No	No	Usual	88,90%	60.8 (6.3)	29.3 (2.63)	52.5%	No	Yes
Fechner et al., 2020 (b)	Parallel-arm	Maastricht, London	6 weeks	N/A	2 week run-in period	Participants were asked to monitor their daily food intake and to fill in a detailed 3-day food dairy during the run-in period and in the final week of the intervention	No	No	Usual	88,90%	60.8 (6.3)	29.3 (2.63)	52.5%	No	Yes
von Frankenberg et al., 2015	Crossover	USA	4 weeks	10 days	10 day control diet	Daily food checklist	No	No	Usual	100%	36 (2.9)	33.6 (1.3)	30%	No	Yes
Marina et al., 2014	Crossover	USA	4 weeks	10 days	10 day control diet	Daily food checklist	No	No	Usual	100%	36 (2.9)	33.6 (1.3)	30%	No	Yes
Waliłko et al., 2021	Crossover	Poland	4 weeks	No washout period	Not reported	Food diaries	Undefined	Undefined	Usual	70%	41.5 (11)	33.6 (4.2)	88.6%	Yes	Yes
Pinsawas et al., 2024	Parallel-arm	Thailand	12 weeks (the intervention lasted 52 weeks, but after the 12th week compliance with targeted macronutrients was very poor	N/A	4 week run-in period	3-d food records monthly	Yes	No	Usual	84%	40.9 (7.9)	31.5 (0.87)	72%	Yes	Yes
Stomby et al., 2014	Parallel-arm	Sweden	24 months	N/A	Not reported	4-day estimated self-reported food records conducted at baseline, monthly for 6 months and at 9, 12, 18 and 24 months. Nitrogen excretion in urine (NU) was used as a biomarker for protein intake	No	No	Usual	70%	60 (1.3)	33 (3.4)	100%	No	Yes
Mellberg et al., 2014	Parallel-arm	Sweden	24 months	N/A	Not reported	4-day estimated self-reported food records conducted at baseline, monthly for 6 months and at 9, 12, 18 and 24 months. Nitrogen excretion in urine (NU) was used as a biomarker for protein intake	No	No	Usual	70%	60 (1.3)	33 (3.4)	100%	No	Yes
Hill et al., 2015	Parallel-arm	USA	12 weeks	N/A	2 week controlled feeding	Daily questionnaires	Yes	No	Usual	89,4%	45 (8.2)	35 (3.9)	54.8%	Yes	Yes
Ruth et al., 2014	Parallel-arm	USA	12 weeks	N/A	1 week	3-day food records (two weekdays and one weekend day) every two weeks	No	No	Usual	60,0%	42.5 (12.1)	36.5 (4.7)	88.50%	Yes	Yes
Wycherley et al., 2014	Parallel-arm	Australia	52 weeks	N/A	Not reported	Daily semiquantitative food records	Yes	Undefined	Usual	76	49.2 (1.1)	33.5 (0.5)	65%	Yes	No

### Network geometry

Network geometry was visualised using network plots to assess connectivity for all outcomes (Supplementary Figs. 2–5). Across outcomes, the networks were generally well connected, with LFMC and MFMC diets having the most direct comparisons. Networks for lipid outcomes were the most extensive and densely connected, whereas networks for adiposity and blood pressure outcomes included fewer nodes and comparisons, with some dietary interventions represented by only one or two studies. Despite this variability, all outcome-specific networks remained connected, allowing comparative estimation across dietary patterns. Detailed counts for all outcomes are presented in Supplementary Table 2.

### Heterogeneity

Overall, the degree of heterogeneity varied across outcomes, with lipid markers generally exhibiting greater heterogeneity than adiposity, blood pressure, and glycaemic measures. Adiposity outcomes showed no evidence of between-study or within-design heterogeneity (BMI & WC I^2^: 0%), although confidence intervals around I^2^ estimates were wide, suggesting limited precision. In contrast, substantial heterogeneity was observed for several lipid outcomes, particularly LDL, TC, and TG (I^2^ approximately 61.8–78.8%), whereas HDL cholesterol showed little evidence of heterogeneity (I^2^ = 0%). Blood pressure outcomes demonstrated low to moderate heterogeneity, with SBP showing borderline within-design heterogeneity (I^2^ = 40.3%), while DBP remained largely homogeneous (I^2^ = 17.7%). Glycaemic outcomes were characterised by low to moderate heterogeneity for FG (I^2^ = 36.9%) and no detectable heterogeneity for FI (I^2^ = 0%) (Supplementary Table 2). Given the substantial heterogeneity observed for several outcomes, particularly lipid markers, treatment rankings based on P-scores should be interpreted as indicative of relative positioning within the network rather than definitive evidence of superiority.

### Inconsistency

Consistency between direct and indirect evidence was assessed using global and local methods. Overall, there was no evidence of global inconsistency across outcomes (Supplementary Table 2), with only borderline evidence of global inconsistency for LDL. Heat plots (Supplementary Fig. 6a-j) indicated limited inconsistency, primarily arising from comparisons within lipid outcome networks involving carbohydrate-restricted dietary patterns. Node-splitting analyses (Supplementary Fig. 7a-j) were largely consistent with the heat-plot findings, showing no significant differences between direct and indirect evidence for adiposity, blood pressure, and glycaemic outcomes. Regarding lipid outcomes, selected comparisons involving carbohydrate-restricted dietary patterns and hMUFA diets versus LFMC and HFLC diets showed greater divergence between direct and indirect estimates. However, these discrepancies were isolated and did not indicate systematic inconsistency across network loops.

### Transitivity

Transitivity, a key assumption in NMA, requires that treatment comparisons are made across studies with comparable populations and study characteristics to allow valid indirect inference. To assess this assumption, we conducted a descriptive evaluation of key clinical and methodological characteristics across treatment comparisons, in line with PRISMA-NMA recommendations. Eligibility was restricted to studies enrolling adults aged 35–75 years who were free of established chronic diseases, focusing on individuals with cardiovascular risk factors to enhance clinical comparability across intervention nodes. Baseline characteristics, including age, sex distribution, cardiometabolic risk profile, medication use, intervention duration, concurrent behavioural or physical activity interventions, and energy restriction status, were examined across comparisons to identify potential imbalances (Supplementary Table 6 & Supplementary Fig. 9). Distributions of most clinical characteristics, including age, baseline BMI, total cholesterol, fasting glucose, and blood pressure, were broadly comparable across nodes. The most notable source of variability was intervention duration, which differed considerably within and across some nodes.

### Effects of diets with different macronutrient compositions on CVD risk factors

An overview of the results is presented in Supplementary Table 7, while the main findings for each outcome are described in detail below.

#### Adiposity outcomes

**Fig. 1:**
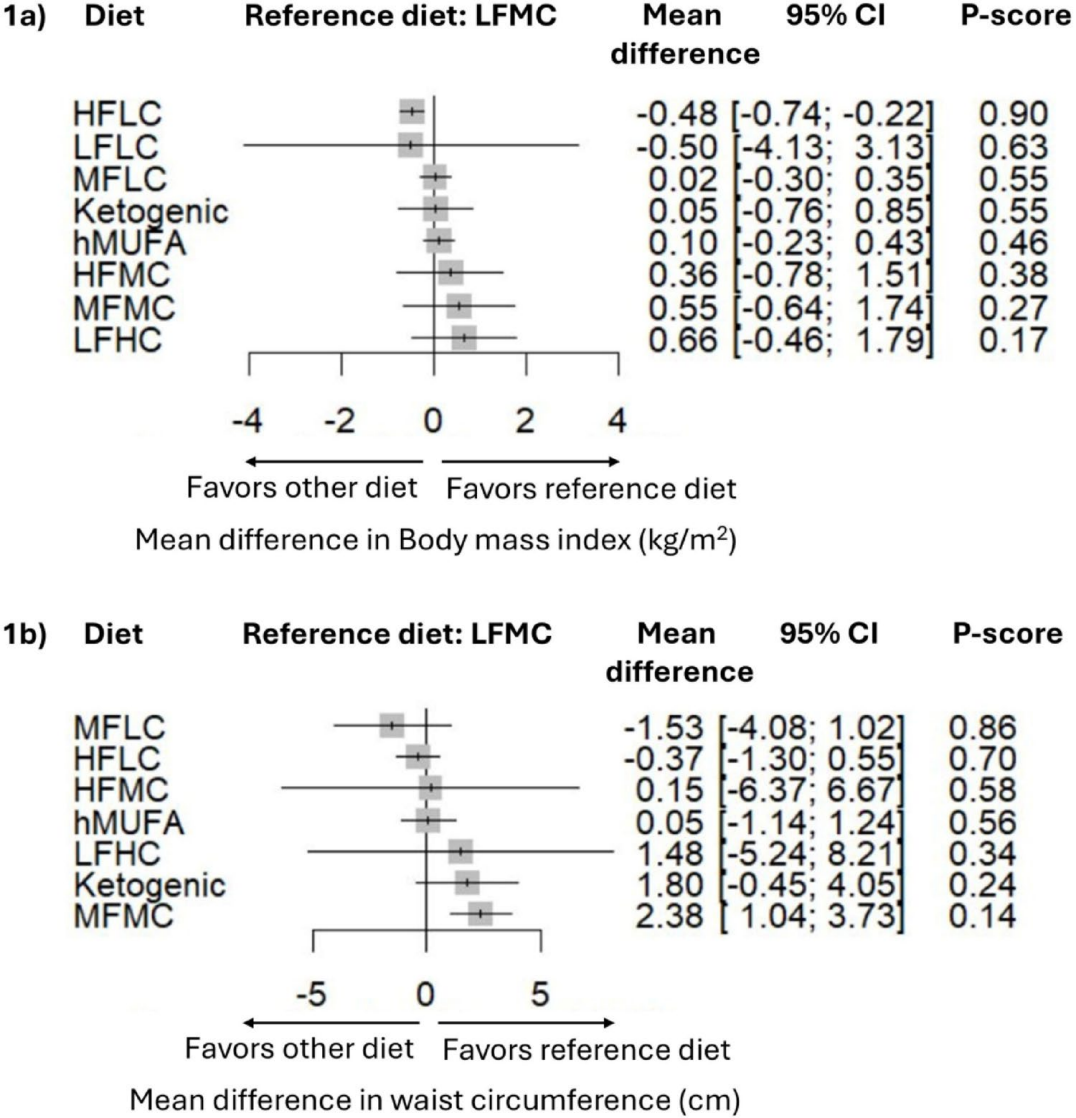
Forest plot of network meta-analysis results for a) body mass index, and b) waist circumference

The BMI network comprised 14 RCTs with a total sample of 1,260 participants [26, 32, 34, 41, 45, 47, 49, 52, 54, 56, 58, 66, 71, 72]. Compared with the reference diet (LFMC), HFLC was the most favourable ranking among the evaluated intervention and associated with a greater reduction in BMI (MD -0.48 kg/m^2^, 95% CI: -0.74 to -0.22 kg/m^2^; P-score 0.90; low certainty) (Fig. 1a). However, the wide confidence intervals indicate that the observed effect size remains uncertain and may reflect imprecision arising from limited direct comparisons and between-study variability.

The WC network included 14 RCTs with a total of 1,590 participants [28, 29, 32, 34, 46, 49–51, 54, 56, 58, 66, 71, 72]. MFMC ranked lowest among dietary interventions and was associated with an increase in WC compared with LFMC (MD 2.38 cm, 95% CI: 1.04 to 3.73 cm; P-score 0.14; high certainty) (Fig. 1b). In contrast, MFLC ranked highest, followed by HFLC and HFMC. However, these rankings were supported by low-certainty evidence and were characterised by wide confidence intervals. Therefore, the intervention hierarchy should be interpreted with caution.

#### Lipid outcomes

**Fig. 2:**
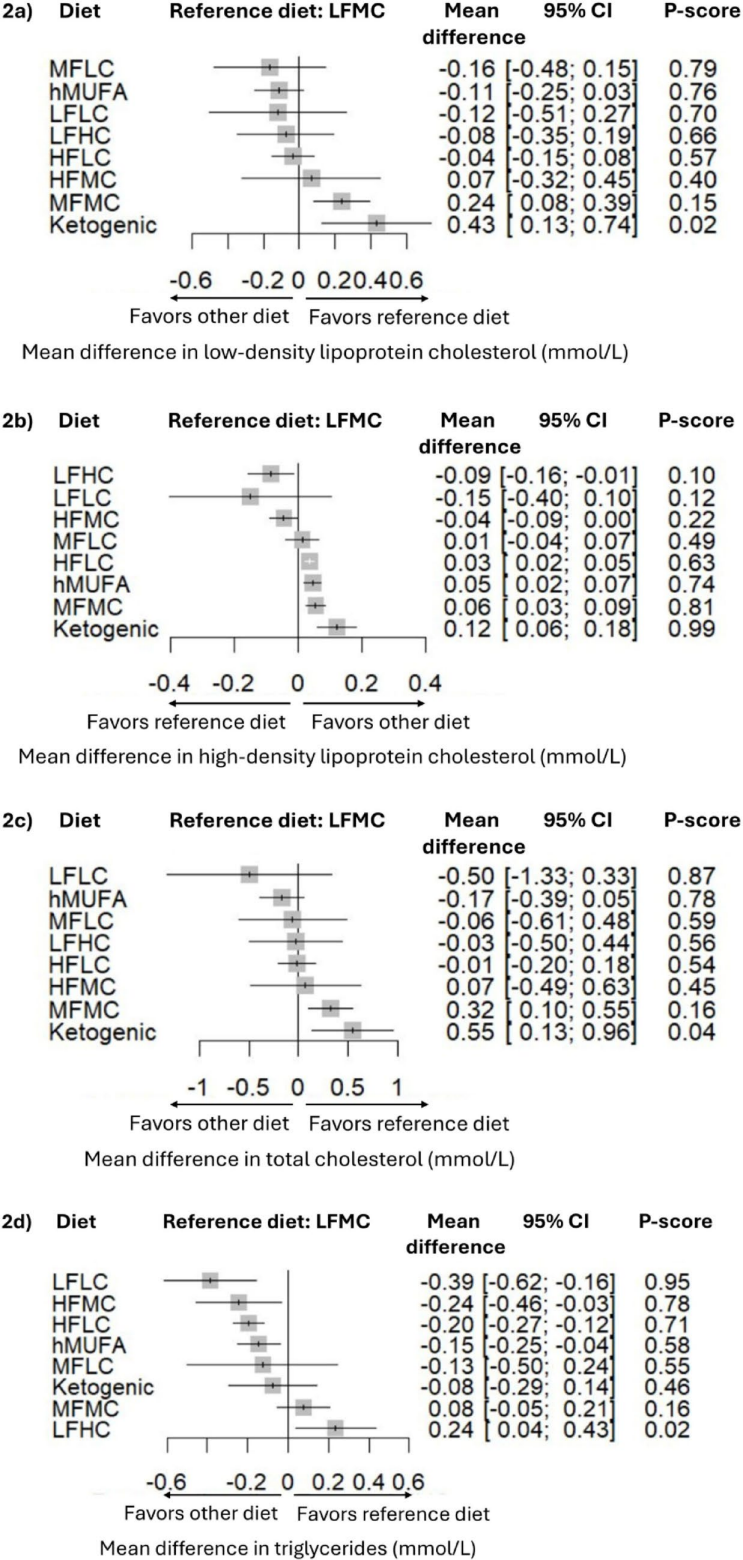
Forest plot of network meta-analysis results for a) low-density lipoprotein cholesterol, b) high-density lipoprotein cholesterol, c) total cholesterol, and d) triglycerides

The **LDL** network meta-analysis included 23 RCTs with a total of 2,579 participants [26, 28, 29, 32, 34, 35, 37, 38, 41, 46, 49–52, 56, 58, 61, 63, 64, 66, 67, 71, 72]. Compared with the reference diet (LFMC), ketogenic diets were associated with an increase in LDL (MD 0.44 mmol/L, 95% CI 0.13 to 0.74 mmol/L; P-score 0.02; moderate certainty) (Fig. 2a), followed by MFMC diets (0.24 mmol/L, 95% CI 0.08 to 0.40 mmol/L; P-score 0.15; moderate certainty). Confidence intervals for LDL estimates were relatively narrow, compared with other lipid outcomes, indicating greater precision. In terms of treatment hierarchy, ketogenic and MFMC diets ranked lowest for LDL outcomes, with moderate certainty of evidence.

The **HDL** network included data from 23 RCTs involving 2,579 participants [26, 28, 29, 32, 34, 35, 37, 38, 41, 46, 49–52, 56, 58, 61, 63, 64, 66, 67, 71, 72]. Compared with the LFMC reference diet, several dietary patterns were associated with increases in HDL, albeit with small effect sizes (Fig. 2b). Ketogenic diets showed the greatest increase in HDL (MD 0.12 mmol/L; 95% CI 0.06 to 0.18 mmol/L; P-score 0.99; high certainty), followed by MFMC (MD 0.06 mmol/L; 95% CI 0.03 to 0.09 mmol/L; P-score 0.81; high certainty) and hMUFA diets (MD 0.05 mmol/L; 95% CI 0.02 to 0.07 mmol/L; P-score 0.74; high certainty). HFLC diets were also associated with a modest increase in HDL (MD 0.03 mmol/L; 95% CI 0.02 to 0.05 mmol/L; P-score 0.63; high certainty). Conversely, LFHC diets were associated with a reduction in HDL (MD -0.09 mmol/L; 95% CI -0.16 to -0.01 mmol/L; P-score 0.10; high certainty). Regarding treatment hierarchy, ketogenic diets ranked highest for increasing HDL, followed by MFMC and hMUFA-based diets, whereas LFHC and LFLC ranked lowest.

A total of 22 RCTs with 2,007 participants [26, 28, 29, 32, 34, 35, 37, 38, 41, 46, 49–52, 56, 58, 61, 63, 66, 67, 71, 72] contributed to the **TC** network. Ketogenic diets were associated with an increase in TC compared with LFMC, with a small-to-moderate effect size (MD 0.55 mmol/L; 95% CI 0.13 to 0.96 mmol/L; P-score 0.04; moderate certainty). MFMC diets also increased TC, though the effect size was smaller (MD 0.32 mmol/L, 95% CI 0.10 to 0.55 mmol/L; P-score 0.16; moderate certainty) (Fig. 2c). Ranking probabilities were consistent with these estimates, with ketogenic and MFMC diets ranking lowest, but should be interpreted cautiously, due to wide confidence intervals and a moderate certainty of evidence.

The **TG** network included 24 RCTs with a total of 2,603 participants [26, 28, 29, 32, 34, 35, 37, 38, 41, 45, 46, 49–52, 56, 58, 61, 63, 64, 66, 67, 71, 72]. LFLC had the greatest reduction compared to LFMC (MD -0.39 mmol/L, 95% CI -0.62 to -0.16 mmol/L; P-score 0.95; high certainty), followed by HFMC (MD -0.24 mmol/L, 95% CI -0.46 to -0.03 mmol/L; P-score 0.78; moderate certainty), HFLC (MD -0.20 mmol/L, 95% CI -0.27 to -0.12; P-score 0.71; moderate certainty), and hMUFA diets (MD -0.15 mmol/L, 95% CI -0.25 to -0.04 mmol/L; P-score 0.58; moderate certainty) (Fig. 2d). In contrast, there is evidence of an association between LFHC diets and increased TG concentrations, compared to the reference diet (MD 0.24 mmol/L, 95% CI 0.04 to 0.43 mmol/L; P-score 0.02; moderate certainty). Low- and moderate-carbohydrate diets ranked highest for triglyceride reduction, whereas LFHC ranked lowest.

#### Blood pressure outcomes

**Fig. 3:**
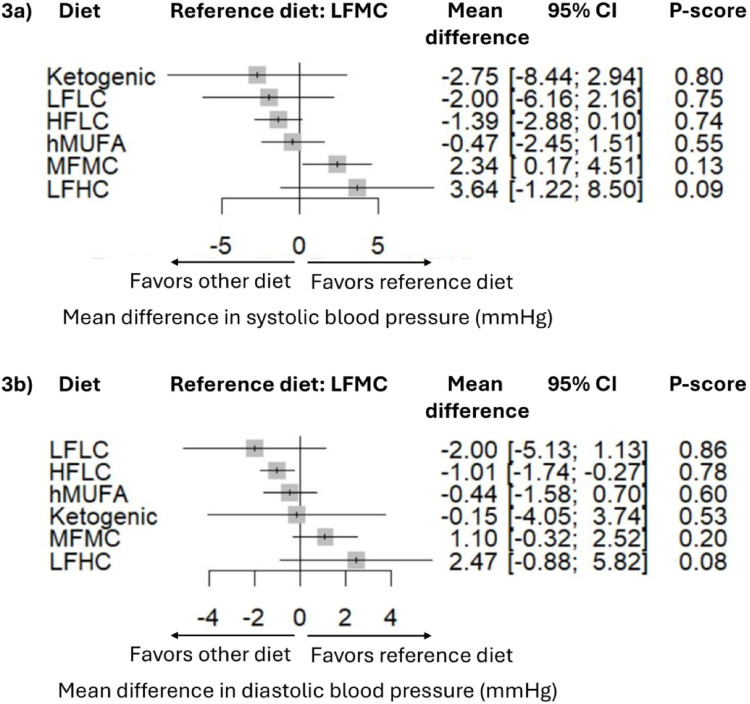
Forest plot of network meta-analysis results for a) systolic, and b) diastolic blood pressure

The networks for **SBP** and **DBP** each included 17 RCTs and a total of 1,326 participants [26, 28, 29, 32, 37, 38, 45, 46, 49–52, 58, 63, 64, 69, 71]. Overall, dietary macronutrient composition had limited effects on blood pressure outcomes. No dietary pattern was associated with a change in SBP levels compared with the reference diet, LFMC (Fig. 3a). Regarding DBP, the HFLC diet was associated with a modest reduction compared with LFMC (MD -1.01 mmHg, 95% CI -1.74 to -0.27 mmHg; P-score 0.78; low certainty) (Fig. 3b).

#### Glycaemic outcomes

**Fig. 4:**
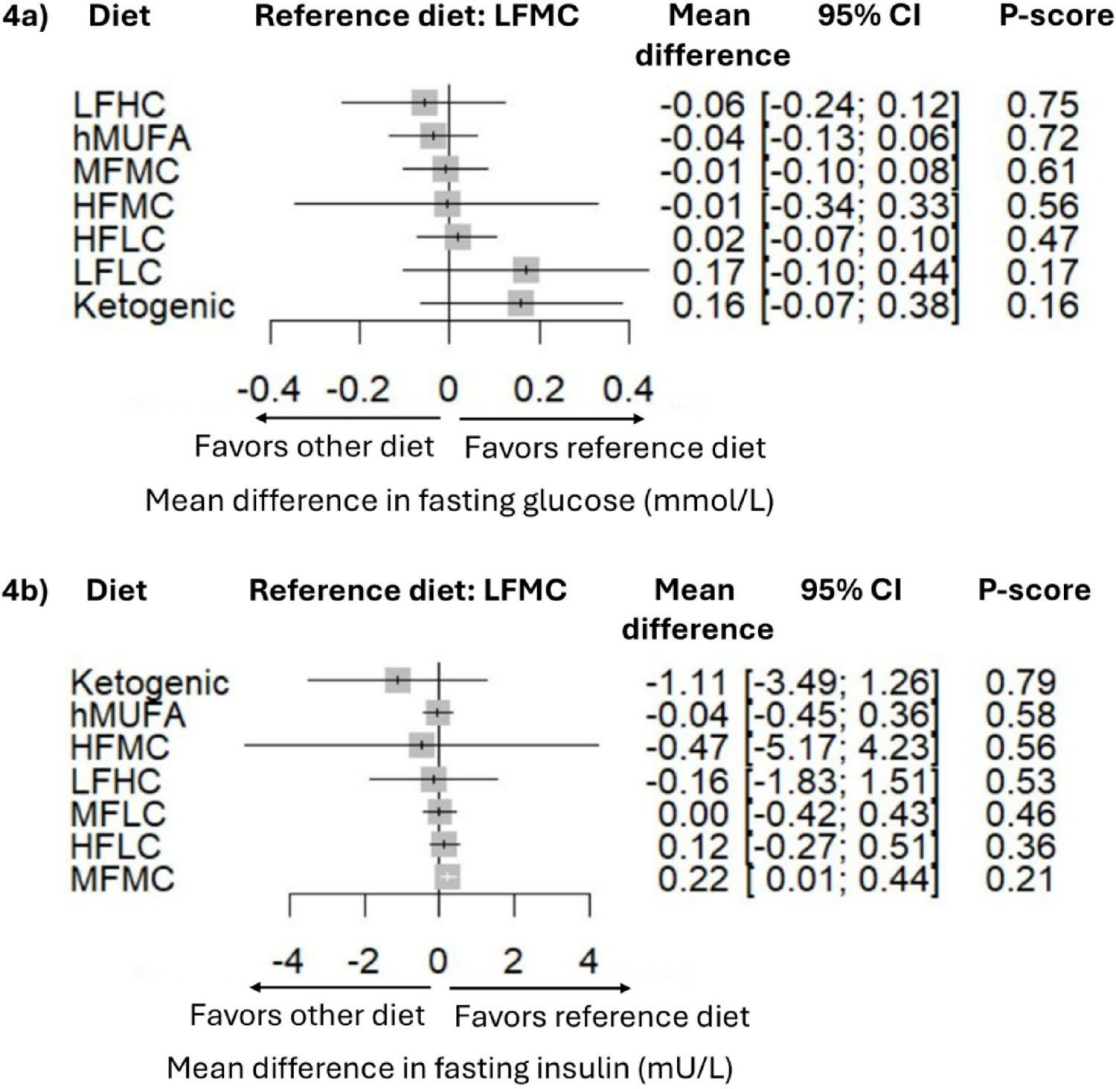
Forest plot of network meta-analysis results for a) fasting glucose, and b) fasting insulin

Across glycaemic outcomes, the **FG** network included 22 studies with a total of 2,088 participants [26, 28, 29, 32, 34, 38, 41, 46, 49–52, 54, 56, 58, 61–63, 66, 70–72], while the FI network included 19 studies with a total of 1,961 participants [26, 28, 29, 32, 34, 38, 41, 46, 49–52, 54, 56, 62, 63, 66, 70, 71]. Overall, dietary macronutrient composition showed limited and inconsistent effects on glycaemic outcomes, with no sufficient evidence of an effect across both FG and FI. Ketogenic diets were associated with a reduction in FI but an increase in FG, whereas hMUFA-based diets showed no meaningful change in either fasting insulin or fasting glucose (Fig. 4). The certainty of evidence ranged from low to moderate, warranting cautious interpretation of these findings.

### Meta-regression analysis

Meta-regression analyses examined whether intervention duration, sex composition (proportion of female participants), and energy restriction status modified the treatment effects across outcomes (Supplementary Table 5). No evidence of modification was observed for BMI, WC, HDL, SBP, or DBP.

Energy restriction modified lipid responses in LFHC diets, with energy-restricted LFHC interventions associated with higher LDL (β = 1.59 mmol/L, 95% CI 0.43 to 2.75) and total cholesterol concentrations (β = 1.66 mmol/L, 95% CI 0.04 to 3.28) compared with unrestricted LFHC diets. Sensitivity analyses stratifying trials by energy restriction status were broadly consistent with the primary analysis across most dietary nodes and outcomes (Supplementary Figs. 10–19). The most notable exception was ketogenic diets, where significant increases in LDL and total cholesterol were observed only among energy-restricted trials, with insufficient data available in the non-energy-restricted stratum to draw firm conclusions. This suggests that concurrent energy restriction may partly account for the lipid effects attributed to this node. By contrast, the BMI reduction associated with HFLC diets was consistent regardless of energy restriction status, indicating a macronutrient-driven rather than energy-deficit-driven effect. Blood pressure and glycaemic outcomes were unaffected by energy restriction in both strata, consistent with the primary analysis.

A higher proportion of female participants was associated with attenuated LDL-lowering effects in hMUFA diets (β = 1.19 mmol/L, 95% CI 0.42 to 1.96) and LFHC diets (β = 1.78 mmol/L, 95% CI 0.43 to 3.13), and with less favourable total cholesterol responses in both hMUFA diets (β = 1.75 mmol/L, 95% CI 0.36 to 3.14) and LFHC diets (β = 2.43 mmol/L, 95% CI 0.42 to 4.43). A statistically significant association between sex distribution and triglyceride concentrations was also observed for LFLC diets; however, this estimate (β = 7.02 mmol/L, 95% CI 0.55 to 13.5) was based on only two contributing studies and likely reflects statistical instability rather than a genuine biological effect.

Longer intervention duration was associated with a progressive attenuation of the LDL-lowering effect of LFHC diets (β = 0.15 mmol/L, 95% CI 0.0004 to 0.30), with a similar pattern observed for total cholesterol. Regarding glycaemic outcomes, longer duration was associated with higher fasting glucose concentrations in HFLC (β = 0.006 mmol/L, 95% CI 0.001 to 0.011) and MFMC diets (β = 0.014 mmol/L, 95% CI 0.006 to 0.022), but lower fasting insulin concentrations (β = -0.10 mU/L, 95% CI -0.21 to -0.003), suggesting divergent temporal effects on glucose and insulin regulation.

### Publication bias and small-study effect

Small-study effects and potential publication bias were assessed using comparison-adjusted funnel plots (Supplementary Fig. 8a-j) and Egger’s regression test for funnel plot asymmetry (Supplementary Table 2) across all outcomes. Overall, there was little evidence of funnel plot asymmetry, and most outcomes showed no indication of small-study effects. Funnel plots for adiposity (BMI, WC), blood pressure (SBP, DBP), and glycaemic (FG, FI) outcomes appeared largely symmetric, suggesting no substantial small-study effects. Lipid outcomes were similarly well balanced, although LDL cholesterol showed minor visual asymmetry. However, this pattern was not consistent across lipid markers and was not supported by statistically significant results from Egger’s regression test.

### Risk of bias and certainty of evidence

The risk of bias assessment was performed for both parallel and crossover RCTs. Among the parallel studies, most trials were judged to have a low overall risk of bias (Supplementary Table 3a). Only a small number of studies were rated as having a high risk of bias, mainly due to issues in handling missing outcome data, lack of allocation concealment, or deviations from the protocol. Regarding crossover trials, they demonstrated a low risk of bias, indicating acceptable internal validity. However, two studies were judged to be at high risk, largely due to concerns about period and carryover effects or outcome measurement (Supplementary Table 3b).

The CINeMA estimates varied across outcomes but were generally moderate to low, with only a few comparisons achieving high confidence (Supplementary Tables 4a-**j**). For BMI and WC, ratings were mostly low to moderate due to imprecision and within-study bias or heterogeneity. Confidence across lipid outcomes was more variable. The LDL estimates were mainly low to moderate due to heterogeneity and imprecision, whereas most HDL comparisons were rated with high confidence. TC and TG comparisons were typically moderate, with downgrading driven mainly by heterogeneity and incoherence, and high confidence was observed only for a few TG comparisons. Evidence for blood pressure outcomes was generally weaker, with SBP and DBP mostly rated as low confidence due to imprecision and heterogeneity. For glycaemic outcomes, FG comparisons were moderate to high in confidence, whereas FI estimates were predominantly low to moderate due to imprecision, heterogeneity, and occasional incoherence.

## Discussion

Diets differing in macronutrient composition exert distinct and sometimes opposing effects on cardiometabolic risk factors. In this network meta-analysis of RCTs, no single dietary strategy consistently optimised all outcomes; instead, carbohydrate- and fat-focused dietary patterns were associated with clinically relevant trade-offs in adiposity and lipid markers, while effects on blood pressure and glycaemic markers were limited. These findings should be interpreted within the context of broad macronutrient distribution targets rather than comprehensive food-based dietary patterns, as important aspects of diet quality, including food sources, fibre, glycaemic index, added sugars, and food matrix effects, were not fully captured by the classification framework. Regarding adiposity, high-fat-low-carbohydrate diets were associated with modest reductions in BMI and ranked most favourably among interventions, although the evidence was of low certainty. The observed effect, corresponding to approximately 1.3–1.6 kg in adults of average height, falls below the 5–10% weight-loss threshold typically associated with meaningful improvements in cardiovascular risk factors [73]. This suggests that, despite statistical significance, the clinical impact of this level of weight reduction may be limited. Our findings are consistent with those of recently published NMAs, although those studies reported larger absolute weight changes when diets were stratified more aggressively by carbohydrate and protein targets. For example, Lou et al. [17], who examined different combinations of carbohydrate and protein proportions, demonstrated a weight reduction of approximately -4.1 kg for very low-carbohydrate-low-protein diets and -1.3 to -1.5 kg for very low/moderate–carbohydrate-high-protein patterns. Likewise, a separate synthesis of RCTs reported greater reductions with ketogenic diets (approximately -10.5 kg) and high-protein diets (approximately -4.5 kg), along with large decreases in waist circumference for ketogenic (approximately -11 cm) and low-carbohydrate diets (approximately -5 cm) [12]. These discrepancies might reflect differences in the definitions of low-carbohydrate and ketogenic diets across studies, differences in populations, as the other NMAs included adults with chronic diseases, unlike our NMA, as well as differences in co-interventions and adherence, particularly the extent to which energy restriction, behavioural support, and intervention intensity accompanied macronutrient prescription.

Across lipid outcomes, divergent effects were observed according to macronutrient composition. Evidence of moderate certainty indicated an association between ketogenic and moderate-fat-moderate-carbohydrate diets and increases in LDL and TC, with these diets also ranking lowest in the treatment hierarchy. Interestingly, ketogenic diets produced the largest increases in HDL cholesterol, followed by moderate-fat-moderate-carbohydrate diets, high-monounsaturated fat (hMUFA) diets, and high-fat-low-carbohydrate diets, whereas low-carbohydrate diets reduced HDL concentrations. A similar pattern emerged for TG. Low- and moderate-carbohydrate diets consistently lowered TG levels, with low-fat-low-carbohydrate diets showing the greatest reduction, whereas low-fat—high-carbohydrate diets led to increases in TG. Additional NMAs further support this trade-off, showing that very low-carbohydrate diets were among the least effective at lowering LDL cholesterol despite favourable reductions in TG [12, 17]. Notably, several dietary patterns, including moderate carbohydrate and very-low-carbohydrate proportions, were reported to achieve TG decreases of approximately 0.14–0.33 mmol/L, magnitudes that are comparable to those observed in our network, reinforcing the metabolic sensitivity of triglycerides to carbohydrate intake [17]. Despite these areas of agreement, some discrepancies across reviews likely reflect heterogeneity in macronutrient definitions, protein targets, comparator diets, and baseline population characteristics. Importantly, consistent with our findings, prior syntheses generally report minimal differences between dietary strategies for total cholesterol, blood pressure, and glycaemic outcomes, suggesting that macronutrient composition alone may have stronger effects primarily within specific lipid markers rather than across the full cardiometabolic spectrum.

Our findings demonstrate that lipid changes across diets with different macronutrients require careful clinical contextualisation, given the varying strength of association between specific lipid fractions and cardiovascular outcomes. Evidence suggests that each 1.0 mmol/L reduction in LDL cholesterol is associated with approximately a 10% reduction in all-cause mortality and about a 20% decrease in major vascular events [74]. Accordingly, the observed increases in LDL in our study with ketogenic (0.44 mmol/L) and moderate-fat-moderate-carbohydrate diets (0.24 mmol/L) may be clinically relevant, particularly if sustained over time. Although carbohydrate-restricted diets improved HDL-C levels, with ketogenic diets producing the greatest increase (0.03–0.12 mmol/L), the clinical significance of these changes for CVD risk reduction is uncertain [75]. Recent evidence raises questions about whether raising HDL-C alone reduces CVD risk independent of broader metabolic changes, and pharmacological trials that selectively raised HDL-C did not demonstrate cardiovascular benefit [76]. In contrast, LDL-C remains one of the most robustly established modifiable risk factors for cardiovascular disease. The observed increases in LDL-C associated with ketogenic diets in this analysis are therefore of greater clinical concern than the accompanying HDL-C changes. These findings highlight the importance of careful and regular lipid monitoring, particularly LDL-C, when ketogenic or very-low-carbohydrate dietary approaches are implemented, especially in individuals with pre-existing dyslipidaemia or elevated cardiovascular risk. The results for hMUFA diets further illustrate the interaction between diet quality and macronutrient distribution. Improvements in HDL cholesterol and triglycerides were observed with hMUFA diets regardless of macronutrient composition, suggesting that MUFA enrichment may exert independent favourable effects on these lipid fractions. However, the LDL effect of hMUFA diets appeared to depend on the broader macronutrient context, reinforcing the principle that diet quality and macronutrient distribution interact in determining lipid outcomes and that neither dimension alone fully explains the cardiometabolic response. It should be noted that the lipid effects observed here reflect broad macronutrient distribution targets and are likely to be substantially modified by diet quality. In particular, the extent to which ketogenic and high-fat diets increase LDL cholesterol is likely influenced by the type of fat consumed, with diets emphasising saturated fat expected to produce more adverse lipid profiles than those emphasising unsaturated fat sources. These findings therefore cannot be generalised to specific food-based dietary patterns without accounting for fat quality, fibre content, and food matrix effects.

A similar interpretive challenge applies to TG. While reductions in TG levels have been associated with approximately an 11–14% decrease in CVD risk per 1 mmol/L reduction [77], the TG decreases identified here were considerably smaller (-0.15 to -0.39 mmol/L) and warrant interpretation alongside the simultaneous elevation in LDL with ketogenic patterns (0.44 mmol/L). From a clinical perspective, these patterns reinforce the importance of individualised lipid surveillance when adopting high-fat and carbohydrate-restricted diets.

Overall, high-fat—low-carbohydrate diets showed a modest improvement in BMI, HDL and TG, without increasing the other markers. However, that is not the case for very high-fat and very low-carbohydrate interventions, which may warrant additional caution, as they indicate a benefit in HDL while simultaneously having a negative impact on LDL and TC. Our findings suggest that the cardiovascular implications are likely shaped by the qualitative composition of dietary fats, as hMUFA diets improved both HDL and TG despite differences in other macronutrient composition, emphasising the need for a holistic risk assessment rather than reliance on macronutrient distribution alone. This is further supported by a recent perspective study demonstrating that both low-carbohydrate and low-fat diets can improve TG and HDL in their healthy versions [78]. The convergence of existing evidence also strengthens the clinical message that diets that improve TG and HDL may have neutral or unfavourable effects on LDL cholesterol. Because LDL is a key causal determinant of cardiovascular risk, improvements in other lipid fractions may not compensate for increases in LDL. Therefore, LDL should be assessed when dietary strategies are being considered.

In addition to the impact on cardiovascular risk markers, NMAs examining macronutrient composition and cardiovascular mortality further support the clinical relevance of lipid changes observed in the present study. Substitution analyses indicate that replacing 5% of energy from carbohydrates with unsaturated fats, particularly polyunsaturated fatty acids (PUFA), including both n-6 and n-3 fatty acids, as well as plant-derived MUFA is associated with meaningful reductions in mortality risk [79]. Similarly, replacing saturated and trans fats with PUFA or MUFA, substituting animal-derived fats with plant sources, and replacing animal protein with plant protein have all been linked to lower mortality, reinforcing the importance of fat quality rather than total fat quantity [80]. This body of evidence suggests that the cardiometabolic impact of diet is driven not only by macronutrient distribution but also by macronutrient quality, highlighting its importance for long-term cardiovascular risk reduction.

In contrast to the lipid outcomes, macronutrient composition did not significantly affect glycaemic markers or blood pressure in the present analysis, suggesting that these outcomes may depend more on dietary factors beyond macronutrient ratios. For glycaemic regulation, factors such as dietary fibre, whole-grain intake, food structure, and overall carbohydrate quality likely play a more decisive role than total carbohydrate quantity, given their established effects on glucose absorption, insulin sensitivity, and postprandial responses. This interpretation is consistent with broader nutritional evidence indicating that diets rich in fibre and minimally processed carbohydrates improve glycaemic control independent of macronutrient distribution. A similar explanation may apply to blood pressure outcomes, where the absence of meaningful differences between dietary strategies suggests that micronutrient composition, particularly sodium and potassium intake, may be more influential determinants of blood pressure. These findings further underscore that the macronutrient distribution framework used in this analysis does not capture the dietary dimensions most relevant to these outcomes, reinforcing the importance of diet quality and food-based characteristics beyond macronutrient ratios.

Finally, meta-regression analyses indicated that energy restriction, sex and intervention duration modified cardiometabolic responses to dietary interventions, highlighting that cardiometabolic responses to macronutrient manipulation are not uniform and reinforcing the importance of considering patient characteristics, intervention design, and follow-up duration when translating dietary evidence into clinical practice. A higher proportion of female participants in the included studies was associated with a weaker lipid-improving effect of the dietary interventions. This observation may partly reflect sex-related differences in habitual macronutrient intake, as women often report higher relative fat and sugar intake, whereas men typically report higher absolute macronutrient intakes but lower relative fat intake, potentially influencing metabolic responses to dietary interventions [80]. Physiological differences in lipid metabolism may also contribute, as clinical and experimental evidence indicates that sex hormones influence plasma lipid and lipoprotein profiles [81].

### Strengths and limitations

A major strength of this study is the use of a comprehensive NMA, which enabled simultaneous comparison of multiple dietary patterns differing in macronutrient composition across several cardiometabolic outcomes. An additional strength is the application of a structured dietary classification framework based on predefined thresholds for macronutrient quantity and quality, which minimised overlap between intervention groups and improved the interpretability of comparative estimates. Clearly defined inclusion criteria were also implemented to support the transitivity assumption, required for valid indirect comparisons.

Several limitations should be acknowledged. Reporting in some primary studies was insufficient to fully characterise macronutrient quality, limiting our ability to distinguish between diets with similar macronutrient distributions but differing food sources, and meaning that within-category heterogeneity in diet quality likely remains. Substantial heterogeneity also existed in the implementation of dietary interventions and contextual factors affecting adherence, and many trials were conducted under controlled feeding conditions with relatively short intervention durations, which may limit generalisability and long-term inference about the sustainability and cardiometabolic effects of these dietary strategies. Regarding the transitivity assumption underlying the network comparisons, although distributions of key clinical and methodological characteristics were broadly comparable across dietary intervention nodes, considerable variability in intervention duration, energy restriction status, and study design was observed across some nodes. Limited reporting of potential effect modifiers, such as medication use, further restricted exploration of sources of heterogeneity. In addition, the review process was conducted by a single reviewer, which represents an important limitation that increases the risk of selection bias or data extraction errors. Although all uncertainties were discussed with the principal investigator and the review followed prespecified eligibility criteria and extraction forms, these safeguards do not fully substitute for independent dual review and the possibility of systematic error cannot be excluded. Finally, the focus on macronutrient composition rather food-based dietary patterns means that interactions between food quality, dietary matrices, and metabolic health were not fully captured.

## Conclusion

This NMA indicates that no single macronutrient distribution consistently optimises cardiometabolic risk factors, as dietary patterns exert heterogeneous effects across adiposity, lipid, glycaemic, and blood pressure markers. Carbohydrate-restricted diets improved triglycerides and HDL cholesterol and produced modest reductions in BMI, but these effects were accompanied by neutral or adverse effects in LDL cholesterol. In contrast, macronutrient composition in terms of quantity had limited influence on glycaemic markers and blood pressure, suggesting that factors beyond macronutrient ratios, including dietary fibre, sodium intake, and food sources, may also play an important role. These findings highlight the importance of considering individual metabolic characteristics when interpreting dietary responses. Ketogenic diets may warrant careful clinical monitoring, particularly regarding lipid responses and dietary fat quality. Future research should further characterise macronutrient quality and food-based dietary patterns to better understand their role in cardiovascular risk.

## Supplementary Information

Below is the link to the electronic supplementary material.

Supplementary Material 1

## Data Availability

The data supporting the findings of this study are derived from publicly available studies cited in the manuscript. Extracted data and analytic code used in the network meta-analysis are available from the corresponding author upon reasonable request.
