# Supplementary material for: Cardiometabolic trade-offs of carbohydrate- and fat-focused diets: a network meta-analysis of randomised clinical trials
Source: Eur J Nutr. 2026 Jul 8;65(5):204. doi: 10.1007/s00394-026-04028-1 (PMC13346310; doi:10.1007/s00394-026-04028-1)
Supplement: Supplementary file 1 — Supplementary Material 1 [file 394_2026_4028_MOESM1_ESM.docx]

**On-line supplementary material**

**Cardiometabolic trade-offs of carbohydrate- and fat-focused Diets: A network meta-analysis of randomised clinical trials**

**Nikitara et al.**

**METHODS**

To investigate the impact of diets with different macronutrient compositions and qualities, we conducted a systematic review and network meta-analysis (NMA) of randomised controlled trials (RCTs) comparing diets with differing macronutrient compositions. The systematic review and network meta-analysis were conducted and reported in accordance with the extension of PRISMA (Preferred Reporting Items for Systematic Reviews and Meta-Analyses) guidelines, incorporating network meta-analyses (PRISMA-NMA) [1]. The study protocol was prospectively registered in the PROSPERO database (registration number: CRD42023427216) [2].

**Eligibility criteria**

Study eligibility was determined a priori using the PICOS framework (Population, Intervention, Comparator, Outcomes, Study design), as outlined below.

***Population***

Studies were considered eligible if they recruited adults aged 35-75 years with at least one cardiometabolic risk factor, including overweight or obesity, hypertension, dyslipidaemia (elevated total or LDL cholesterol or reduced HDL cholesterol), or prediabetes, as defined in each trial. We excluded trials that enrolled participants with established diabetes or other chronic conditions (gastrointestinal, respiratory, cardiovascular, metabolic, hepatic, or renal) and those that included pregnant or breastfeeding women.

***Interventions***

Eligible interventions involved dietary modifications characterised by explicit changes in macronutrient intake and/or macronutrient quality (e.g., monounsaturated fat content, glycaemic index, or dietary fibre), with quantitative reporting of intake levels. Diets described using broad food-based labels (e.g., Mediterranean, DASH, Nordic, ketogenic) were included only when macronutrient targets or achieved intake levels were clearly reported, allowing consistent classification across studies. Studies in which macronutrient changes occurred as secondary consequences of the dietary pattern were excluded. Interventions focusing solely on micronutrient supplementation, time-restricted eating or fasting regimens, single foods or food groups, or individual meals were excluded. Studies incorporating meal replacements, dietary supplements, or interventions combined with high-intensity exercise programmes were also excluded to minimise heterogeneity unrelated to dietary composition.

***Comparators***

Comparators were alternative dietary interventions that met the same eligibility criteria but differed from the intervention of interest in macronutrient composition and/or quality.

***Outcomes***

Primary outcomes were changes in adiposity, lipids, blood pressure, and glycaemic biomarkers, including body mass index (BMI), waist circumference (WC), low-density lipoprotein cholesterol (LDL-C), high-density lipoprotein cholesterol (HDL), total cholesterol (TC), triglycerides (TG), systolic blood pressure (SBP), diastolic blood pressure (DBP), fasting glucose (FG), and fasting insulin (FI).

***Study design***

Only randomised controlled trials (RCTs) were included, using either parallel-group or crossover designs.

***Timeframe and language restrictions***

Studies published from 2013 onwards were considered eligible. This restriction was applied to reflect the increasing emphasis in recent nutrition research on overall dietary patterns and macronutrient composition rather than isolated nutrients or single foods. Only studies published in English were included.

**Search strategy**

We systematically searched five electronic databases (PubMed, Scopus, Web of Science, CINAHL, and ProQuest) for peer-reviewed articles in two stages: an initial search run in May 2023 and an update conducted in October 2025 to capture recently published studies. Search strategies were tailored to each database and combined terms for (i) dietary interventions and macronutrient composition, (ii) the target population, (iii) lipid, blood pressure, glycaemic and adiposity outcomes, and (iv) RCTs. The search strings are provided in **Supplementary** **Table 1**. The search strategy was originally developed for a broader systematic review examining both RCTs and observational studies on dietary macronutrient composition and cardiometabolic outcomes. Accordingly, study design terms including “cohort”, “prospective”, and “case-control” retained in the search strings. For the purposes of the present network meta-analysis, eligibility was restricted exclusively to RCTs, and all non-randomised studies were excluded during screening. In addition, we screened grey literature, the reference lists of eligible studies and relevant prior reviews to identify additional potentially eligible trials. Minor discrepancies in hit counts upon replication may arise from retrospective MeSH term assignment and database indexing updates, which is expected and consistent with Cochrane guidance on search reproducibility.

**Study selection and data extraction**

Records were imported into EndNote for deduplication and screened in Rayyan. Titles and abstracts were screened against explicit eligibility criteria, followed by full-text assessment. Screening was performed by a single reviewer, and any potential issues were resolved with the Principal Investigator. Data were extracted using a prespecified form capturing study design, participant characteristics, intervention and comparator details (including macronutrient targets or achieved intake), and outcome data. For continuous outcomes, we extracted group-level means and measures of variability at baseline and at the end of the intervention, or change-from-baseline and/or between-group differences when reported. The assessment closest to the end of the intervention was used. Intention-to-treat estimates were prioritised, and per-protocol results were used when unavailable. Study identifiers were recorded to assess potential overlap of study populations. Where multiple publications reported data from the same trial, these were treated as a single study unit in the analysis, with data extracted complementarily across publications to avoid double-counting of participants.

**Definition of dietary intervention categories (node-making)**

The classification system distinguished diets by fat and carbohydrate intake, allowed protein to vary, and retained monounsaturated fat (MUFA) as a prespecified quality marker. Protein intake was not included as a primary classification dimension because variation in protein composition across interventions was limited.

The hMUFA node was intentionally defined using a diet-quality criterion rather than overall macronutrient distribution, and this was a prespecified decision grounded in the nature of the available evidence base. Several included RCTs were specifically designed to investigate MUFA enrichment as the primary dietary target, independently of total fat or carbohydrate intake. Many of these were Mediterranean or DASH-type interventions whose primary distinguishing dietary characteristic was MUFA enrichment, and assigning them to any of the macronutrient-distribution nodes would have misrepresented the intervention. Evidence from the broader literature also suggests that diets enriched in MUFA may influence cardiometabolic outcomes independently of overall macronutrient balance, further supporting the evaluation of MUFA enrichment as a distinct dietary characteristic within the network [6].

MUFA enrichment was selected as the single quality marker following an exploratory classification process. Fibre content and sodium reduction were also considered as quality-based classification dimensions, as some included trials targeted these characteristics as primary intervention aims. However, when these markers were included alongside MUFA enrichment, the resulting network became too sparse to support meaningful comparative estimation. MUFA was therefore retained as the single quality marker as it generated sufficient network density while reflecting the most prevalent quality characteristic actively targeted as a primary intervention aim across the included studies. No included trial was specifically designed to investigate other quality dimensions, such as low saturated fat or low added sugars, as a primary dietary target independently of macronutrient distribution. Saturated fat in particular did not vary meaningfully between intervention arms and therefore could not serve as a discriminating classification criterion. The decision not to include such quality dimensions was therefore evidence-driven and reflects the structure of the available evidence rather than a judgement about their clinical importance.

The hMUFA node was defined as ≥20% of total daily energy intake from MUFA. This node did not overlap with any other dietary category. Although diets assigned to the other nodes varied in MUFA content, none reached the predefined threshold for hMUFA classification. Consequently, hMUFA interventions occupied a distinct region of dietary exposure space, and no intervention arm fulfilled the criteria for more than one node.

Node assignment was primarily based on the dietary targets defined by each intervention protocol. When data on achieved macronutrient intake were also available, adherence was additionally evaluated to determine whether participants’ actual intake remained consistent with the assigned dietary category. In cases where achieved intake deviated substantially from the prescribed targets to the extent that the intervention no longer fulfilled the predefined criteria for its original node, achieved intake was prioritised for node assignment. This approach was adopted to ensure that classifications reflected the dietary exposure actually experienced by participants rather than the intended intervention target alone, thereby reducing the risk of exposure misclassification and improving the clinical interpretability and validity of the network comparisons.

The dietary interventions included in this current NMA are the following (macronutrients are expressed as % of Daily Energy intake):

1. High-MUFA diet (hMUFA): ≥20% of DEI from MUFA, irrespective of total fat/carbohydrate/protein distribution,
2. Ketogenic (very-high-fat (VHF), very-low-carbohydrate (VLC)): fat >70%, carbohydrate ≤10%,
3. High-fat, low-carbohydrate (HFLC): fat 36-70%, carbohydrate 11-44%,
4. High-fat, moderate-carbohydrate (HFMC): fat 36-70%, carbohydrate 45-60%,
5. Moderate-fat, low-carbohydrate (MFLC): fat 31-35%, carbohydrate 11-44%,
6. Moderate-fat, moderate-carbohydrate (MFMC): fat 31-35%, carbohydrate 45-60%,
7. Low-fat, moderate-carbohydrate (LFMC): fat ≤30%, carbohydrate 45-60%,
8. Low-fat, high-carbohydrate (LFHC): fat ≤30%, carbohydrate >60%.

The LFMC diet was the most frequently used comparator and therefore selected as the reference diet to maximise network connectivity.

**Data synthesis and statistical analysis**

To synthesise evidence across multiple dietary interventions, network meta-analyses were conducted in R (version 4.5.1) [3] using a frequentist framework implemented in the netmeta package [4]. Random-effects models were fitted to account for between-study heterogeneity, with the between-study variance estimated using the DerSimonian-Laird method. While this estimator is widely used and supports comparability with existing literature, it may underestimate between-study variance when the number of studies per comparison is small, potentially resulting in confidence intervals that do not fully reflect uncertainty in the estimates, and therefore uncertainty around effect estimates and rankings should be interpreted accordingly. Statistical heterogeneity was quantified using Cochran’s Q and the I² statistic. Transitivity was assessed descriptively by examining the distribution of prespecified effect modifiers across intervention comparisons. These included age, baseline body mass index, baseline cardiometabolic status and comorbidities, medication use, co-interventions (including structured physical activity), and intervention duration. Global inconsistency was assessed using a design-by-treatment interaction model, which tests whether treatment effects differ systematically across study designs and decomposes Cochran’s Q into within-design heterogeneity and between-design inconsistency. Local inconsistency was assessed using node-splitting, which separates direct and indirect evidence for specific comparisons and formally tests their agreement; statistically significant discrepancies were interpreted as evidence of inconsistency.

For each outcome, relative treatment effects were estimated under a consistency model that jointly integrates direct and indirect evidence. Results are presented as mean differences (MDs) with corresponding 95% confidence intervals (CIs). Reference diets were selected based on the frequency of direct comparisons within each network, with the most used comparator serving as the reference node to maximise network connectivity. Multi-arm trials were analysed using standard procedures implemented in netmeta, which account for correlations between comparisons and preserve within-trial randomisation. No additional small-sample or variance-correcting methods were applied beyond those incorporated into the random-effects framework. Crossover trials were incorporated by extracting mean differences and standard errors from within-subject comparisons as reported by the primary studies, which inherently preserve the paired nature of the data and account for within-subject correlation, consistent with standard NMA methodology. Washout periods were reviewed for all included crossover trials as part of the risk of bias assessment using the additional RoB 2 domain for crossover designs and considered adequate to minimise potential carryover effects between intervention periods. When washout periods were not implemented, studies were included if the study design and outcome reporting suggested that carryover effects were unlikely to substantially influence the results. However, residual uncertainty regarding carryover effects cannot be excluded for crossover trials where washout periods were not explicitly reported.

All statistical conversions and calculations were conducted according to the recommendations of the Cochrane Handbook for Systematic Reviews of Interventions [5]. Relative ranking of interventions was summarised using P-scores, which reflect the probability that an intervention is among the most effective based on the combined evidence. For each outcome and classification system, network geometry was visualised using network plots, with node size proportional to the total sample size contributing to each intervention and edge thickness proportional to the number of direct comparisons.

To explore potential effect modification, random-effects meta-regression analyses were conducted using prespecified study-level covariates, including intervention duration (weeks), percentage of female participants, and presence of caloric restriction (yes/no). Each covariate was examined in separate single-covariate models, and regression coefficients with 95% CIs were reported. Potential small-study effects were explored using funnel plots and formal tests for asymmetry. Comparison-adjusted funnel plots were used to account for the multi-comparison structure of network meta-analysis, and Egger-type regression tests were applied to assess whether smaller studies reported systematically different effects. These analyses were exploratory in nature. Given the study-level covariates examined and the limited number of studies contributing to individual nodes, findings should be interpreted as hypothesis-generating rather than confirmatory and require verification in future research.

**Risk of bias and certainty of evidence**

The credibility of all pairwise treatment effect estimates was evaluated using the CINeMA framework, a structured approach based on the GRADE methodology and adapted for network meta-analysis of multiple simultaneous treatment comparisons, implemented through the CINeMA web application (https://cinema.med.auth.gr). CINeMA evaluates confidence across six domains, including within-study bias, reporting bias, indirectness, imprecision, heterogeneity, and incoherence. Each domain is rated as having no concerns, some concerns, or major concerns, and these judgments are combined to produce an overall confidence rating of high, moderate, low, or very low for each pairwise network estimate.

The percentage contribution matrix played a central role in assessing within-study bias and indirectness. This matrix quantifies the proportional contribution of each study to each network estimate, based on study precision, whether the evidence is direct or indirect, and the distance between comparisons within the network structure. Within-study bias was assessed using the revised Cochrane Risk of Bias 2 (RoB 2) tool. The risk-of-bias judgments on the study level were then weighted according to the contribution matrix. Indirectness was assessed by evaluating the relevance of each study to the review question in terms of population characteristics, intervention definitions, and outcome assessment. Reporting bias was assessed at the network level based on the funnel plot analyses. Imprecision was evaluated by comparing the 95% confidence interval for each network estimate against a pre-specified range of equivalence representing clinically unimportant differences.

Heterogeneity was assessed under the random-effects model by examining whether the 95% prediction interval, incorporating the common between-study heterogeneity variance (τ²) estimated across the network, was consistent with the confidence interval in its implications for clinical decisions, following the range of equivalence specified for each outcome. Incoherence was evaluated locally using the node-splitting approach for comparisons with both direct and indirect evidence, and globally using the design-by-treatment interaction model for indirect-only comparisons. For the global test, no concerns, some concerns, and major concerns were assigned for p-values >0.10, between 0.05-0.10, and <0.05, respectively. When no closed loops were present and the global test could not be computed, major concerns for incoherence were assigned by default.

Overall confidence ratings were derived by downgrading from high confidence according to domain-level judgments. To avoid double-counting, related methodological limitations were not downgraded independently and interrelated concerns across domains were consolidated into a single level of downgrading. The absence of direct evidence was not treated as an independent reason for downgrading. Indirect-only comparisons were assessed using the same domain-based criteria as direct or mixed evidence comparisons, with certainty determined by the quality and coherence of the contributing indirect evidence.

**Supplementary Table 1.** Search strategy performed on each database

**a. Search on Pubmed (Filters: 01/01/2013 – 30/10/2025, English)**

| (("trial"[Title/Abstract] OR "control trial"[Title/Abstract] OR "clinical trial"[Title/Abstract] OR "randomized control trial"[Title/Abstract] OR "non randomized control trial"[Title/Abstract] OR "cohort"[Title/Abstract] OR "prospective"[Title/Abstract] OR "longitudinal"[Title/Abstract] OR "case control"[Title/Abstract]) AND ("amyloid a"[Title/Abstract] OR "homocysteine"[Title/Abstract] OR ("c reactive protein"[Title/Abstract] OR "CRP"[Title/Abstract]) OR "inflammation"[Title/Abstract] OR ("homoeostatic model of assessment"[Title/Abstract] OR "HOMA"[Title/Abstract]) OR "QUICKI"[Title/Abstract] OR "quantitative insulin sensitivity index"[Title/Abstract] OR "hyperglycemia"[Title/Abstract] OR "blood sugar"[Title/Abstract] OR "hyperglycemia"[MeSH Terms] OR "glucose"[Title/Abstract] OR "insulin"[Title/Abstract] OR "HbA1c"[Title/Abstract] OR ("glycated hemoglobin"[MeSH Terms] OR ("glycated"[All Fields] AND "hemoglobin"[All Fields]) OR "glycated hemoglobin"[All Fields] OR "glycated hemoglobin a"[All Fields]) OR "hypertension"[Title/Abstract] OR "hyperlipidemia"[Title/Abstract] OR "hyperlipidemia"[Title/Abstract] OR "blood pressure"[Title/Abstract] OR "systolic pressure"[Title/Abstract] OR "diastolic pressure"[Title/Abstract] OR "triacylglycerols"[Title/Abstract] OR "triglyceride*"[Title/Abstract] OR "HDL"[Title/Abstract] OR "high density lipoprotein"[Title/Abstract] OR "LDL"[Title/Abstract] OR "low density lipoprotein"[Title/Abstract] OR "cholesterol"[Title/Abstract] OR "cholesterol, hdl"[MeSH Terms] OR "cholesterol, ldl"[MeSH Terms] OR "waist circumference"[Title/Abstract] OR "waist circumference"[MeSH Terms] OR "fat free mass"[Title/Abstract] OR "fat mass"[Title/Abstract] OR "weight"[Title/Abstract] OR ("body mass index"[Title/Abstract] OR "BMI"[Title/Abstract]) OR "body mass index"[MeSH Terms] OR "body composition"[Title/Abstract] OR "body composition"[MeSH Terms] OR "adiposity"[MeSH Terms] OR "adiposity"[Title/Abstract] OR "overweight"[MeSH Terms] OR "overweight"[Title/Abstract] OR "obesity"[Title/Abstract]) AND ("non alcoholic fatty liver disease"[Title/Abstract] OR "kidney disease"[Title/Abstract] OR "cardiovascular abnormalities"[MeSH Terms] OR "cardiovascular health"[Title/Abstract] OR "cardiometabolic health"[Title/Abstract] OR "metabolic syndrome"[Title/Abstract] OR "cardiovascular"[Title/Abstract] OR "heart disease"[Title/Abstract] OR "vascular disease"[Title/Abstract] OR "myocardial infarction"[Title/Abstract] OR "stroke"[Title/Abstract] OR "diabetes"[Title/Abstract]) AND ("dietary intake"[Title/Abstract] OR "eating practice*"[Title/Abstract] OR "diet group*"[Title/Abstract] OR "diet"[Title/Abstract] OR "diet"[MeSH Terms] OR "dietary pattern*"[Title/Abstract] OR "dietary intervention*"[Title/Abstract] OR "dietary behavior"[Title/Abstract] OR "diet therapy"[Title/Abstract] OR "feeding behavior"[Title/Abstract] OR "energy intake"[Title/Abstract] OR "nutritional status"[MeSH Terms] OR "nutritional sciences"[MeSH Terms] OR "nutrition"[Title/Abstract] OR "nutritional status"[Title/Abstract])) | **13,542** |
| --- | --- |

**b. Search on Scopus (Filters: 01/01/2013 –30/10/2025, English)**

| ( TITLE-ABS-KEY ( ( "dietary W/0 intervention" ) OR ( "dietary W/0 pattern" ) OR ( "diet W/5 group" ) OR ( "eating W/0 practice" ) OR ( "dietary W/0 intake" ) OR ( "nutritional W/0 status" ) OR ( "energy W/0 intake" ) OR ( "feeding W/0 behaviour" ) OR ( "dietary W/0 therapy" ) OR ( "dietary W/0 behaviour" ) OR ( diet ) ) AND TITLE-ABS-KEY ( ( "non PRE/0 alcoholic PRE/0 fatty PRE/0 liver PRE/0 disease" ) OR ( "kidney W/0 disease" ) OR ( "cardiovascular W/0 abnormalities" ) OR ( "cardiovascular W/0 health" ) OR ( "cardiometabolic W/0 health" ) OR ( "metabolic W/0 syndrome" ) OR ( "heart W/0 disease" ) OR ( "metabolic W/0 syndrome" ) OR ( "vascular W/0 disease" ) OR ( "myocardial W/0 infarction" ) OR ( stroke ) OR ( diabetes ) ) AND TITLE-ABS-KEY ( ( "amyloid W/0 a" ) OR ( homocysteine ) OR ( "c PRE/0 reactive PRE/0 protein" ) OR ( crp ) OR ( inflammation ) OR ( "homoeostatic PRE/0 model PRE/0 of PRE/0 assessment" ) OR ( homa ) OR ( quicki ) OR ( "quantitative PRE/0 insulin PRE/0 sensitivity PRE/0 index" ) OR ( hyperglycemia ) OR ( "blood W/0 sugar" ) OR ( hyperglycemia ) OR ( glucose ) OR ( insulin ) OR ( hba1c ) OR ( "glycated PRE/0 hemoglobin" ) OR ( hypertension ) OR ( hyperlipidemia ) OR ( "high PRE/0 blood PRE/0 pressure" ) OR ("systolic PRE/1 pressure") OR ("diastolic PRE/1 pressure") OR ( triacylglycerols ) OR ( triglyceride* ) OR ( hdl ) OR ( "high PRE/0 density PRE/0 lipoprotein" ) OR ( ldl ) OR ( "low PRE/0 density PRE/0 lipoprotein" ) OR ( cholesterol ) OR ( "waist PRE/0 circumference" ) OR ( "fat W/0 free W/0 mass" ) OR ( "fat W/0 mass" ) ] OR ( weight ) OR ( "body PRE/0 mass PRE/0 index" ) OR ( bmi ) OR ( "body W/0 composition" ) OR ( adiposity ) OR ( overweight ) OR ( obesity ) ) AND TITLE-ABS-KEY ( ( trial ) OR ( "control W/0 trial" ) OR ( "clinical W/0 trial" ) OR ( "randomized PRE/0 control PRE/0 trial" ) OR ( cohort ) OR ( prospective ) OR ( longitudinal ) OR ( "case PRE/0 control" ) ) ) AND ( LIMIT-TO ( SRCTYPE , "j" ) ) AND ( LIMIT-TO ( DOCTYPE , "ar" ) ) AND ( LIMIT-TO ( LANGUAGE , "English" ) ) | **15,790** |
| --- | --- |

**c. Search on Web of Science (Filters: 01/01/2013 – 30/10/2025, English)**

| (((TS=((dietary W/0 intervention) OR (dietary W/0 pattern) OR (diet W/5 group) OR (eating W/0 practice) OR (dietary W/0 intake) OR (nutritional W/0 status) OR (energy W/0 intake) OR (feeding W/0 behaviour) OR (dietary W/0 therapy) OR (dietary W/0 behaviour) OR (diet))) AND TS=((non PRE/0 alcoholic PRE/0 fatty PRE/0 liver PRE/0 disease) OR (kidney W/0 disease) OR (cardiovascular W/0 abnormalities) OR (cardiovascular W/0 health) OR (cardiometabolic W/0 health) OR (metabolic W/0 syndrome) OR (heart W/0 disease) OR (metabolic W/0 syndrome) OR (vascular W/0 disease) OR (myocardial W/0 infarction) OR (stroke) OR (diabetes))) AND TS=((amyloid W/0 a) OR (homocysteine) OR (c PRE/0 reactive PRE/0 protein) OR (CRP) OR (inflammation) OR (homoeostatic PRE/0 model PRE/0 of PRE/0 assessment) OR (HOMA) OR (QUICKI) OR (quantitative PRE/0 insulin PRE/0 sensitivity PRE/0 index) OR (hyperglycemia) OR (blood W/0 sugar) OR (hyperglycemia) OR (glucose) OR (insulin) OR (HbA1c) OR (glycated PRE/0 hemoglobin) OR (hypertension) OR (hyperlipidemia) OR (high PRE/0 blood PRE/0 pressure) OR (systolic PRE/1 pressure) OR (diastolic PRE/1 pressure) OR (triacylglycerols) OR (triglyceride) OR (HDL) OR (high PRE/0 density PRE/0 lipoprotein) OR (LDL) OR (low PRE/0 density PRE/0 lipoprotein) OR (cholesterol) OR (waist PRE/0 circumference) OR (fat W/0 free W/0 mass) OR (fat W/0 mass) OR (weight) OR (body PRE/0 mass PRE/0 index) OR (BMI) OR (body W/0 composition) OR (adiposity) OR (overweight) OR (obesity))) AND TS=((trial) OR (control W/10 trial) OR (clinical W/0 trial) OR (randomized PRE/0 control PRE/0 trial) OR (cohort) OR (prospective) OR (longitudinal) OR (case PRE/0 control)) | **7,371** |
| --- | --- |

**d. Search on CINHAL (Filters: 01/01/2013 – 30/10/2025, English)**

| AB ( (amyloid W/0 a) OR (homocysteine) OR (c PRE/0 reactive PRE/0 protein) OR (CRP) OR (inflammation) OR (homoeostatic PRE/0 model PRE/0 of PRE/0 assessment) OR (HOMA) OR (QUICKI) OR (quantitative PRE/0 insulin PRE/0 sensitivity PRE/0 index) OR (hyperglycemia) OR (blood W/0 sugar) OR (hyperglycemia) OR (glucose) OR (insulin) OR (HbA1c) OR (glycated PRE/0 hemoglobin) OR (hypertension) OR (diastolic PRE/1 pressure) OR (systolic PRE/1 pressure) OR (hyperlipidemia) OR (high PRE/0 blood PRE/0 pressure) OR (triacylglycerols) OR (triglyceride) OR (HDL) OR (high PRE/0 density PRE/0 lipoprotein) OR (LDL) OR (low PRE/0 density PRE/0 lipoprotein) OR (cholesterol) OR (waist PRE/0 circumference) OR (fat W/0 free W/0 mass) OR (fat W/0 mass) OR (weight) OR (body PRE/0 mass PRE/0 index) OR (BMI) OR (body W/0 composition) OR (adiposity) OR (overweight) OR (obesity) ) AND AB ( (dietary W/0 intervention) OR (dietary W/0 pattern) OR (diet W/5 group) OR (eating W/0 practice) OR (dietary W/0 intake) OR (nutritional W/0 status) OR (energy W/0 intake) OR (feeding W/0 behaviour) OR (dietary W/0 therapy) OR (dietary W/0 behaviour) OR (diet) ) AND AB ( (non PRE/0 alcoholic PRE/0 fatty PRE/0 liver PRE/0 disease) OR (kidney W/0 disease) OR (cardiovascular W/0 abnormalities) OR (cardiovascular W/0 health) OR (cardiometabolic W/0 health) OR (metabolic W/0 syndrome) OR (heart W/0 disease) OR (metabolic W/0 syndrome) OR (vascular W/0 disease) OR (myocardial W/0 infarction) OR (stroke) OR (diabetes) ) AND AB ( (trial) OR (control W/10 trial) OR (clinical W/0 trial) OR (randomized PRE/0 control PRE/0 trial) OR (cohort) OR (prospective) OR (longitudinal) OR (case PRE/0 control)) | **2,431** |
| --- | --- |

**e. ProQuest (Filters: 1/1/2013 - 30/10/2025, English)**

| (tiab((amyloid a) OR (homocysteine) OR (c reactive protein) OR (CRP) OR (inflammation) OR (homoeostatic model of assessment) OR (HOMA) OR (QUICKI) OR (quantitative insulin sensitivity index) OR (hyperglycemia) OR (blood sugar) OR (hyperglycemia) OR (glucose) OR (insulin) OR (HbA1c) OR (glycated hemoglobin) OR (hypertension) OR (diastolic pressure) OR (systolic pressure) OR (hyperlipidemia) OR (high blood pressure) OR (triacylglycerols) OR (triglyceride) OR (HDL) OR (high density lipoprotein) OR (LDL) OR (low density lipoprotein) OR (cholesterol) OR (waist circumference) OR (fat free mass) OR (fat mass) OR (weight) OR (body mass index) OR (BMI) OR (body composition) OR (adiposity) OR (overweight) OR (obesity)) AND tiab((dietary intervention) OR (dietary pattern) OR (diet group) OR (eating practice) OR (dietary intake) OR (nutritional status) OR (energy intake) OR (feeding behaviour) OR (dietary therapy) OR (dietary behaviour) OR (diet)) AND tiab((non alcoholic fatty liver disease) OR (kidney disease) OR (cardiovascular abnormalities) OR (cardiovascular health) OR (cardiometabolic health) OR (metabolic syndrome) OR (heart disease) OR (metabolic syndrome) OR (vascular disease) OR (myocardial infarction) OR (stroke) OR (diabetes)) AND tiab((trial) OR (control trial) OR (clinical trial) OR (randomized control trial) OR (cohort) OR (prospective) OR (longitudinal) OR (case control)))) | **5,354** |
| --- | --- |

**Supplementary Table 2.** Structural characteristics and assessment of statistical assumptions across outcome-specific networks

| **Outcome** | **Studies** | **Pairwise comparisons** | **Treatments** | **Designs** | **Heterogeneity**  **(between studies)** | | **Heterogeneity**  **(within designs)** | | | **Global**  **inconsistency** | | | **Funnel plot**  **assymetry** | |
| --- | --- | --- | --- | --- | --- | --- | --- | --- | --- | --- | --- | --- | --- | --- |
|  | **N** | **N** | **N** | **N** | **τ^2^** | **Ι^2^ (95% CI)** | **Q** | **degrees of freedom** | **p-value** | **Q** | **degrees of freedom** | **p-value** | **z** | **p-value** |
| **Body mass index** | 14 | 18 | 9 | 10 | 0 | 0%  (0.0%; 64.8%) | 3.2 | 4 | 0.96 | 2.57 | 4 | 0.63 | -1.29 | 0.20 |
| **Waist circumference** | 14 | 18 | 8 | 9 | 0 | 0%  (0.0%; 62.4%) | 4.72 | 5 | 0.45 | 1.34 | 4 | 0.85 | 0.13 | 0.90 |
| **Low-density lipoprotein** | 23 | 31 | 9 | 16 | 0.02 | 71%  (54.5%; 81.6%) | 32.9 | 7 | <0.001 | 20.7 | 12 | 0.06 | 1.94 | 0.05 |
| **High-density lipoprotein** | 23 | 31 | 9 | 16 | 0 | 0%  (0.0%; 48.0%) | 8.41 | 7 | 0.3 | 8.86 | 12 | 0.72 | 0.83 | 0.41 |
| **Total cholesterol** | 22 | 26 | 9 | 15 | 0.05 | 78.8%  (66.8%; 86.5%) | 75.6 | 16 | <0.001 | 10.5 | 9 | 0.32 | 0.95 | 0.34 |
| **Triglycerides** | 24 | 32 | 9 | 16 | 0.001 | 61.8%  (38.7%; 76.2%) | 28.3 | 8 | <0.001 | 17.2 | 12 | 0.14 | -0.19 | 0.85 |
| **Systolic blood pressure** | 17 | 23 | 7 | 10 | 1.64 | 40.3%  (0.0%; 67.6%) | 23.5 | 14 | 0.05 | 14.8 | 7 | 0.45 | -1.25 | 0.21 |
| **Diastolic blood pressure** | 17 | 23 | 7 | 10 | 0.24 | 17.7%  (0.0%; 54.8%) | 17.0 | 14 | 0.26 | 4.37 | 7 | 0.74 | -0.16 | 0.88 |
| **Fasting glucose** | 22 | 26 | 8 | 13 | 0.005 | 36.9%  (0.0%; 64.2%) | 26.9 | 17 | 0.06 | 1.97 | 8 | 0.98 | 1.62 | 0.11 |
| **Fasting insulin** | 19 | 23 | 8 | 12 | 0 | 0%  (0.0%; 53.6%) | 10.1 | 14 | 0.75 | 8.06 | 7 | 0.33 | -1.32 | 0.19 |

**Supplementary Table 3.** Risk of Bias results for parallel and crossover studies

**a. Parallel studies**

| **Reference** | **Randomization process** | **Deviations from intended interventions** | **Missing outcome data** | **Measurement of the outcome** | **Selection of the reported result** | **Overall Bias** |
| --- | --- | --- | --- | --- | --- | --- |
| Andersson et al., 2016 | Low | Low | Low | Low | Low | Low |
| Azadbakht et al., 2013 | Some concerns | Low | Low | Low | Low | Some concerns |
| Bajerska et al., 2018 | Low | Low | Low | Low | Low | Low |
| Duś-Żuchowska et al., 2018 | Low | Low | Low | Low | Low | Low |
| Bazzano et al., 2014 | Low | Low | Low | Low | Low | Low |
| Bergia et al., 2022 | Low | Low | Low | Low | Low | Low |
| Boers et al., 2014 | Some concerns | Low | Low | Low | Low | Some concerns |
| Chiu et al. 2017 | Low | Low | Low | Low | Low | Low |
| Buscemi et al., 2013 | Low | Some concerns | Low | Low | Low | Some concerns |
| Guo et al., 2022 | Some concerns | Some concerns | High | Some concerns | Low | High |
| Tricò et al., 2021 | Some concerns | Some concerns | Low | Low | Low | Some concerns |
| Veum et al., 2017 | Low | Low | Low | Low | Low | Low |
| Belanger et al., 2023 | Low | Low | Low | Low | Low | Low |
| Chiu et al. 2014 | Low | Low | Low | Low | Low | Low |
| Dorans et al., 2022 | Low | Low | Low | Low | Low | Low |
| Ebbeling et al., 2021 | Low | Low | Low | Low | Low | Low |
| Giacco et al., 2014 | Low | Some concerns | Low | Low | Low | Some concerns |
| Jackson et al., 2014 | Low | Some concerns | Low | Low | Low | Some concerns |
| Kim et al., 2017 | Some concerns | Some concerns | Low | Low | Low | Some concerns |
| Jenkins et al., 2014 | High | Some concerns | Low | Low | Low | High |
| Krishnan et al., 2018 | Low | Low | Low | Low | Low | Low |
| Kitabchi et al., 2013 | Low | Low | Low | Low | Low | Low |
| Krishnan et al., 2012 | Low | Low | Low | Low | Low | Low |
| Uusitupa et al., 2013 | Low | High | Low | Some concerns | Low | High |
| Mousavi et al., 2023 | Low | Low | Low | Low | Low | Low |
| Poulsen et al., 2014 | Low | Low | Low | Low | Low | Low |
| Raben et al., 2020 | Low | Some concerns | Low | Low | Some concerns | Some concerns |
| Fechner et al., 2020 | Low | Low | Low | Low | Low | Low |
| Pinsawas et al., 2024 | Low | Low | Low | Low | Low | Low |
| Stomby et al., 2014 | Low | Low | Low | Some concerns | Low | Some concerns |
| Mellberg et al., 2014 | Low | Low | Low | Some concerns | Low | Some concerns |
| Hill et al., 2015 | Low | Low | Low | Low | Low | Low |
| Ruth et al., 2014 | Low | Low | Low | Low | Low | Low |
| Wycherley et al., 2014 | Low | Low | Low | Low | Low | Low |

**b. Crossover studies**

| **Reference** | **Randomization process** | **Period and carryover effects** | **Deviations from intended interventions** | **Missing outcome data** | **Measurement of the outcome** | **Selection of the reported result** | **Overall Bias** |
| --- | --- | --- | --- | --- | --- | --- | --- |
| Anderson-Vasquez et al., 2015 | Some concerns | High | Low | Low | Some concerns | Low | High |
| Brassard et al., 2017 | Low | Low | Low | Low | Low | Some concerns | Some concerns |
| Gadgil et al., 2013 | Low | Low | Low | Low | Low | Low | Low |
| Juraschek et al., 2013 | Low | Low | Low | Low | Low | Low | Low |
| Schroeder et al., 2015 | Low | Low | Low | Low | Low | Low | Low |
| Kirwan et al., 2016 | Low | Low | Low | Low | Low | Low | Low |
| Rajaie et al., 2014 | Low | Low | Low | Low | Low | Low | Low |
| Rajaie et al., 2013 | Low | Low | Low | Low | Low | Low | Low |
| Sacks et al., 2014 | Low | Low | Low | Low | Low | Low | Low |
| von Frankenberg et al., 2015 | Low | Low | Some concerns | Low | High | Low | High |
| Marina et al., 2014 | Low | Low | Low | Low | Some concerns | Low | Some concerns |
| Waliłko et al., 2021 | Low | Low | Low | Low | Low | Low | Low |

**Supplementary Table 4.** Confidence in Network Meta-Analysis (CINeMA) results per outcome and comparison

**Number of studies** refers to the number of studies with direct comparisons only. **Certainty** ratings reflect the full network contribution including indirect evidence. **Abbreviations:** HFLC: high-fat—low-carbohydrate; LFMC: low-fat—moderate-carbohydrate; LFLC: low-fat—low-carbohydrate; MFLC: moderate-fat—low-carbohydrate; MFMC: moderate-fat—moderate-carbohydrate; HFMC: high-fat—moderate-carbohydrate; LFHC: low-fat—high-carbohydrate; hMUFA: high-monounsaturated fatty acid

**a.** CINeMA results for body mass index

| **Comparison** | **Number of studies** | **Within-study bias** | **Reporting bias** | **Indirectness** | **Imprecision** | **Heterogeneity** | **Incoherence** | **Confidence rating** | **Reason(s) for downgrading** |
| --- | --- | --- | --- | --- | --- | --- | --- | --- | --- |
| HFLC : LFMC | 5 | Major concerns | Low risk | No concerns | No concerns | No concerns | No concerns | Low | Within-study bias |
| Ketogenic : LFMC | 1 | No concerns | Low risk | No concerns | Major concerns | No concerns | No concerns | Low | Imprecision |
| LFLC : LFMC | 1 | No concerns | Low risk | No concerns | Major concerns | No concerns | No concerns | Low | Imprecision |
| LFMC : MFLC | 1 | No concerns | Low risk | No concerns | No concerns | No concerns | No concerns | High | – |
| LFMC : MFMC | 2 | No concerns | Low risk | No concerns | Major concerns | No concerns | No concerns | Low | Imprecision |
| hMUFA : LFMC | 3 | No concerns | Low risk | No concerns | No concerns | Some concerns | No concerns | Moderate | Heterogeneity |
| HFMC : LFMC | 0 | Some concerns | Low risk | No concerns | Major concerns | No concerns | No concerns | Low | Imprecision |
| LFHC : LFMC | 0 | Some concerns | Low risk | No concerns | Some concerns | Some concerns | No concerns | Moderate | Imprecision, Heterogeneity |

**b.** CINeMA results for waist circumference

| **Comparison** | **Number of studies** | **Within-study bias** | **Reporting bias** | **Indirectness** | **Imprecision** | **Heterogeneity** | **Incoherence** | **Confidence rating** | **Reason(s) for downgrading** |
| --- | --- | --- | --- | --- | --- | --- | --- | --- | --- |
| HFLC:LFMC | 5 | Some concerns | Low risk | No concerns | Major concerns | No concerns | No concerns | Low | Within-study bias; Imprecision |
| Ketogenic:LFMC | 1 | No concerns | Low risk | No concerns | Some concerns | Some concerns | No concerns | Moderate | Imprecision; Heterogeneity |
| LFMC:MFLC | 1 | No concerns | Low risk | No concerns | Major concerns | No concerns | No concerns | Low | Imprecision |
| LFMC:MFMC | 3 | No concerns | Low risk | No concerns | No concerns | No concerns | No concerns | High | — |
| hMUFA:LFMC | 3 | No concerns | Low risk | No concerns | Major concerns | No concerns | No concerns | Low | Imprecision |
| HFMC:LFMC | 0 | Some concerns | Low risk | No concerns | Major concerns | No concerns | No concerns | Low | Within-study bias; Imprecision |
| LFHC:LFMC | 0 | Some concerns | Low risk | No concerns | Major concerns | No concerns | No concerns | Low | Within-study bias; Imprecision |

**c.** CINeMA results for low-density lipoprotein cholesterol

| **Comparison** | **Number of studies** | **Within-study bias** | **Reporting bias** | **Indirectness** | **Imprecision** | **Heterogeneity** | **Incoherence** | **Confidence rating** | **Reason(s) for downgrading** |
| --- | --- | --- | --- | --- | --- | --- | --- | --- | --- |
| HFLC:LFMC | 7 | Some concerns | Low risk | No concerns | No concerns | Major concerns | No concerns | Low | Within-study bias; Heterogeneity |
| Ketogenic:LFMC | 1 | No concerns | Low risk | No concerns | Some concerns | Some concerns | No concerns | Moderate | Imprecision; Heterogeneity |
| LFHC:LFMC | 1 | No concerns | Low risk | No concerns | Some concerns | No concerns | No concerns | Moderate | Imprecision |
| LFLC:LFMC | 1 | No concerns | Low risk | No concerns | Major concerns | No concerns | No concerns | Low | Imprecision |
| LFMC:MFLC | 1 | No concerns | Low risk | No concerns | Some concerns | Some concerns | No concerns | Moderate | Imprecision; Heterogeneity |
| LFMC:MFMC | 4 | No concerns | Low risk | No concerns | No concerns | Some concerns | No concerns | Moderate | Heterogeneity |
| hMUFA:LFMC | 5 | No concerns | Low risk | No concerns | No concerns | Major concerns | No concerns | Low | Heterogeneity |
| HFMC:LFMC | 0 | Some concerns | Low risk | No concerns | Some concerns | Some concerns | No concerns | Moderate | Within-study bias; Imprecision; Heterogeneity |

**d.** CINeMA results for high-density lipoprotein cholesterol

| **Comparison** | **Number of studies** | **Within-study bias** | **Reporting bias** | **Indirectness** | **Imprecision** | **Heterogeneity** | **Incoherence** | **Confidence rating** | **Reason(s) for downgrading** |
| --- | --- | --- | --- | --- | --- | --- | --- | --- | --- |
| HFLC:LFMC | 7 | No concerns | Low risk | No concerns | No concerns | No concerns | No concerns | High | — |
| Ketogenic:LFMC | 1 | No concerns | Low risk | No concerns | No concerns | No concerns | No concerns | High | — |
| LFHC:LFMC | 1 | No concerns | Low risk | No concerns | No concerns | No concerns | No concerns | High | — |
| LFLC:LFMC | 1 | No concerns | Low risk | No concerns | Some concerns | No concerns | No concerns | Moderate | Imprecision |
| LFMC:MFLC | 1 | No concerns | Low risk | No concerns | No concerns | No concerns | No concerns | High | — |
| LFMC:MFMC | 4 | No concerns | Low risk | No concerns | No concerns | No concerns | No concerns | High | — |
| hMUFA:LFMC | 5 | No concerns | Low risk | No concerns | No concerns | No concerns | No concerns | High | — |
| HFMC:LFMC | 0 | Some concerns | Low risk | No concerns | No concerns | No concerns | No concerns | Moderate | Within-study bias |

**e.** CINeMA results for total cholesterol

| **Comparison** | **Number of studies** | **Within-study bias** | **Reporting bias** | **Indirectness** | **Imprecision** | **Heterogeneity** | **Incoherence** | **Confidence rating** | **Reason(s) for downgrading** |
| --- | --- | --- | --- | --- | --- | --- | --- | --- | --- |
| HFLC:LFMC | 6 | No concerns | Low risk | No concerns | No concerns | Major concerns | No concerns | Low | Heterogeneity |
| Ketogenic:LFMC | 1 | No concerns | Low risk | No concerns | No concerns | Some concerns | Some concerns | Moderate | Heterogeneity; Incoherence |
| LFLC:LFMC | 1 | No concerns | Low risk | No concerns | Major concerns | No concerns | No concerns | Low | Imprecision |
| LFMC:MFMC | 4 | No concerns | Low risk | No concerns | No concerns | Some concerns | No concerns | Moderate | Heterogeneity |
| hMUFA:LFMC | 4 | No concerns | Low risk | No concerns | Some concerns | Some concerns | No concerns | Moderate | Imprecision; Heterogeneity |
| HFMC:LFMC | 0 | Some concerns | Low risk | No concerns | Major concerns | No concerns | No concerns | Low | Within-study bias; Imprecision |
| LFHC:LFMC | 0 | No concerns | Low risk | No concerns | Major concerns | No concerns | No concerns | Low | Imprecision |
| LFMC:MFLC | 0 | No concerns | Low risk | No concerns | Major concerns | No concerns | No concerns | Low | Imprecision |

**f.** CINeMA results for triglycerides

| **Comparison** | **Number of studies** | **Within-study bias** | **Reporting bias** | **Indirectness** | **Imprecision** | **Heterogeneity** | **Incoherence** | **Confidence rating** | **Reason(s) for downgrading** |
| --- | --- | --- | --- | --- | --- | --- | --- | --- | --- |
| HFLC:LFMC | 7 | No concerns | Low risk | No concerns | No concerns | Some concerns | No concerns | Moderate | Heterogeneity |
| Ketogenic:LFMC | 1 | No concerns | Low risk | No concerns | No concerns | Some concerns | Major concerns | Low | Heterogeneity; Incoherence |
| LFHC:LFMC | 1 | No concerns | Low risk | No concerns | No concerns | Some concerns | No concerns | Moderate | Heterogeneity |
| LFLC:LFMC | 2 | No concerns | Low risk | No concerns | No concerns | No concerns | No concerns | High | — |
| LFMC:MFLC | 1 | No concerns | Low risk | No concerns | Some concerns | No concerns | No concerns | Moderate | Imprecision |
| LFMC:MFMC | 4 | No concerns | Low risk | No concerns | No concerns | No concerns | No concerns | High | — |
| hMUFA:LFMC | 5 | No concerns | Low risk | No concerns | No concerns | Some concerns | No concerns | Moderate | Heterogeneity |
| HFMC:LFMC | 0 | Some concerns | Low risk | No concerns | No concerns | Some concerns | No concerns | Moderate | Within-study bias; Heterogeneity |

**g.** CINeMA results for systolic blood pressure

| **Comparison** | **Number of studies** | **Within-study bias** | **Reporting bias** | **Indirectness** | **Imprecision** | **Heterogeneity** | **Incoherence** | **Confidence rating** | **Reason(s) for downgrading** |
| --- | --- | --- | --- | --- | --- | --- | --- | --- | --- |
| HFLC:LFMC | 6 | No concerns | Low risk | No concerns | Some concerns | Some concerns | No concerns | Moderate | Imprecision; Heterogeneity |
| LFHC:LFMC | 1 | No concerns | Low risk | No concerns | Major concerns | No concerns | No concerns | Low | Imprecision |
| LFLC:LFMC | 1 | No concerns | Low risk | No concerns | Major concerns | No concerns | Major concerns | Low | Imprecision; Incoherence |
| LFMC:MFMC | 4 | No concerns | Low risk | No concerns | No concerns | Major concerns | No concerns | Low | Heterogeneity |
| hMUFA:LFMC | 5 | No concerns | Low risk | No concerns | Major concerns | No concerns | No concerns | Low | Imprecision |
| Ketogenic:LFMC | 0 | No concerns | Low risk | No concerns | Major concerns | No concerns | Major concerns | Low | Imprecision; Incoherence |

**h.** CINeMA results for diastolic blood pressure

| **Comparison** | **Number of studies** | **Within-study bias** | **Reporting bias** | **Indirectness** | **Imprecision** | **Heterogeneity** | **Incoherence** | **Confidence rating** | **Reason(s) for downgrading** |
| --- | --- | --- | --- | --- | --- | --- | --- | --- | --- |
| HFLC : LFMC | 6 | No concerns | Low risk | No concerns | No concerns | Major concerns | No concerns | Low | Heterogeneity |
| LFHC : LFMC | 1 | No concerns | Low risk | No concerns | Major concerns | No concerns | No concerns | Low | Imprecision |
| LFLC : LFMC | 1 | No concerns | Low risk | No concerns | Major concerns | No concerns | No concerns | Low | Imprecision |
| LFMC : MFMC | 4 | No concerns | Low risk | No concerns | Major concerns | No concerns | No concerns | Low | Imprecision |
| hMUFA : LFMC | 5 | No concerns | Low risk | No concerns | Major concerns | No concerns | No concerns | Low | Imprecision |
| Ketogenic : LFMC | 0 | No concerns | Low risk | No concerns | Major concerns | No concerns | No concerns | Low | Imprecision |

**i.** CINeMA results for fasting glucose

| **Comparison** | **Number of studies** | **Within-study bias** | **Reporting bias** | **Indirectness** | **Imprecision** | **Heterogeneity** | **Incoherence** | **Confidence rating** | **Reason(s) for downgrading** |
| --- | --- | --- | --- | --- | --- | --- | --- | --- | --- |
| HFLC:LFMC | 6 | Some concerns | Low risk | No concerns | No concerns | No concerns | No concerns | Moderate | Within-study bias |
| Ketogenic:LFMC | 1 | No concerns | Low risk | No concerns | Some concerns | No concerns | No concerns | Moderate | Imprecision |
| LFHC:LFMC | 1 | No concerns | Low risk | No concerns | No concerns | Some concerns | No concerns | Moderate | Heterogeneity |
| LFLC:LFMC | 1 | No concerns | Low risk | No concerns | Some concerns | No concerns | No concerns | Moderate | Imprecision |
| LFMC:MFMC | 4 | No concerns | Low risk | No concerns | No concerns | No concerns | No concerns | High | — |
| hMUFA:LFMC | 5 | No concerns | Low risk | No concerns | No concerns | No concerns | No concerns | High | — |
| HFMC:LFMC | 0 | Some concerns | Low risk | No concerns | Major concerns | No concerns | No concerns | Low | Within-study bias; Imprecision |

**j.** CINeMA results for fasting insulin

| **Comparison** | **Number of studies** | **Within-study bias** | **Reporting bias** | **Indirectness** | **Imprecision** | **Heterogeneity** | **Incoherence** | **Confidence rating** | **Reason(s) for downgrading** |
| --- | --- | --- | --- | --- | --- | --- | --- | --- | --- |
| HFLC:LFMC | 5 | Some concerns | Low risk | No concerns | Some concerns | Some concerns | Major concerns | Low | Within-study bias; Imprecision; Heterogeneity; Incoherence |
| Ketogenic:LFMC | 1 | No concerns | Low risk | No concerns | Major concerns | No concerns | No concerns | Low | Imprecision |
| LFHC:LFMC | 1 | No concerns | Low risk | No concerns | Major concerns | No concerns | No concerns | Low | Imprecision |
| LFMC:MFMC | 4 | No concerns | Low risk | No concerns | No concerns | Some concerns | No concerns | Moderate | Heterogeneity |
| hMUFA:LFMC | 4 | No concerns | Low risk | No concerns | Major concerns | No concerns | No concerns | Moderate | Imprecision |
| HFMC:LFMC | 0 | No concerns | Low risk | No concerns | Major concerns | No concerns | No concerns | Low | Imprecision |
| LFMC:MFLC | 0 | No concerns | Low risk | No concerns | Major concerns | No concerns | No concerns | Low | Imprecision |

**Supplementary Table 5.** Results of the meta-regression analyses performed for all outcomes

| **INTERVENTION DURATION (weeks)** | | | | | | | | | | |
| --- | --- | --- | --- | --- | --- | --- | --- | --- | --- | --- |
| **Diet** | **BMI** | **WC** | **LDL-C** | **HDL-C** | **TC** | **TG** | **SBP** | **DBP** | **FG** | **FI** |
| HFLC | 0.005  (-0.02; 0.03) | -0.01  (-0.08; 0.06 | -0.006  (-0.02; 0.01) | 0.0002  (-0.002; 0.002) | -0.01  (-0.03; 0.02) | 0.001  (-0.02; 0.02) | 0.05  (-0.04; 0.13) | 0.02  (-0.03; 0.07) | 0.01  ( 0.001; 0.01) | 0.01  (-0.02; 0.02) |
| HFMC | 0.76  (-0.64; 2.15) | N/A | -0.04  (-0.60; 0.52) | 0.03  (-0.08; 0.14) | -0.06  (-0.82; 0.71) | 0.09  (-0.49; 0.68) | N/A | N/A | -0.02  (-0.35; 0.31) | -0.01  (-0.03; 0.01) |
| hMUFA | N/A | N/A | 0.003  (-0.005; 0.01) | -0.001  (-0.002; 0.0008) | 0.003  (-0.01; 0.02) | 0.002  (-0.01; 0.01) | -0.01  (-0.11; 0.10) | -0.03  (-0.08; 0.03) | -0.0003  (-0.01; 0.01) | -0.29  (-1.15; 0.60) |
| LFHC | N/A | N/A | 0.15  (0.0004; 0.30) | -0.02  (-0.06; 0.02) | 0.15  (-0.05; 0.36) | 0.07  (-0.10; 0.24) | N/A | N/A | 0.03  (-0.05; 0.10) | 0.26  (-0.70; 1.22 |
| MFMC | N/A | 0.12  (-0.12; 0.35 | -0.01  (-0.05; 0.02) | -0.002  (-0.006; 0.002) | -0.01  (-0.06; 0.04) | -0.001  (-0.05; 0.05) | 0.03  (-0.24; 0.30) | 0.14  (-0.04; 0.32) | 0.01  (0.01; 0.02) | -0.11  (-0.21; -0.003) |
| LFLC | N/A | N/A | N/A | N/A | N/A | 0.04  (-0.01; 0.09) | N/A | N/A | N/A | N/A |
| **FEMALE PARTICIPANTS (percentage)** | | | | | | | | | | |
| **Diet** | **BMI** | **WC** | **LDL-C** | **HDL-C** | **TC** | **TG** | **SBP** | **DBP** | **FG** | **FI** |
| HFLC | -1.24  (-4.67; 2.20) | -6.6  (-15.09; 1.81) | 0.55  (-0.12; 1.22) | 0.02  (-0.06; 0.10) | 1.21  (-0.26; 2.67) | 0.13  (-0.87; 1.13) | -0.74  (-4.38; 2.90) | 1.11  (-1.27; 3.49) | -0.53  (-1.12; 0.06) | 1.61  (-2.55; 5.77) |
| Ketogenic | -1.71  (-4.85; 1.44) | -1.15  (-8.11; 5.81) | -1.20  (-2.07; -0.33) | -0.06  (-0.24; 0.12) | -1.16  (-2.34; 0.02) | -0.64  (-1.54; 0.26) | N/A | N/A | -0.25  (-0.96; 0.45) | -0.73  (-8.36; 6.89) |
| hMUFA | N/A | N/A | 1.19  (0.42; 1.96) | -0.008  (-0.15; 0.13) | 1.75  (0.36; 3.14) | 0.87  (-0.24; 1.97) | 1.76  (-6.43; 9.94) | 1.19  (-4.00; 6.39) | -0.05  (-0.53; 0.43) | -0.87  (-3.07; 1.33) |
| LFHC | N/A | N/A | 1.78  (0.43; 3.13) | -0.16  (-0.58; 0.26) | 2.43  (0.42; 4.43) | 0.81  ( -0.90; 2.52) | N/A | N/A | 0.07 (-0.98; 1.11) | 3.25  (-17.0; 23.5) |
| MFMC | N/A | -11.52  (-27.07; 4.03) | 0.11  (-1.0077; 1.23) | -0.21  (-0.49; 0.06) | 0.30  (-1.49; 2.08) | 0.36  (-2.32; 3.03) | -4.17  (-19.41;11.1) | -8.47  (-18.5; 1.57) | -0.96  (-1.80; -0.11) | -3.91  (-14.09; 6.27) |
| LFLC | N/A | N/A | N/A | N/A | N/A | 7.02  (0.55; 13.5) | N/A | N/A | N/A | N/A |
| **REDUCTION OF CALORIES (Yes/No)** | | | | | | | | | | |
| **Diet** | **BMI** | **WC** | **LDL-C** | **HDL-C** | **TC** | **TG** | **SBP** | **DBP** | **FG** | **FI** |
| HFLC | 0.03  (-0.49; 0.56) | 0.70  (-1.59; 3.00) | -0.05  (-0.42; 0.31) | 0.008  (-0.03; 0.05) | 0.11  (-0.52; 0.73) | 0.14  (-0.39; 0.66) | -0.70  (-2.38; 0.97) | 0.54  (-0.53; 1.60) | -0.01  (-0.27; 0.25) | 0.005  (-0.77; 0.78) |
| HFMC | 0.51  (-1.27; 4.29) | N/A | 1.21  (-0.13; 2.55) | 0.006  (-0.20; 0.21) | 1.24  (-0.61; 3.09) | 0.84  (-0.59; 2.27) | N/A | N/A | 0.27  (-0.74; 1.29) | N/A |
| hMUFA | N/A | N/A | 0.32  (-0.30; 0.94) | 0.04  (-0.07; 0.14) | 0.47  (-0.49; 1.43) | 0.5  (-0.35; 1.35) | 1.47  (-3.49; 6.44) | 2.27  (-1.06; 5.61) | 0.09  (-0.36; 0.53) | 0.86  (-1.03; 2.74) |
| LFHC | N/A | N/A | 1.59  (0.43; 2.75) | -0.10  (-0.42; 0.21) | 1.66  (0.04; 3.28) | 0.79  (-0.56; 2.14) | N/A | N/A | 0.36  (-0.47; 1.19) | 2.96  (-4.90; 10.8) |
| MFMC | N/A | 1.26  (-4.03; 6.54) | 0.78  (-0.08; 1.64) | 0.07  (-0.07; 0.22) | 0.70  (-0.56; 1.96) | 0.43  (-0/8; 1.66) | N/A | N/A | 0.25  (-0.51; 1.01) | -0.05  (-6.56; 6.46) |

**Abbreviations:** BMI, body mass index; WC, waist circumference; LDL-C, low-density lipoprotein cholesterol; HDL-C, high-density lipoprotein cholesterol; TC, total cholesterol; TG, triglycerides; SBP, systolic blood pressure; DBP, diastolic blood pressure; FG, fasting glucose; FI, fasting insulin; HFLC, high-fat—low-carbohydrate; LFMC, low-fat—moderate-carbohydrate; Ketogenic, ketogenic diet; LFHC, low-fat—high-carbohydrate; MFMC, moderate-fat—moderate-carbohydrate; MFLC, moderate-fat—low-carbohydrate; hMUFA, high-monounsaturated fatty acid; HFMC, high-fat—moderate-carbohydrate; N/A, not available as regression coefficients were estimate only for treatment nodes with sufficient comparative information relative to the reference intervention LFMC

**Supplementary Figure 1.** Prisma network meta-analysis flowchart

**
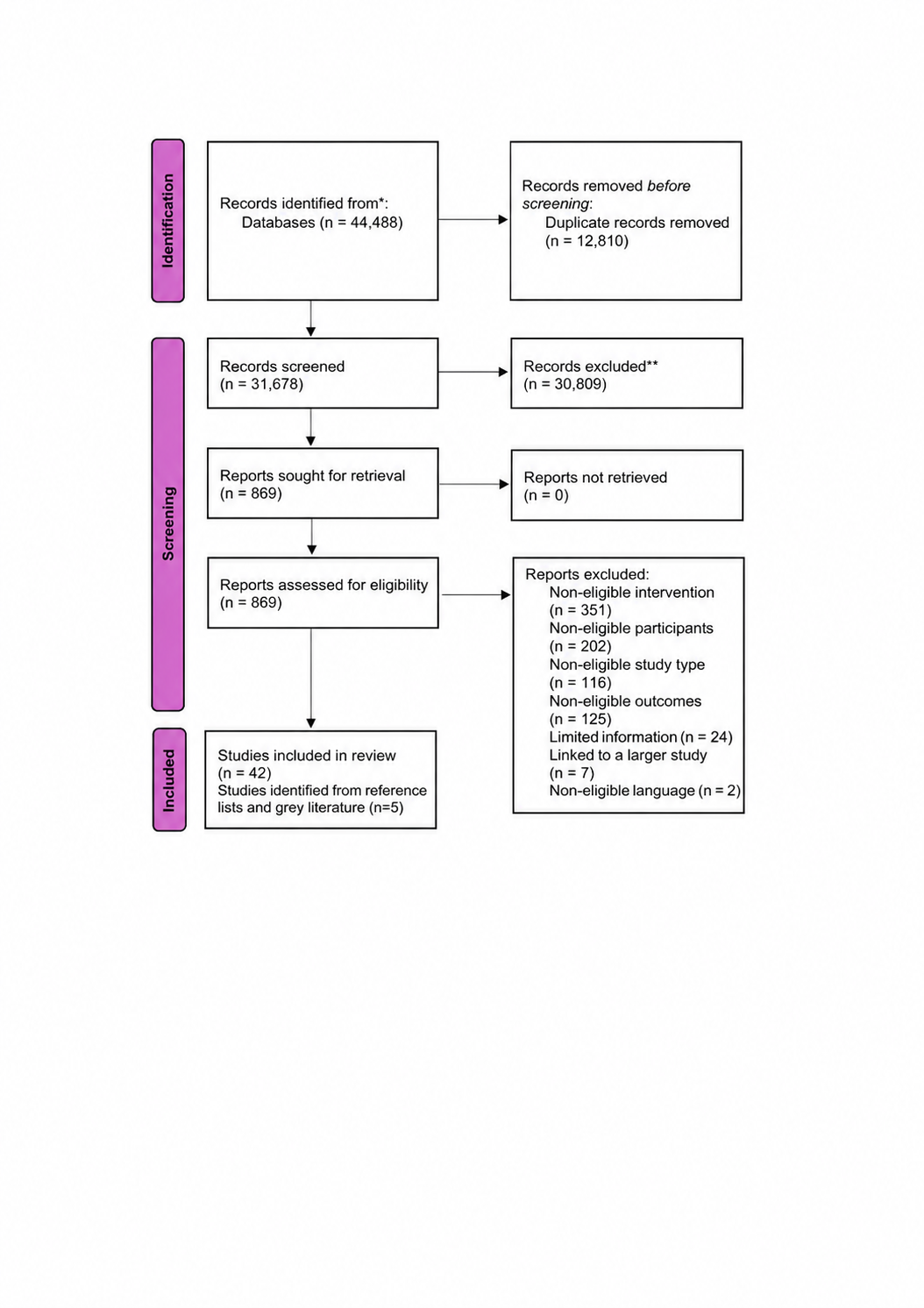
**

**Supplementary Figure 2.** Network plots for a) body mass index (BMI), and b) waist circumference (WC)

**
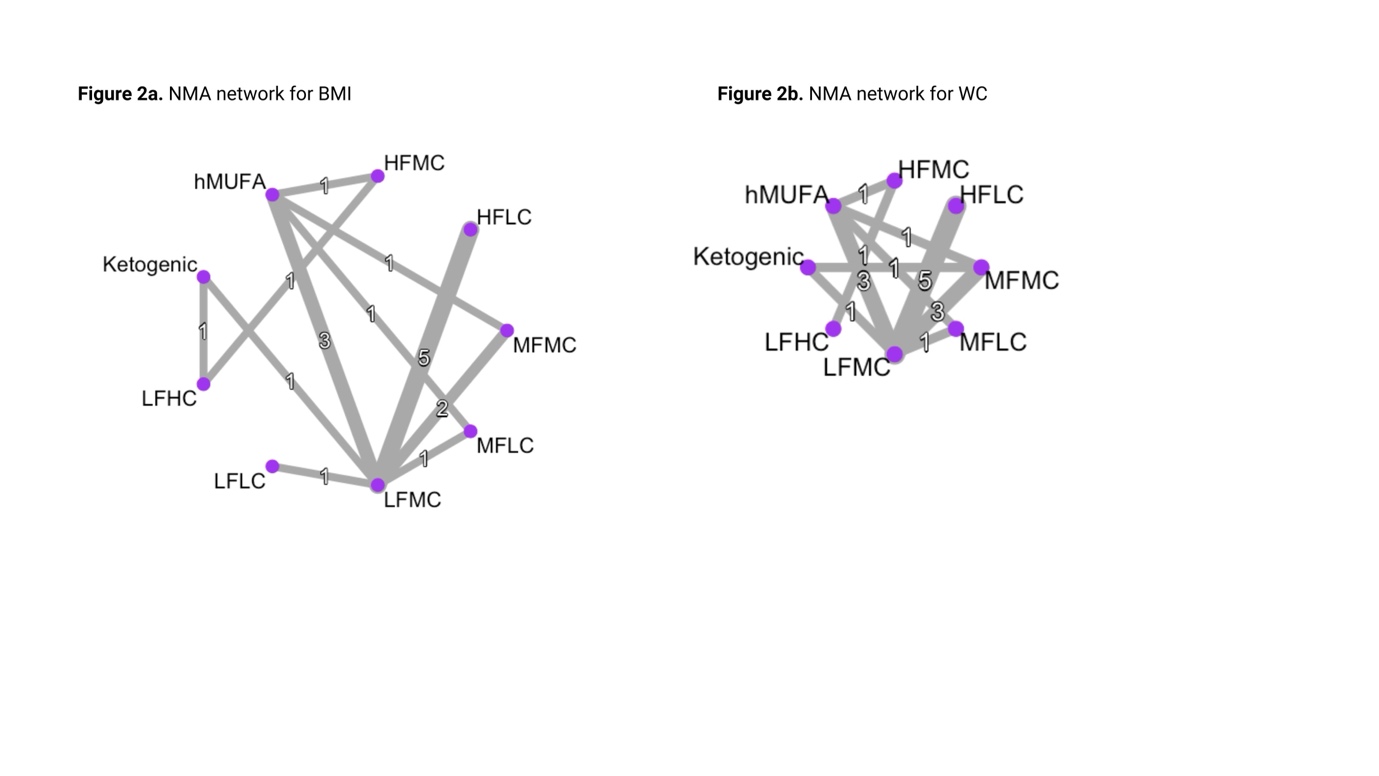
**

**Supplementary Figure 3.** Network plots for a) low-density lipoprotein cholesterol (LDL), b) high-density lipoprotein cholesterol (HDL), c) total cholesterol (TC), and d) triglycerides (TG)


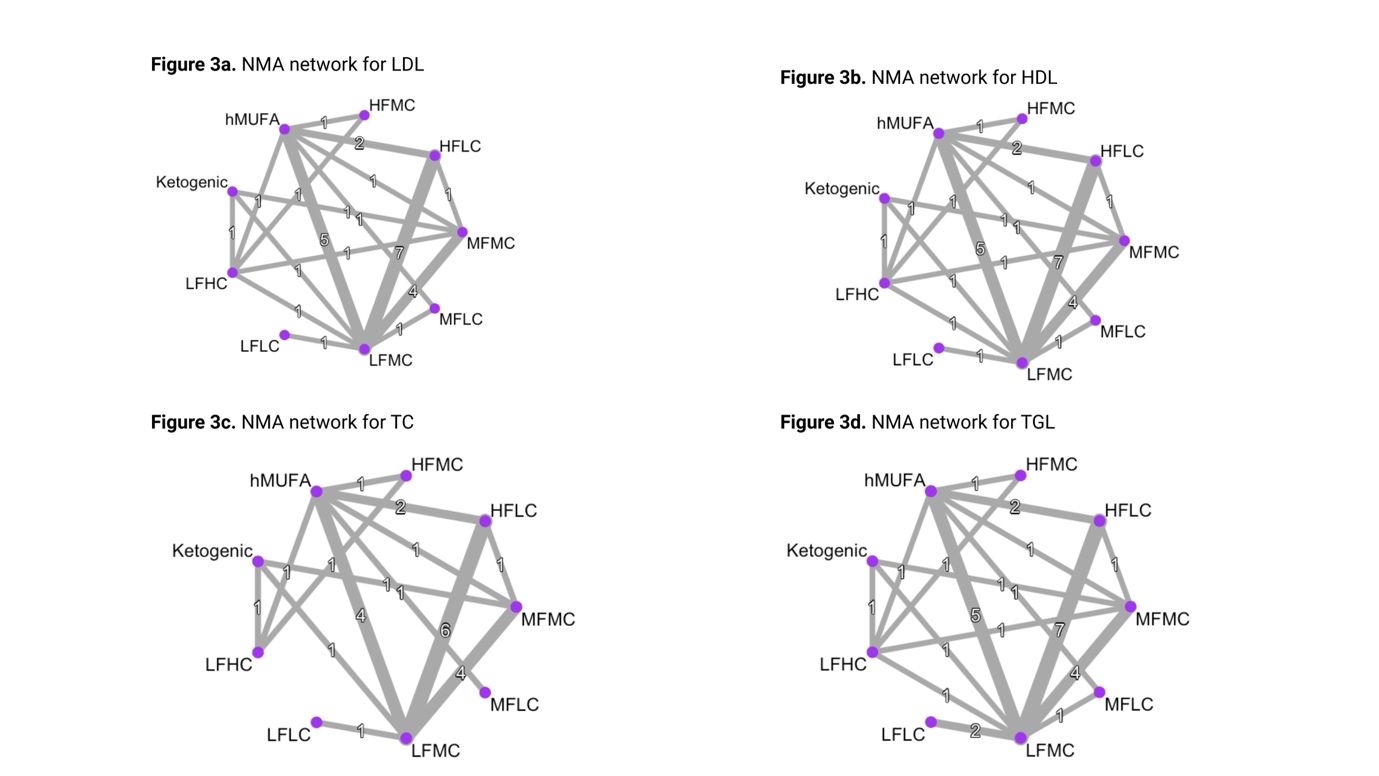


**Supplementary Figure 4.** Network plots for a) systolic (SBP), and b) diastolic (DBP) blood pressure

**
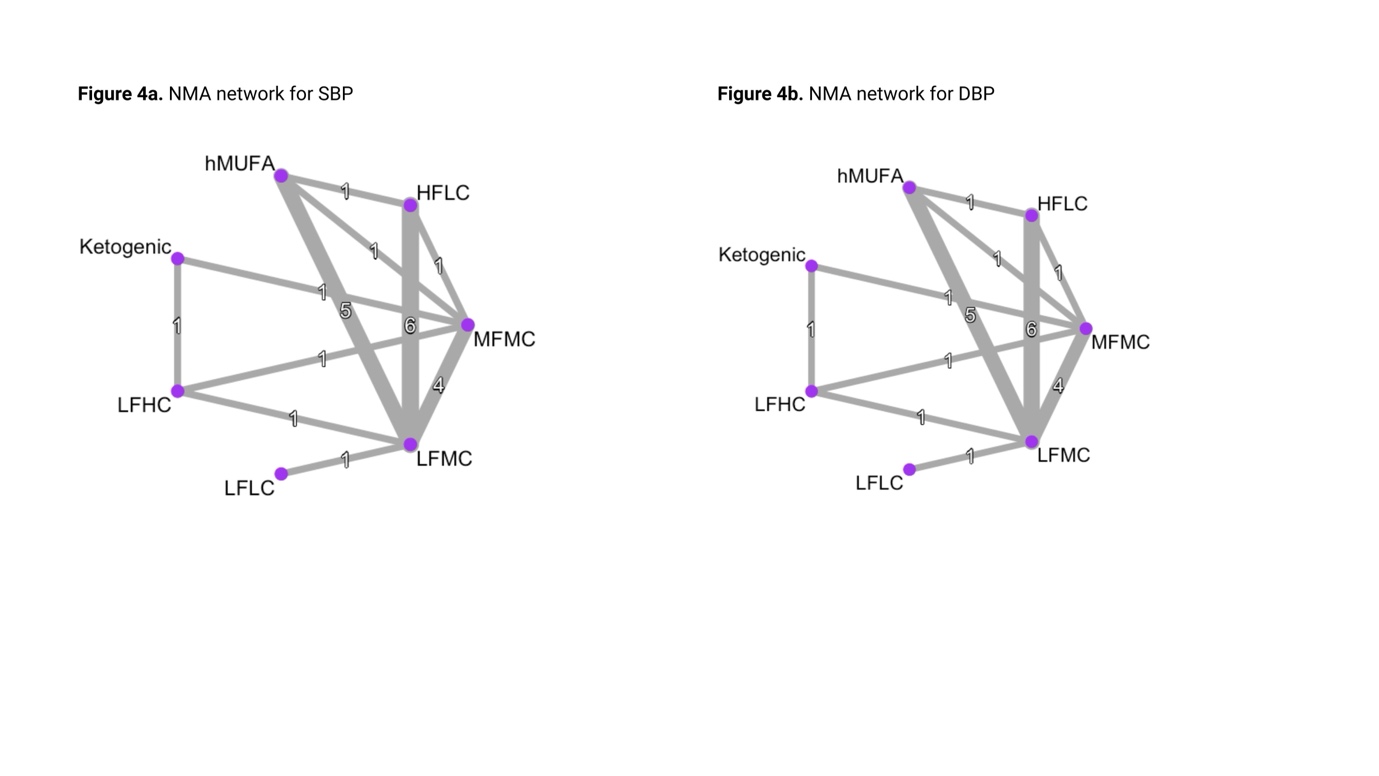
**

**Supplementary Figure 5.** Network plots for a) fasting glucose (FG), and b) fasting insulin (FI)

**
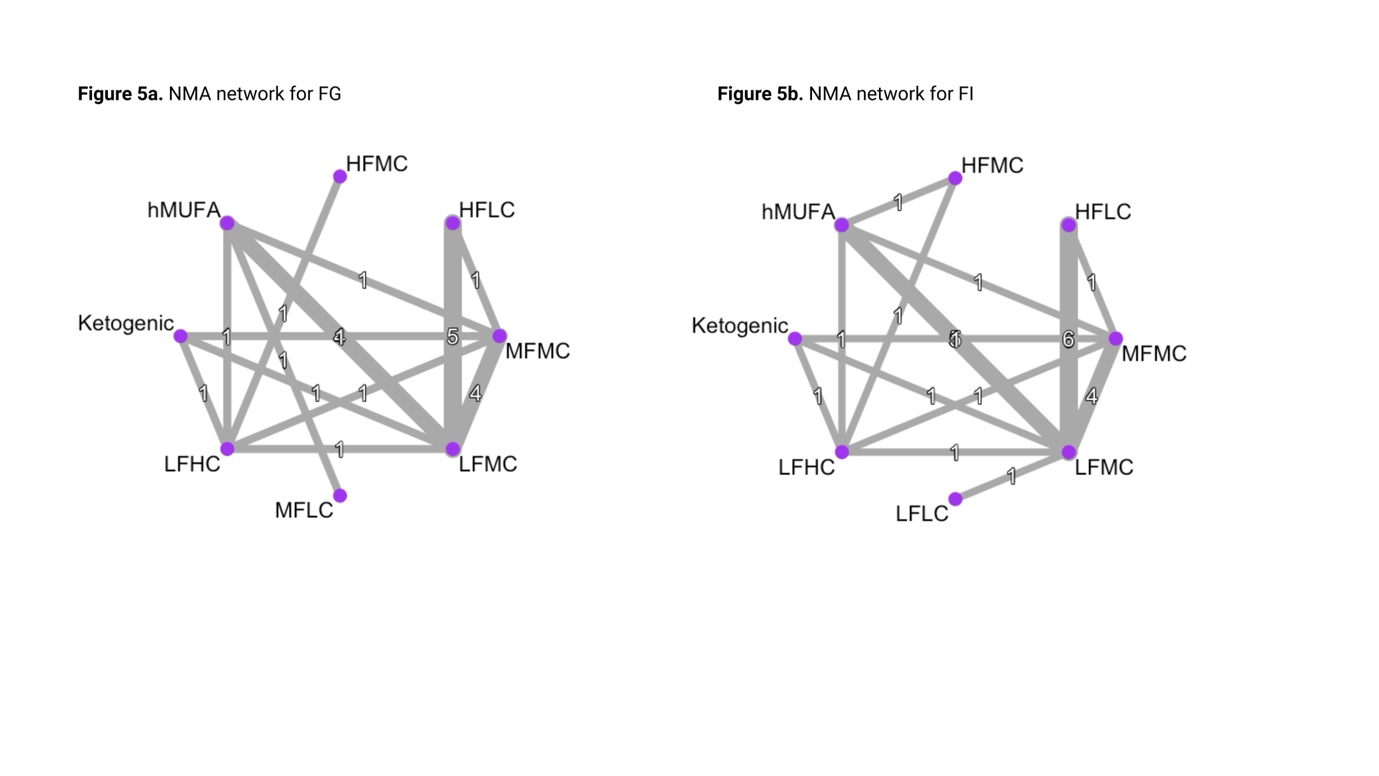
**

**Supplementary Figure 6.** Net heat plots assessing local inconsistency across dietary intervention networks.

**Legend:** Warmer colours indicate comparisons contributing more strongly to inconsistency, while cooler colours indicate negligible inconsistency. Grey cells represent comparisons not informed by direct evidence.

**a.** Net heat plot for body mass index


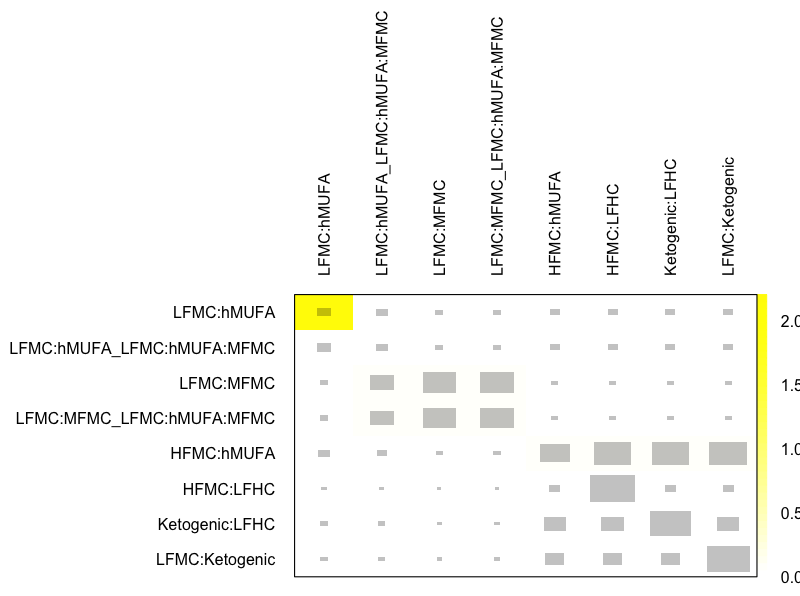


**b.** Net heat plot for waist circumference

**
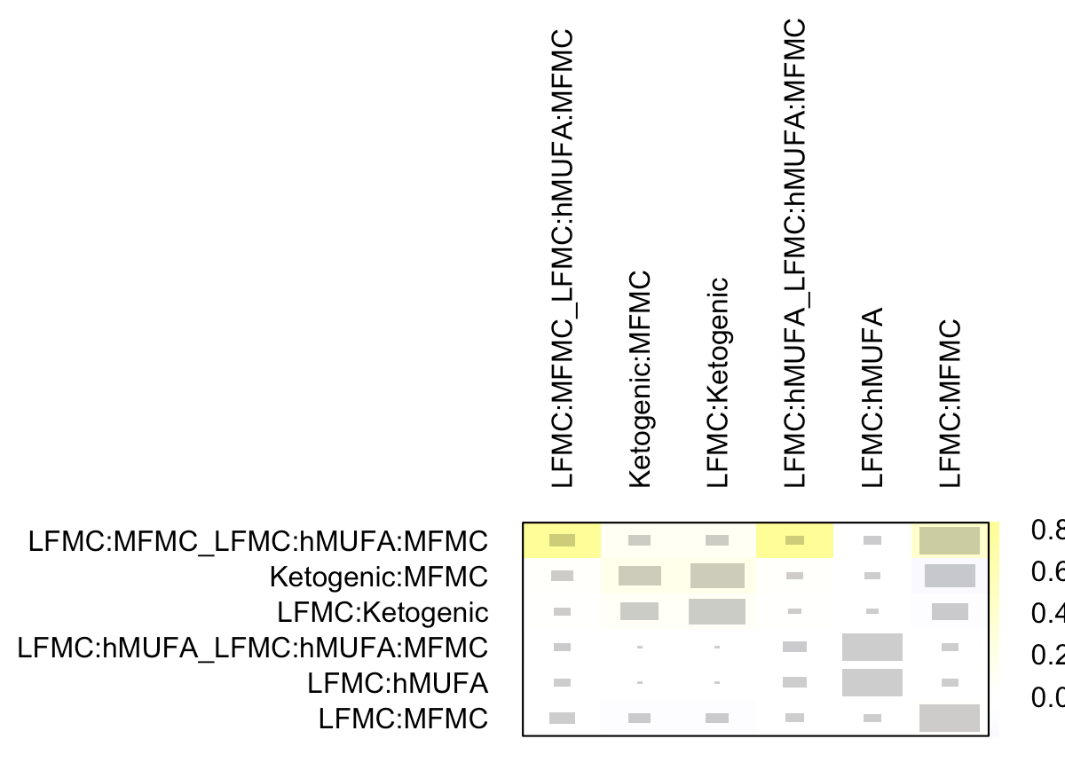
**

**c.** Net heat plot for low-density lipoprotein cholesterol


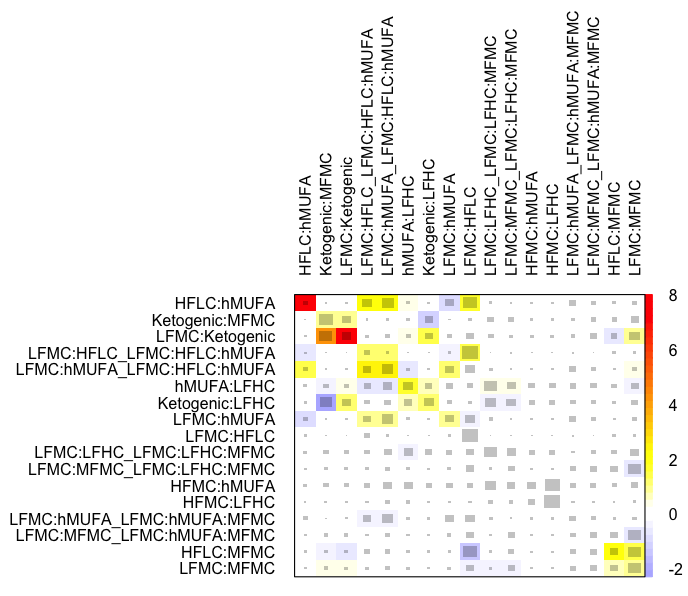


**d.** Net heat plot for high-density lipoprotein cholesterol


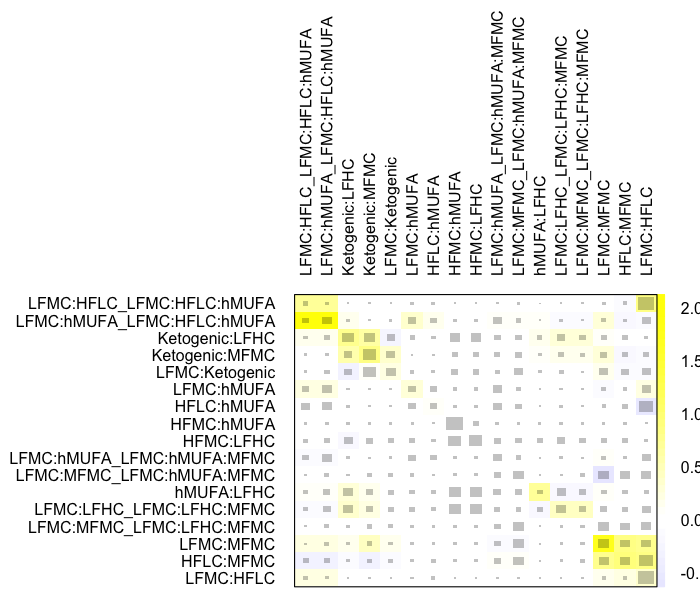


**e.** Net heat plot for total cholesterol


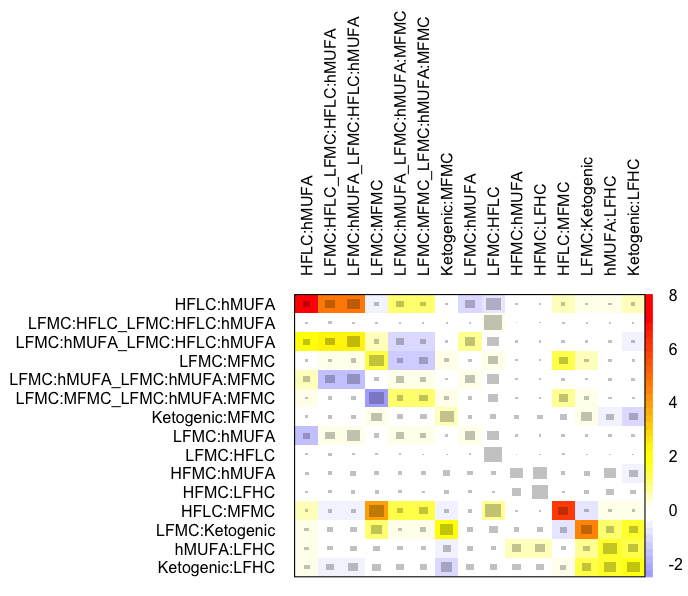


**f.** Net heat plot for triglycerides


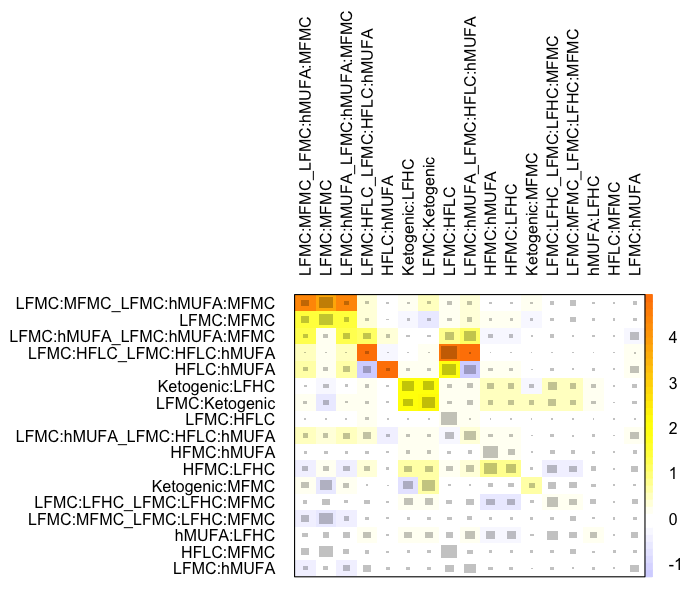


**g.** Net heat plot for systolic blood pressure


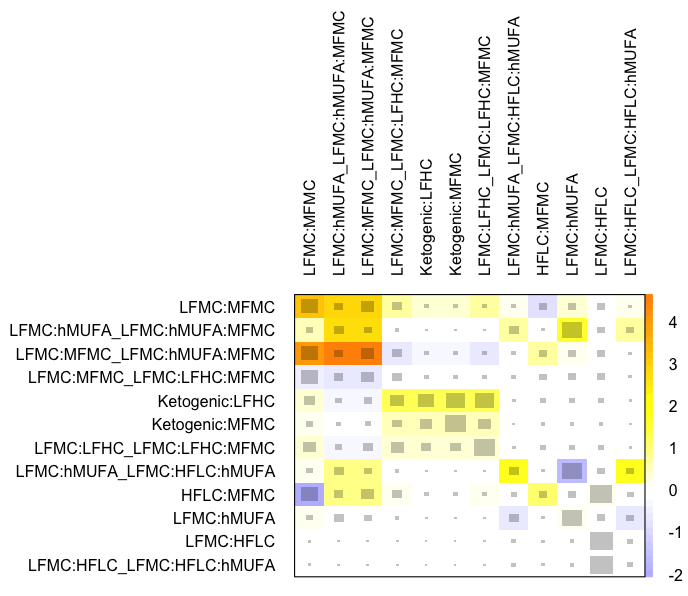


**h.** Net heat plot for diastolic blood pressure


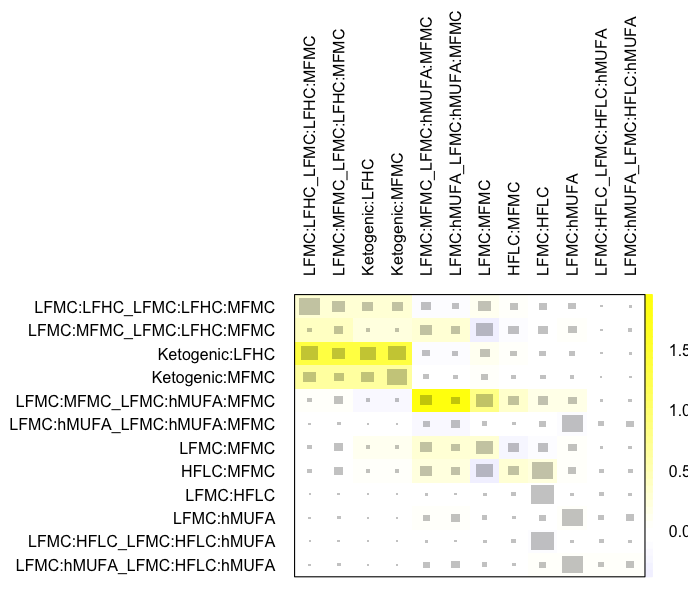


**i.** Net heat plot for fasting glucose


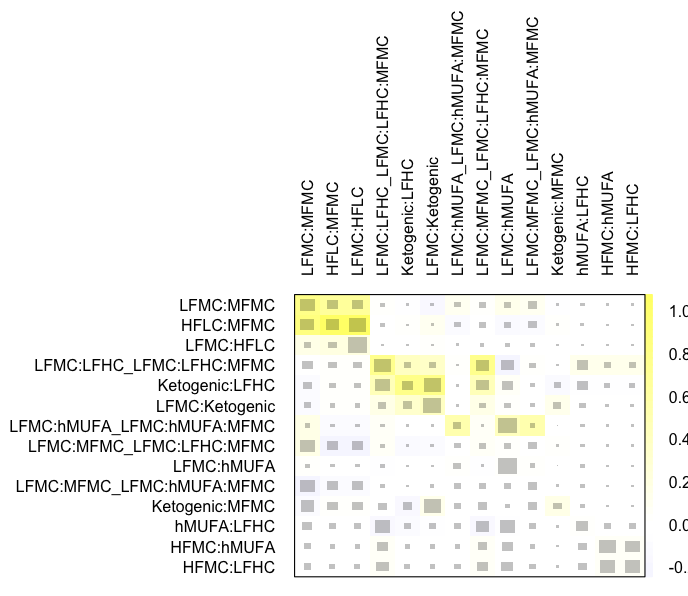


**j.** Net heat plot for fasting insulin


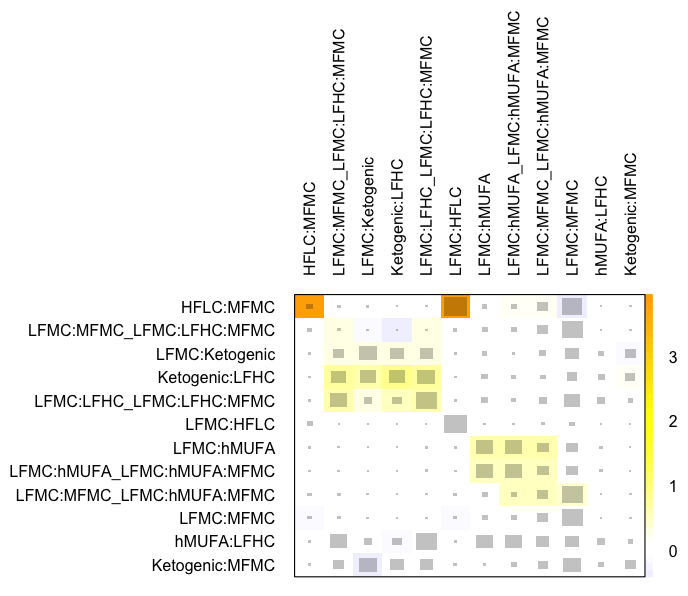


**Supplementary Figure 7.** Node-splitting analysis for assessing local inconsistency

**a.** Node-splitting results for body mass index


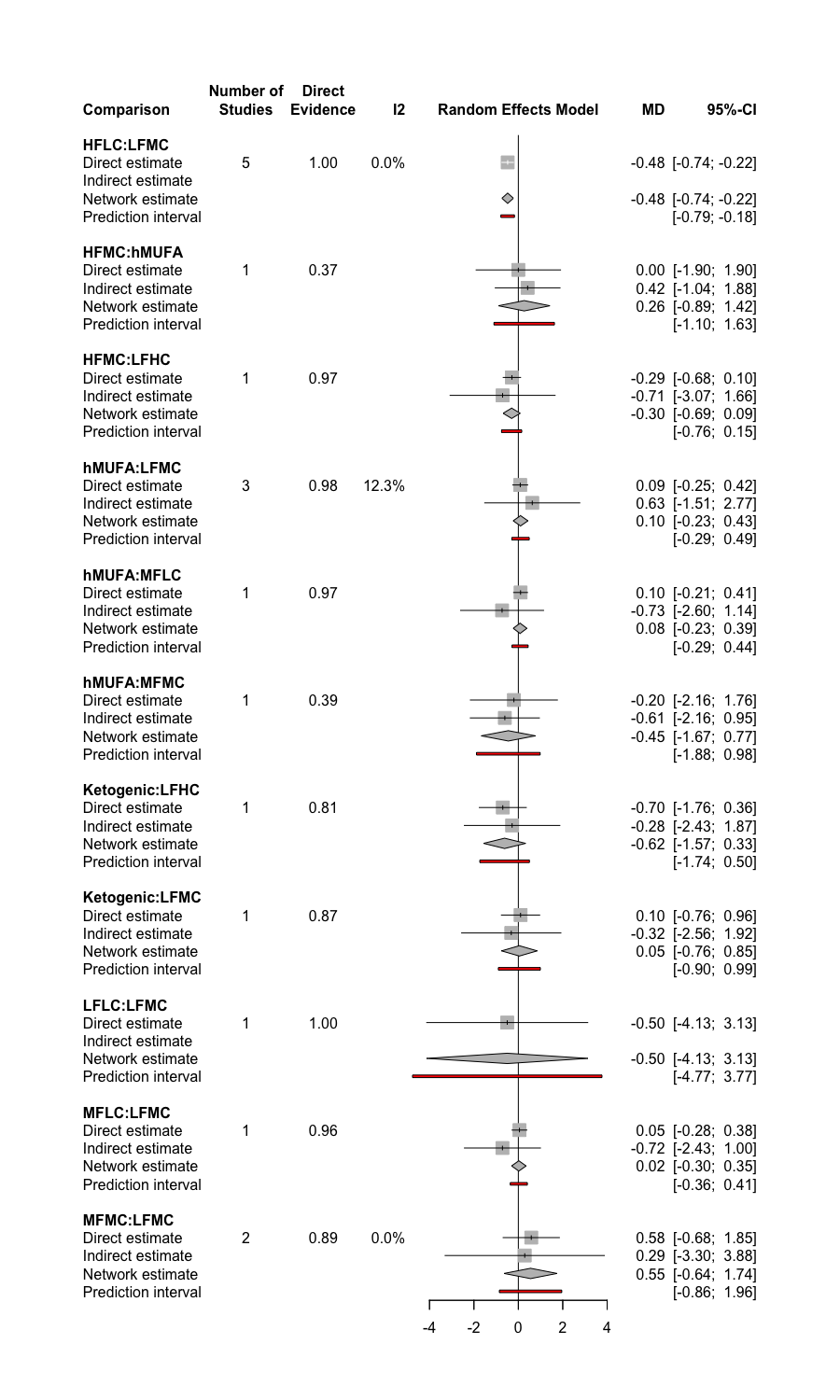


**b.** Node-splitting results for waist circumference


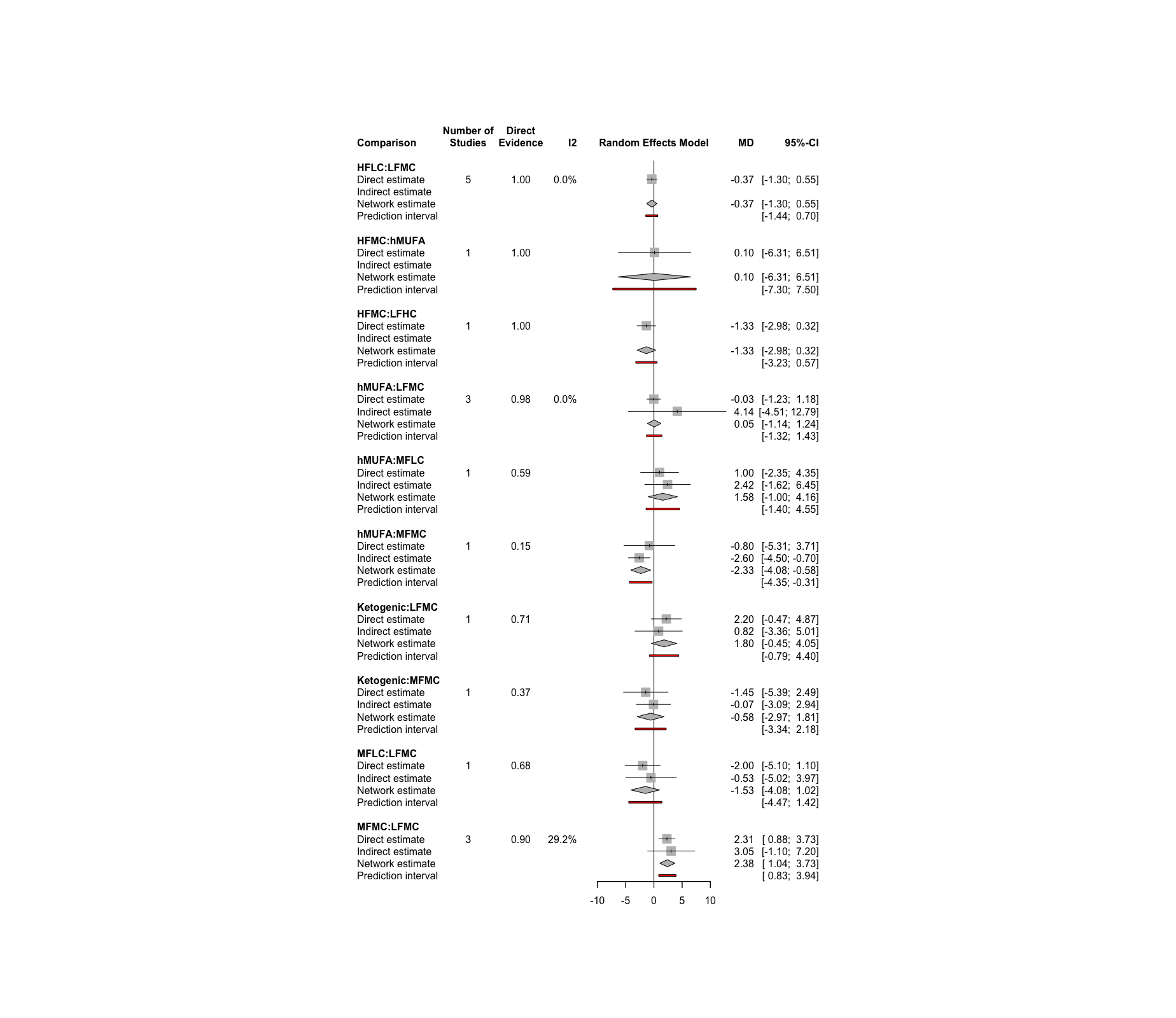


**c.** Node-splitting results for low-density lipoprotein cholesterol


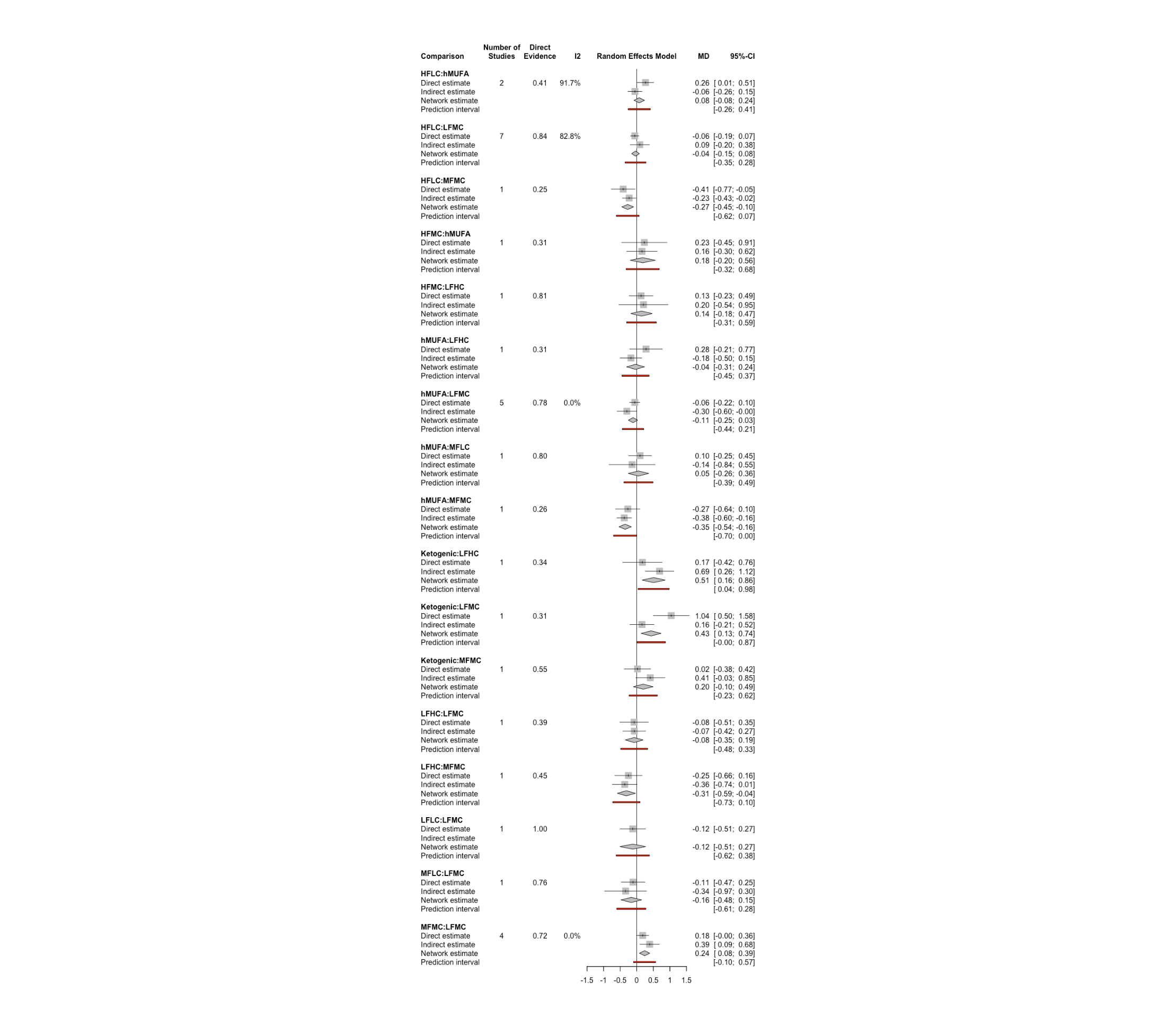


**d.** Node-splitting results for high-density lipoprotein cholesterol


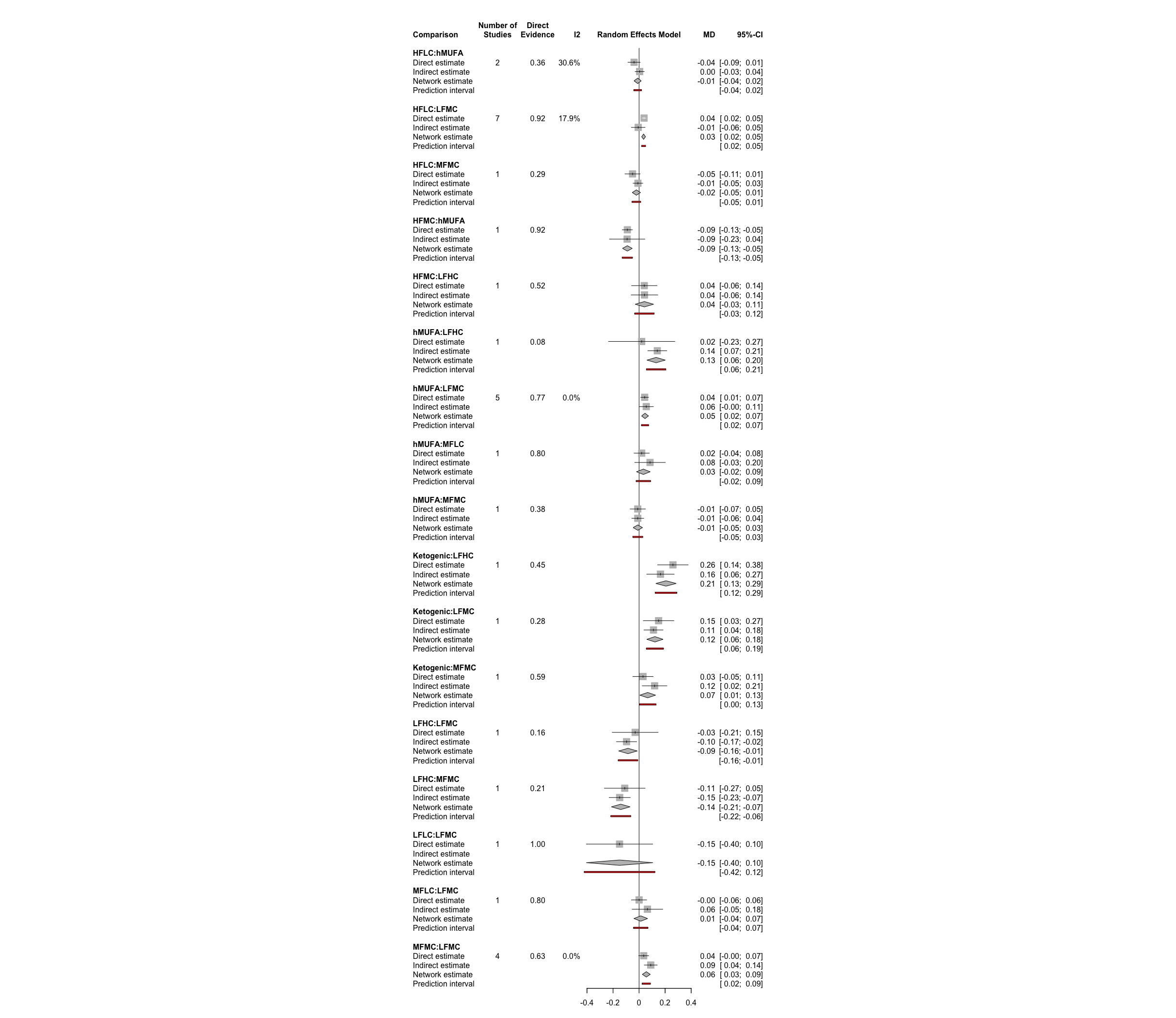


**e.** Node-splitting results for total cholesterol


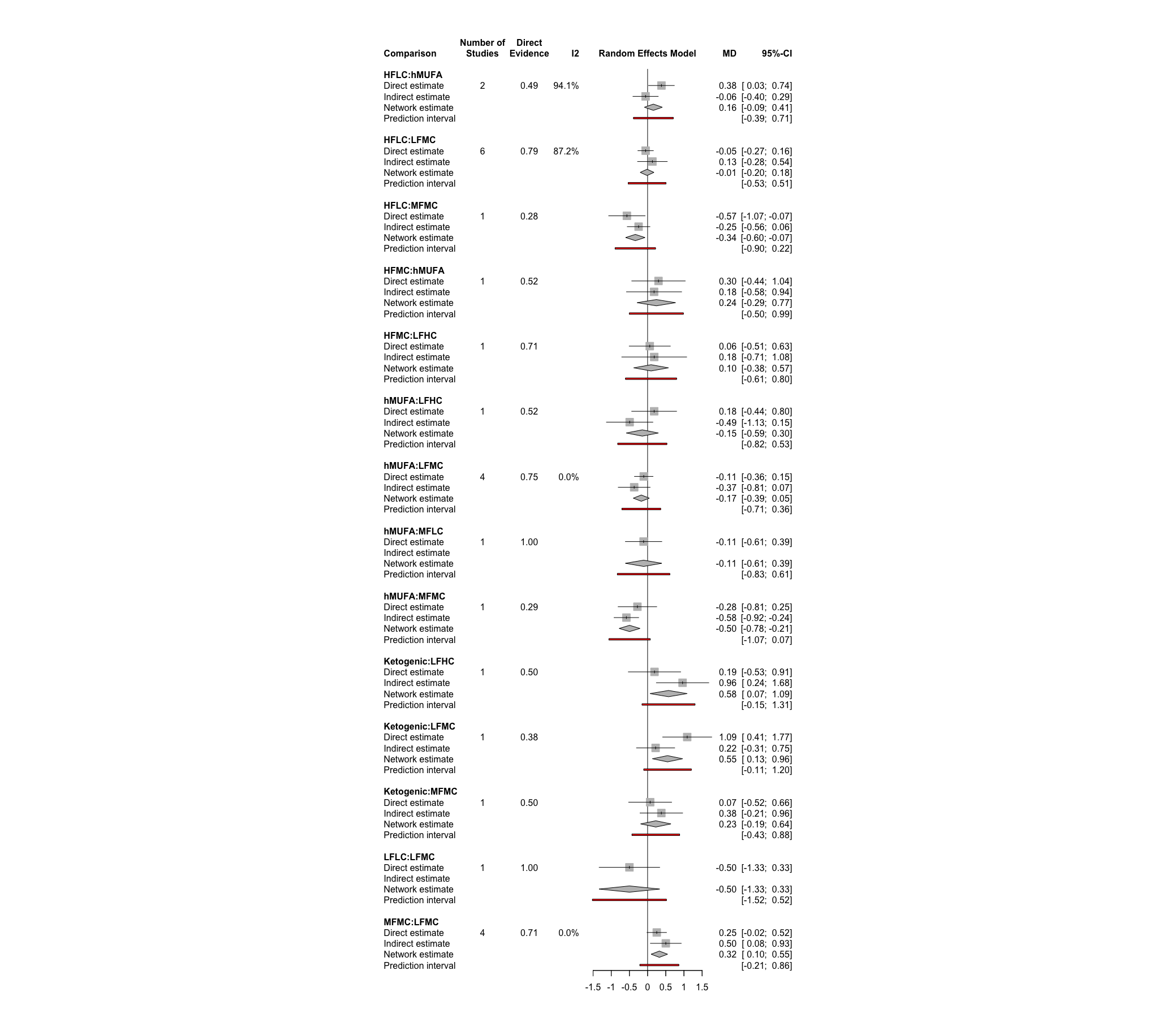


**f.** Node-splitting results for triglycerides


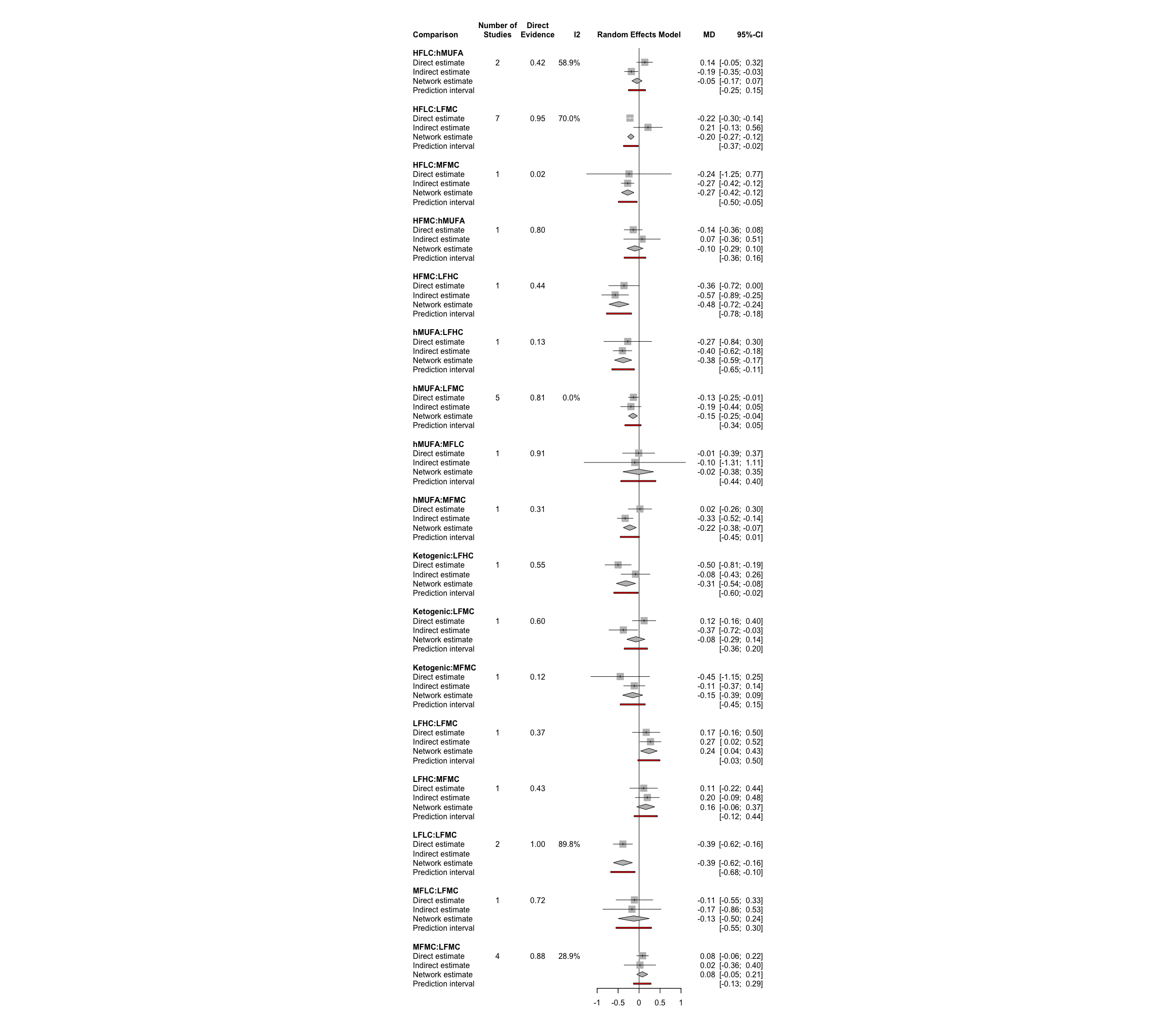


**g.** Node-splitting results for systolic blood pressure


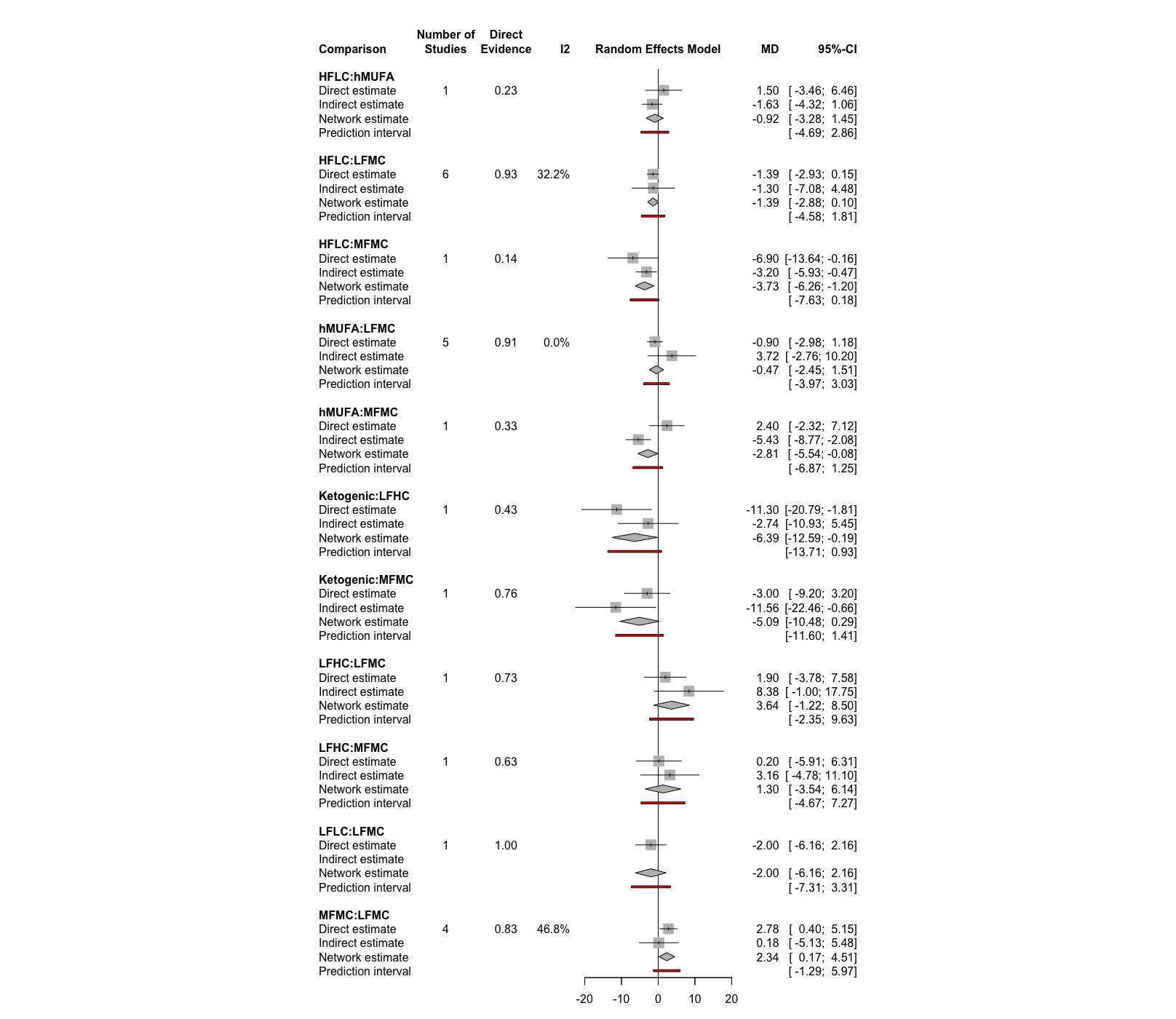


**h.** Node-splitting results for diastolic blood pressure


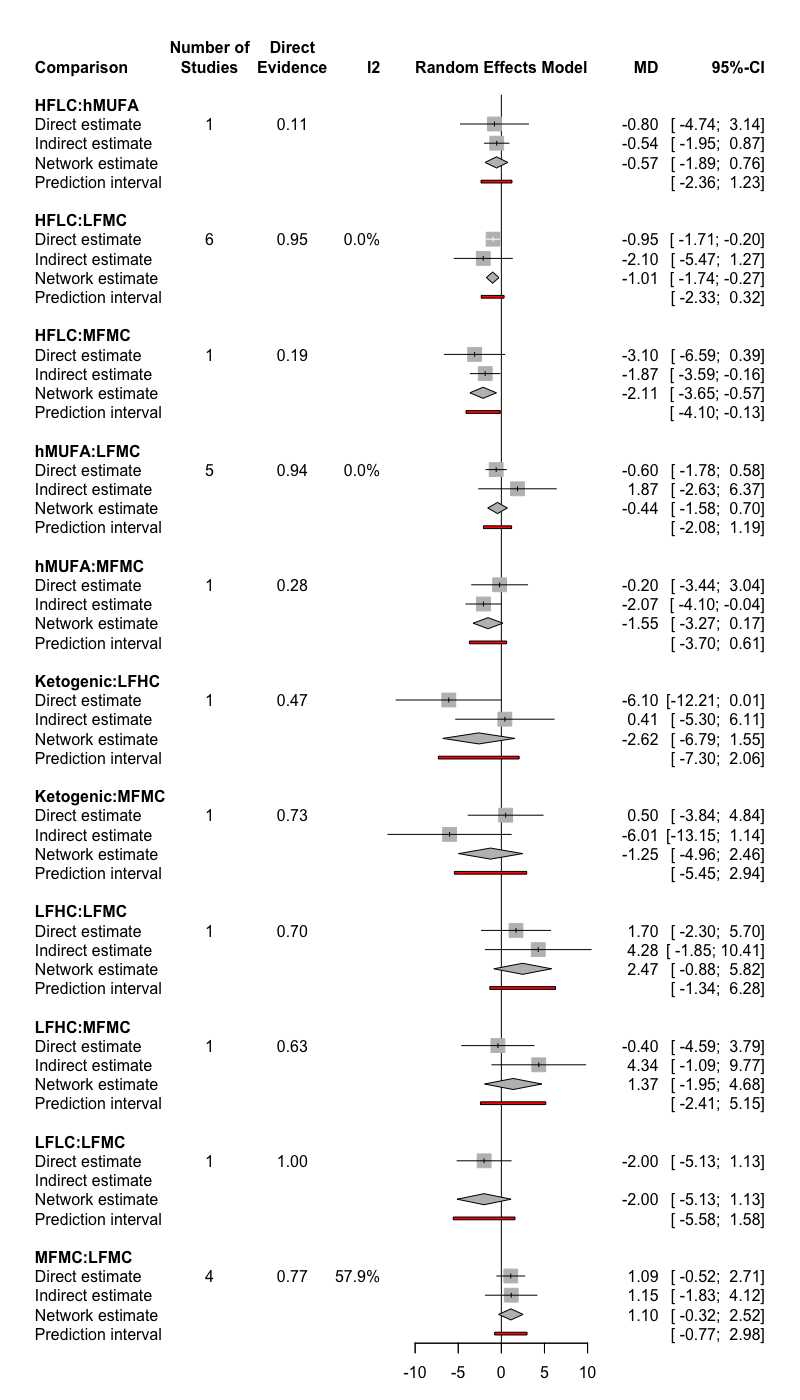


**i.** Node-splitting results for fasting glucose


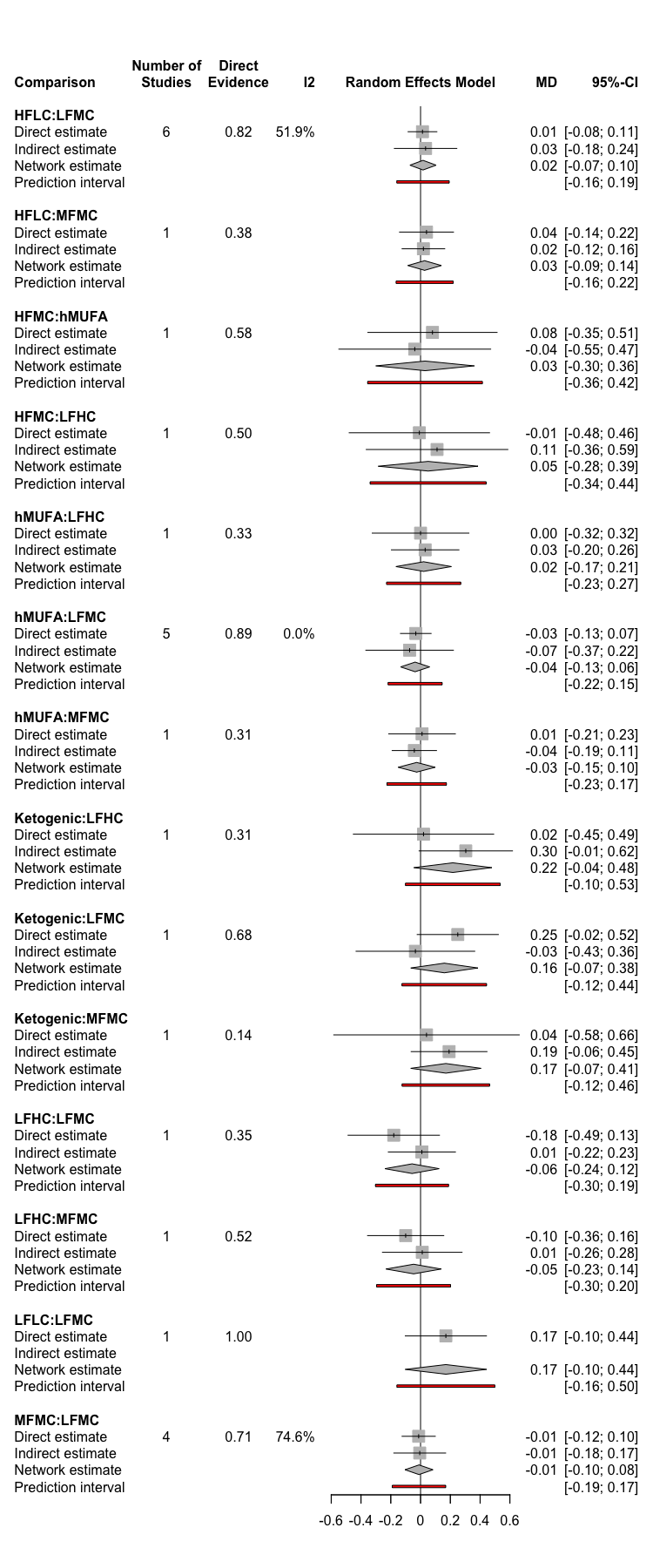


**j.** Node-splitting results for fasting insulin


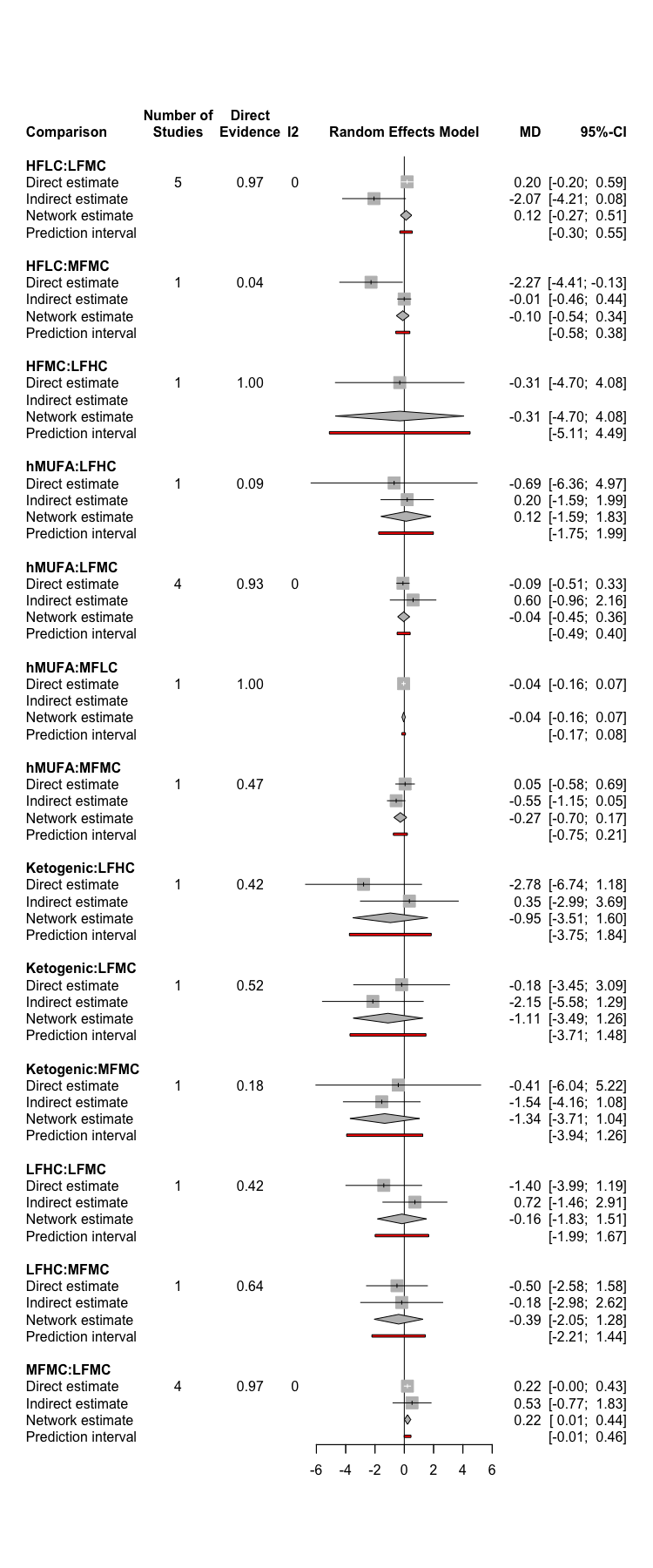


**Supplementary Figure 8.** Funnel plots for all outcomes

**a.** Funnel plot for body mass index


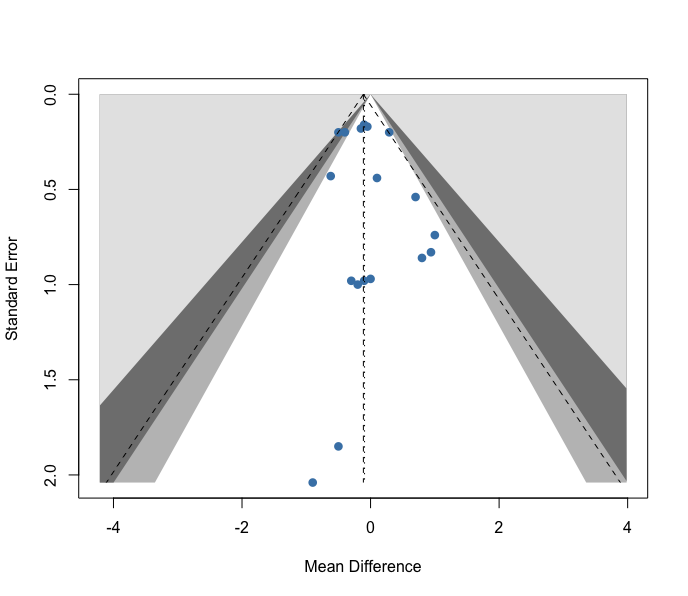


**b.** Funnel plot for waist circumference


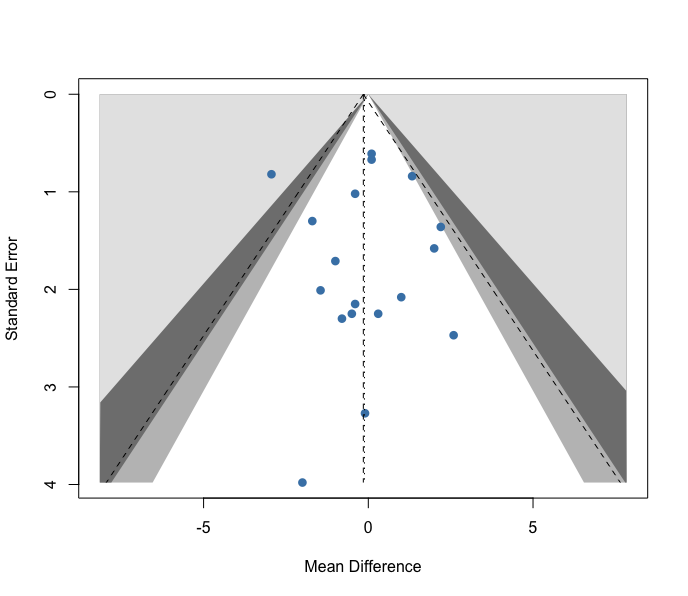


**c.** Funnel plot for low-density lipoprotein cholesterol

**d.** Funnel plot for high-density lipoprotein cholesterol


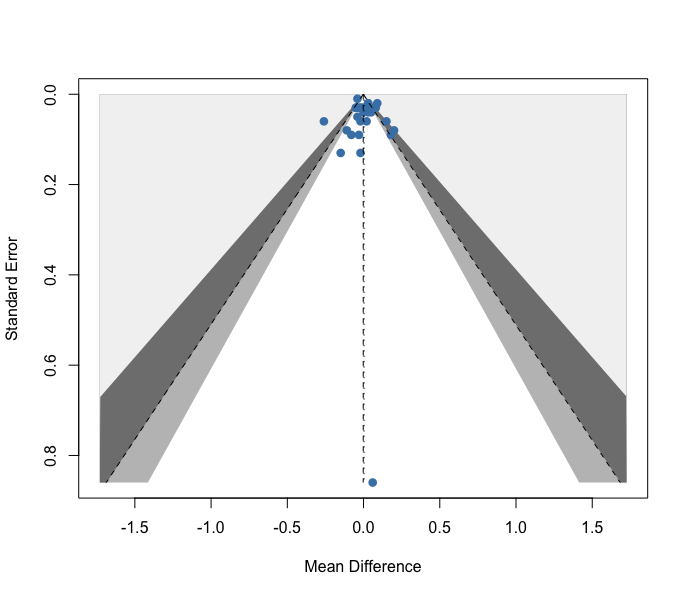


**e.** Funnel plot for total cholesterol


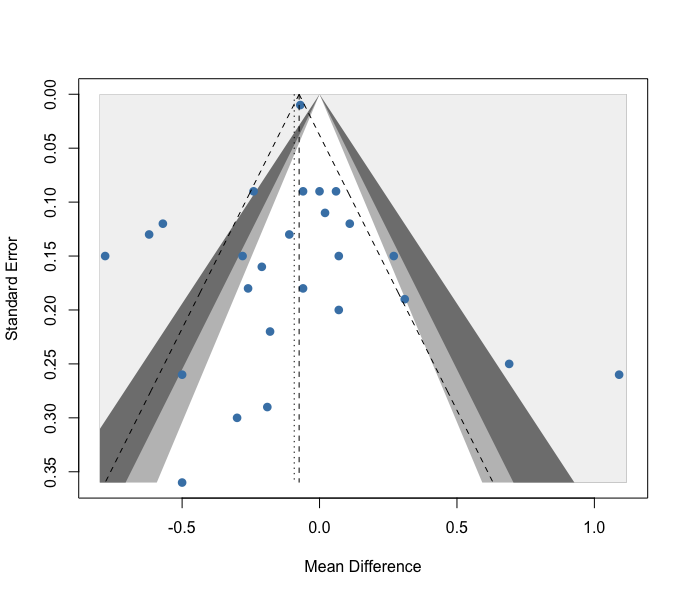


**f.** Funnel plot for triglycerides


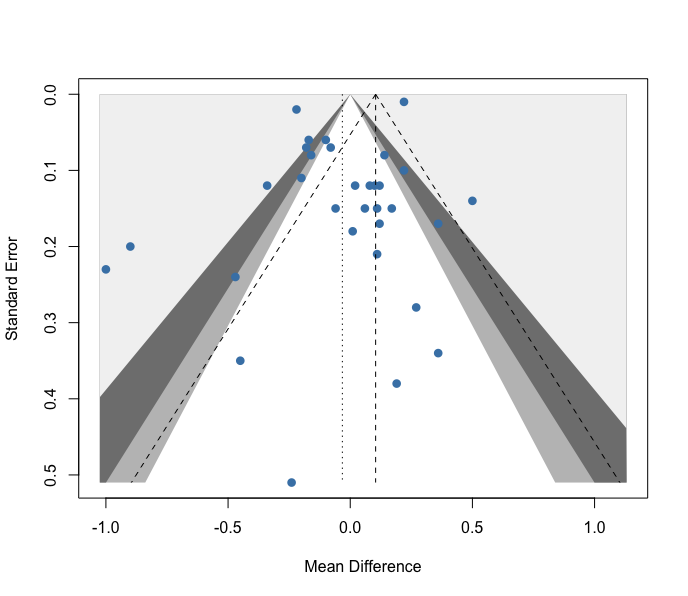


**g.** Funnel plot for systolic blood pressure


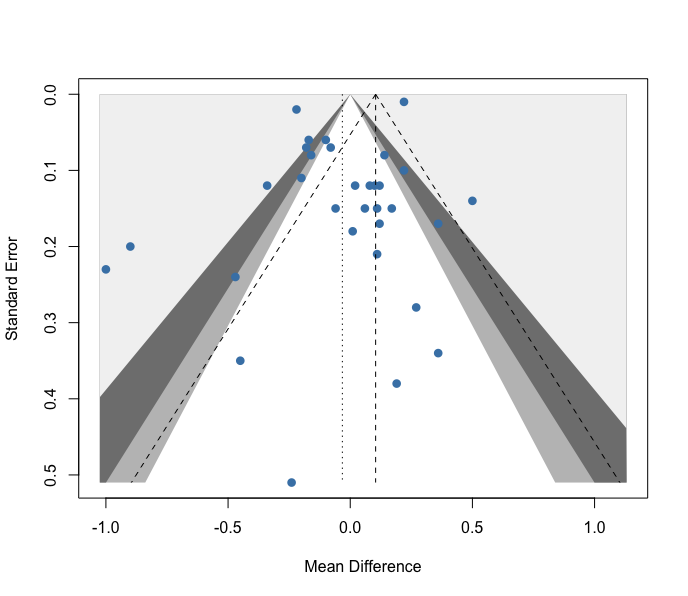


**h.** Funnel plot for diastolic blood pressure


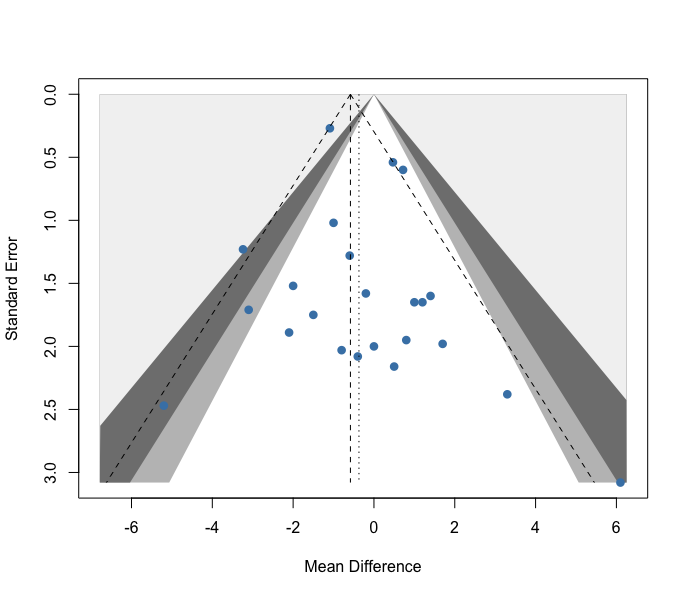


**i.** Funnel plot for fasting glucose


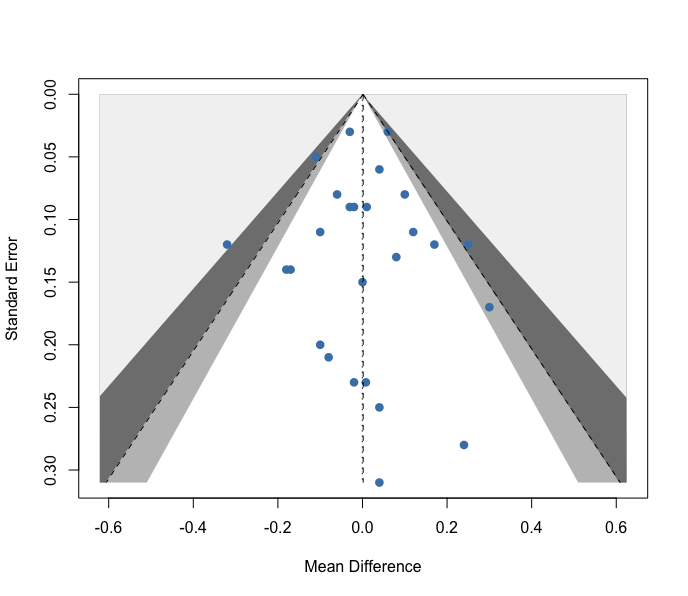


**j.** Funnel plot for fasting insulin


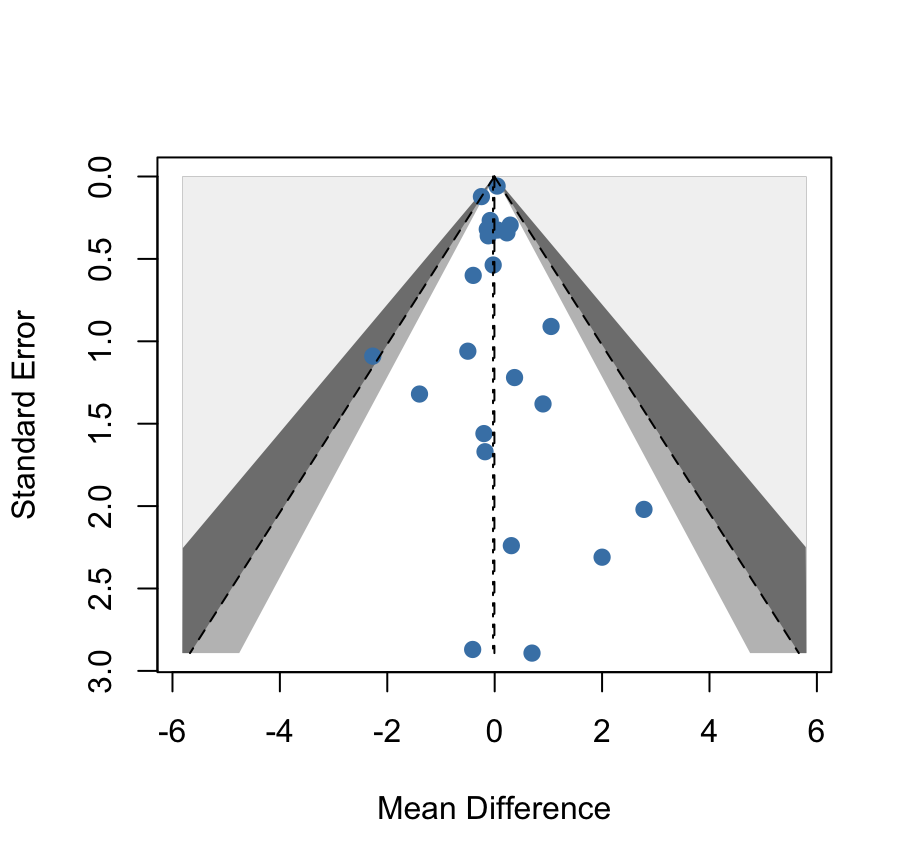


**Supplementary Figure 9.** Boxplots of baseline and study characteristics across dietary nodes to assess the transitivity assumption


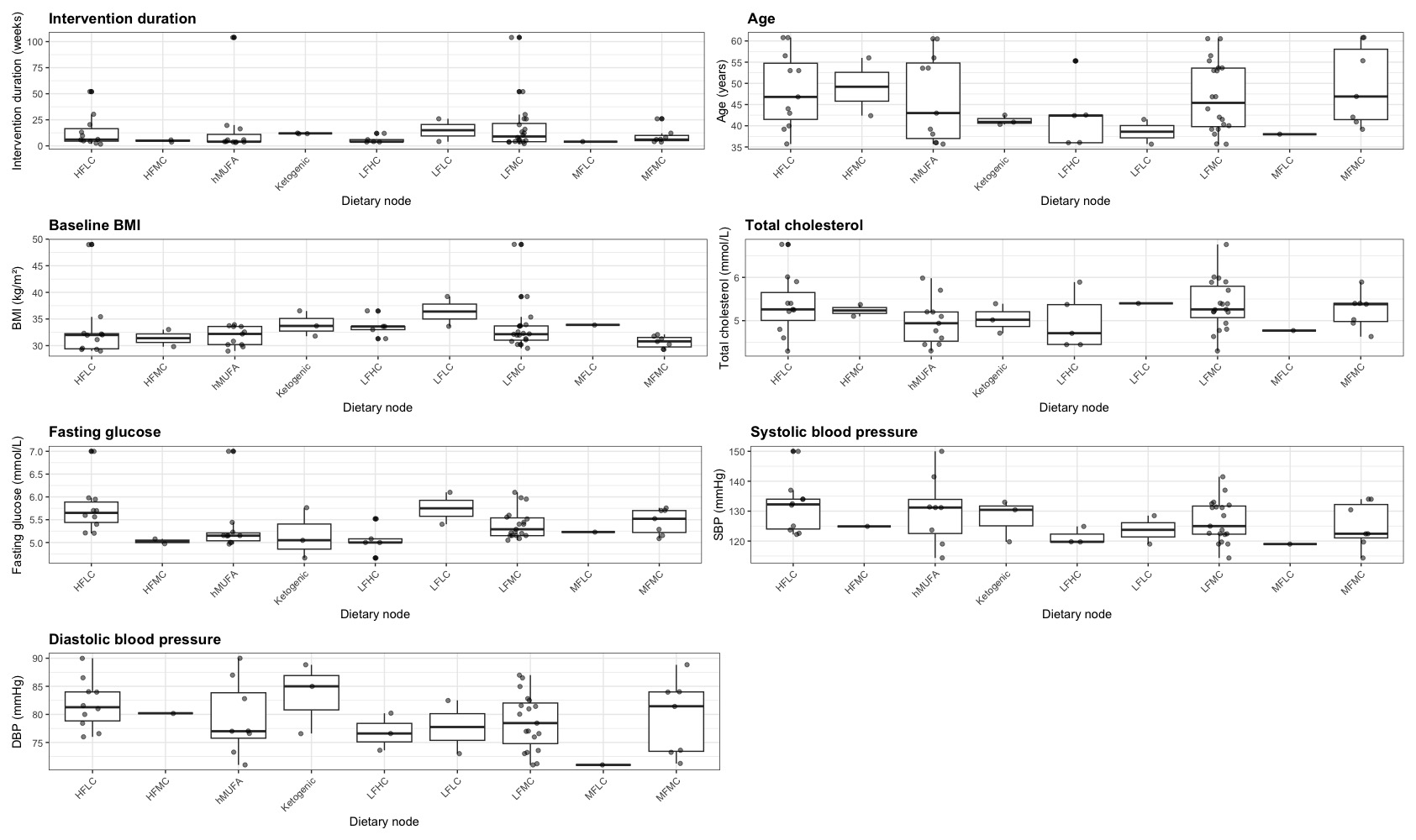


**Footnote:** The reference diet is LFMC (low-fat—moderate-carbohydrate). **Abbreviations:** HFLC, high-fat—low-carbohydrate; HFMC, high-fat—moderate-carbohydrate; hMUFA, high-monounsaturated fatty acid; LFHC, low-fat—high-carbohydrate; LFLC, low-fat—low-carbohydrate; LFMC, low-fat—moderate-carbohydrate; MFLC, moderate-fat—low-carbohydrate; MFMC, moderate-fat—moderate-carbohydrate; BMI, body mass index

**Supplementary Figure 10.** Effect of dietary interventions on body mass index (BMI) according to results from a sensitivity analysis restricting to energy-restricted (a) and non-energy-restricted (b) trials
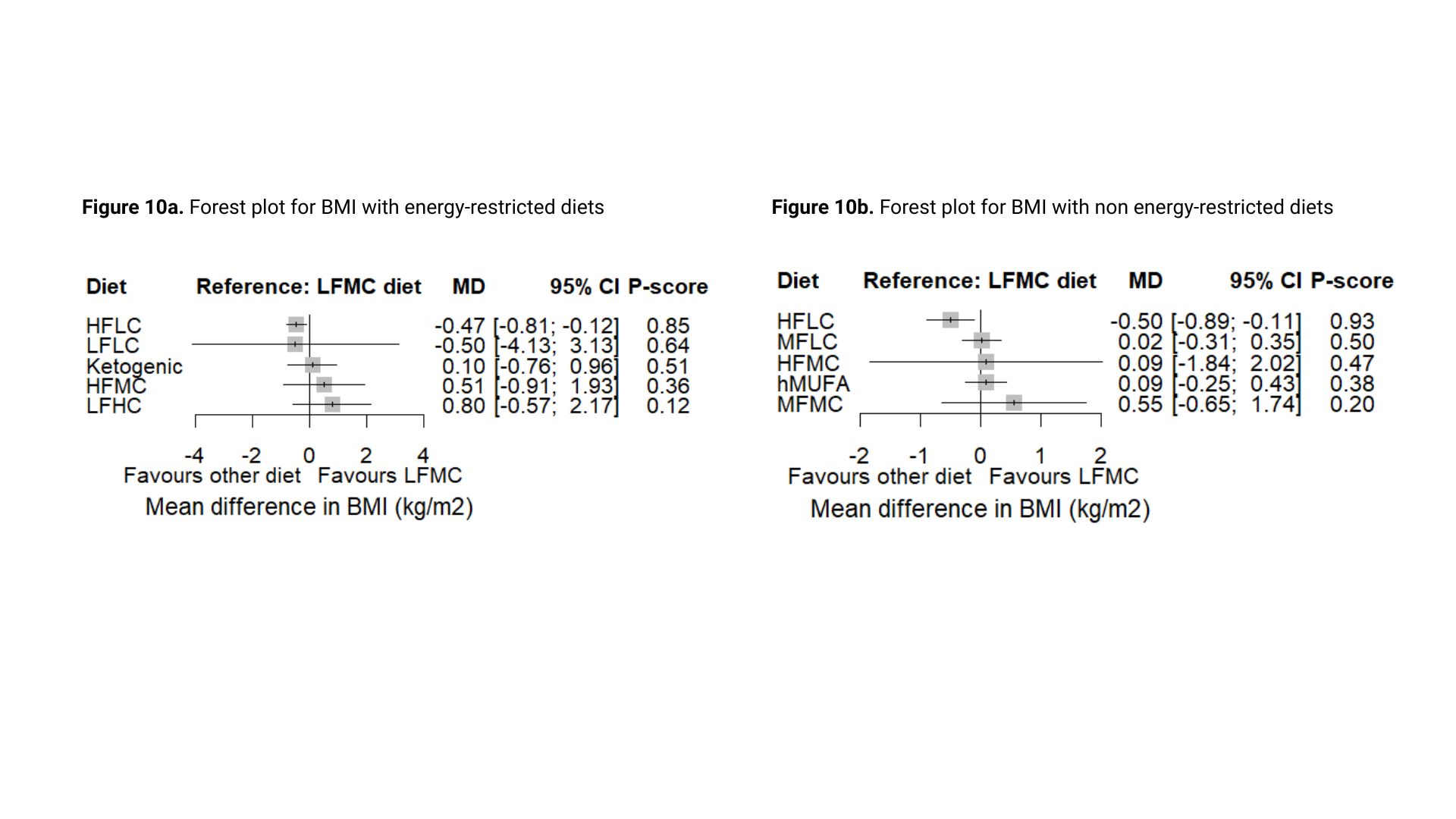


**Supplementary Figure 11.** Effect of dietary interventions on waist circumference (WC) according to results from a sensitivity analysis restricting to energy-restricted (a) and non-energy-restricted (b) trials


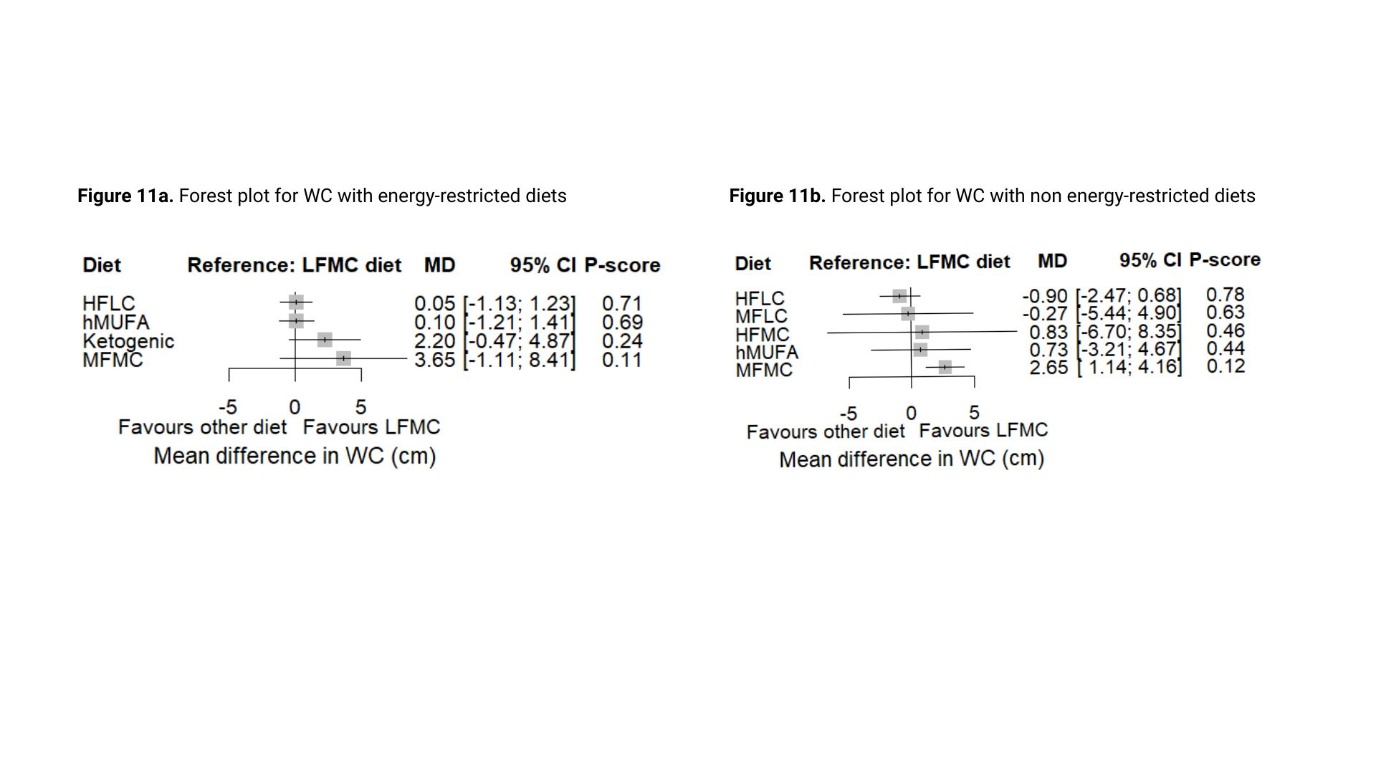


**Supplementary Figure 12.** Effect of dietary interventions on low-density lipoprotein cholesterol (LDL-C) according to results from a sensitivity analysis restricting to energy-restricted (a) and non-energy-restricted (b) trials


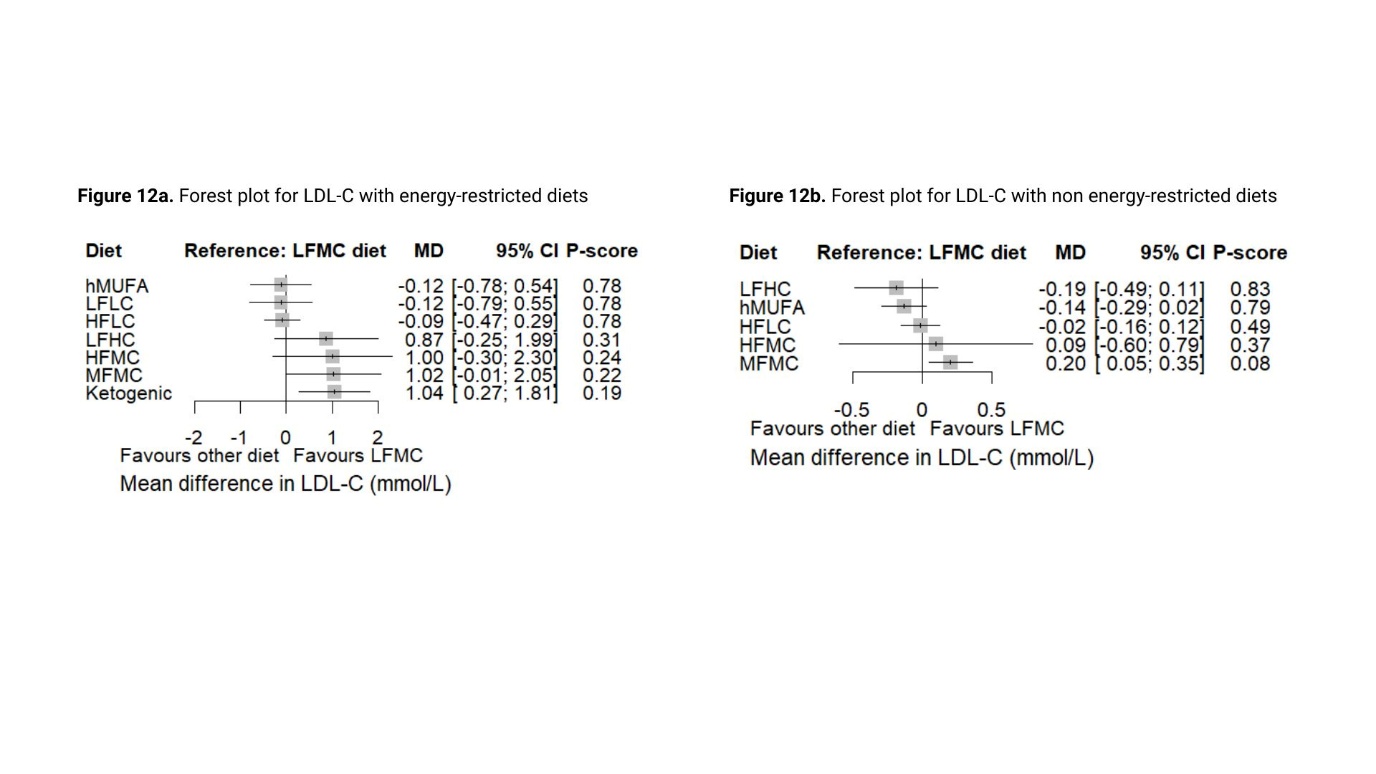


**Supplementary Figure 13.** Effect of dietary interventions on high-density lipoprotein cholesterol (HDL-C) according to results from a sensitivity analysis restricting to energy-restricted (a) and non-energy-restricted (b) trials


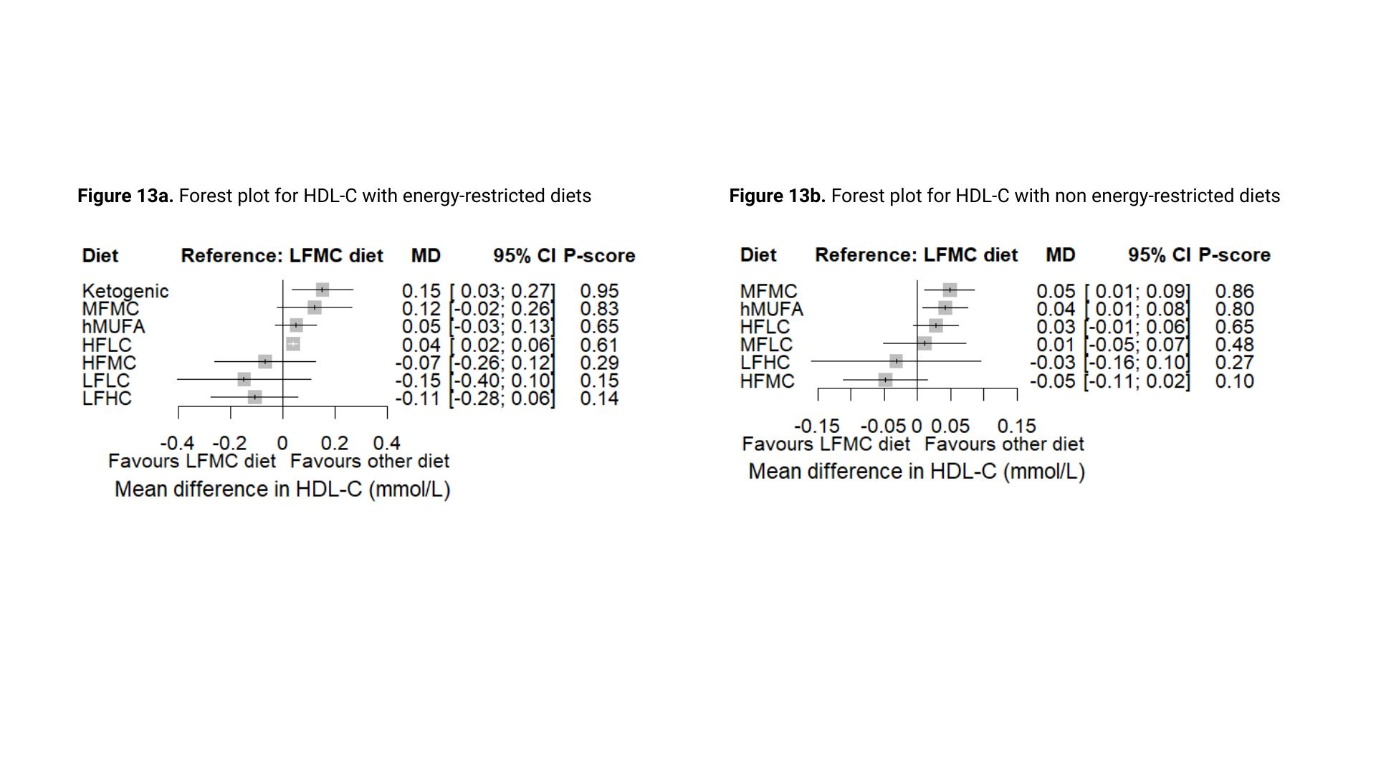


**Supplementary Figure 14.** Effect of dietary interventions on total cholesterol (TC) according to results from a sensitivity analysis restricting to energy-restricted (a) and non-energy-restricted (b) trials
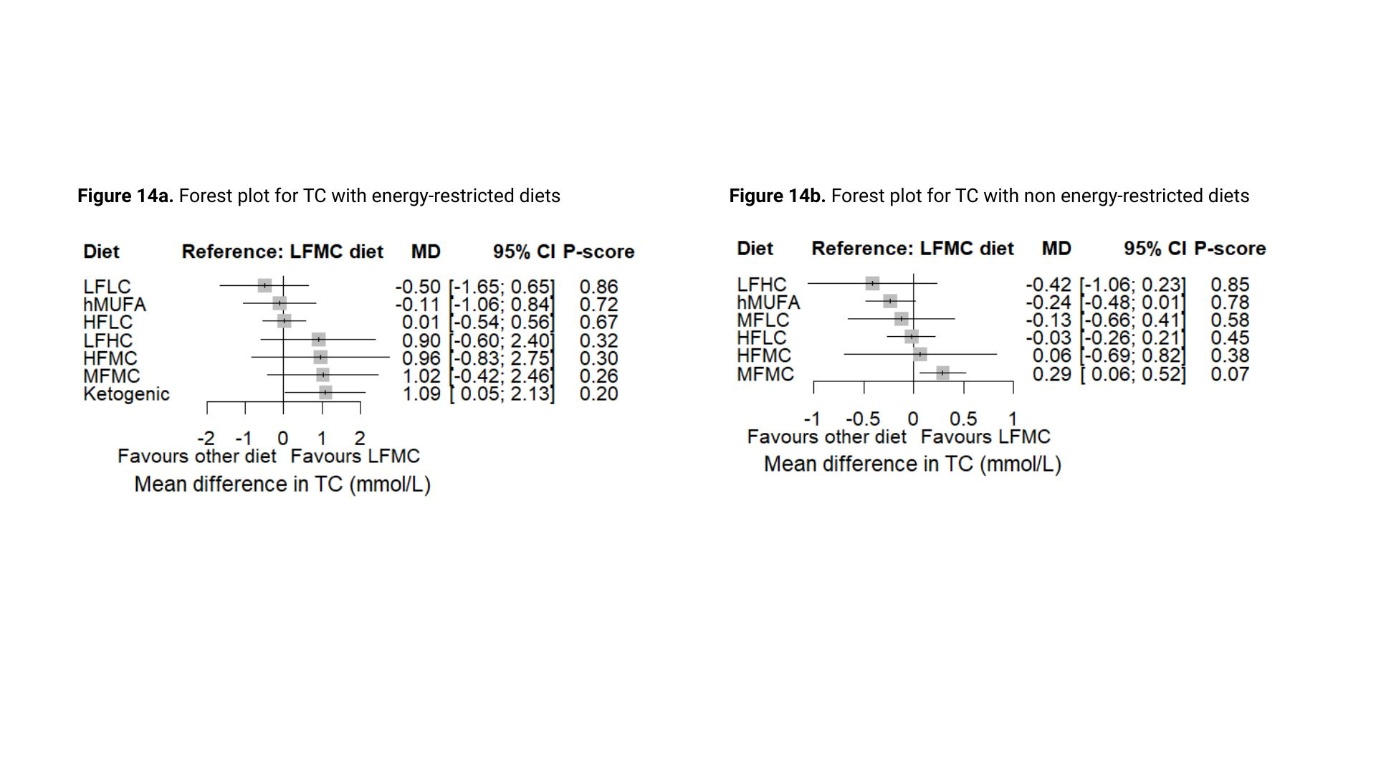


**Supplementary Figure 15.** Effect of dietary interventions on triglycerides (TG) according to results from a sensitivity analysis restricting to energy-restricted (a) and non-energy-restricted (b) trials


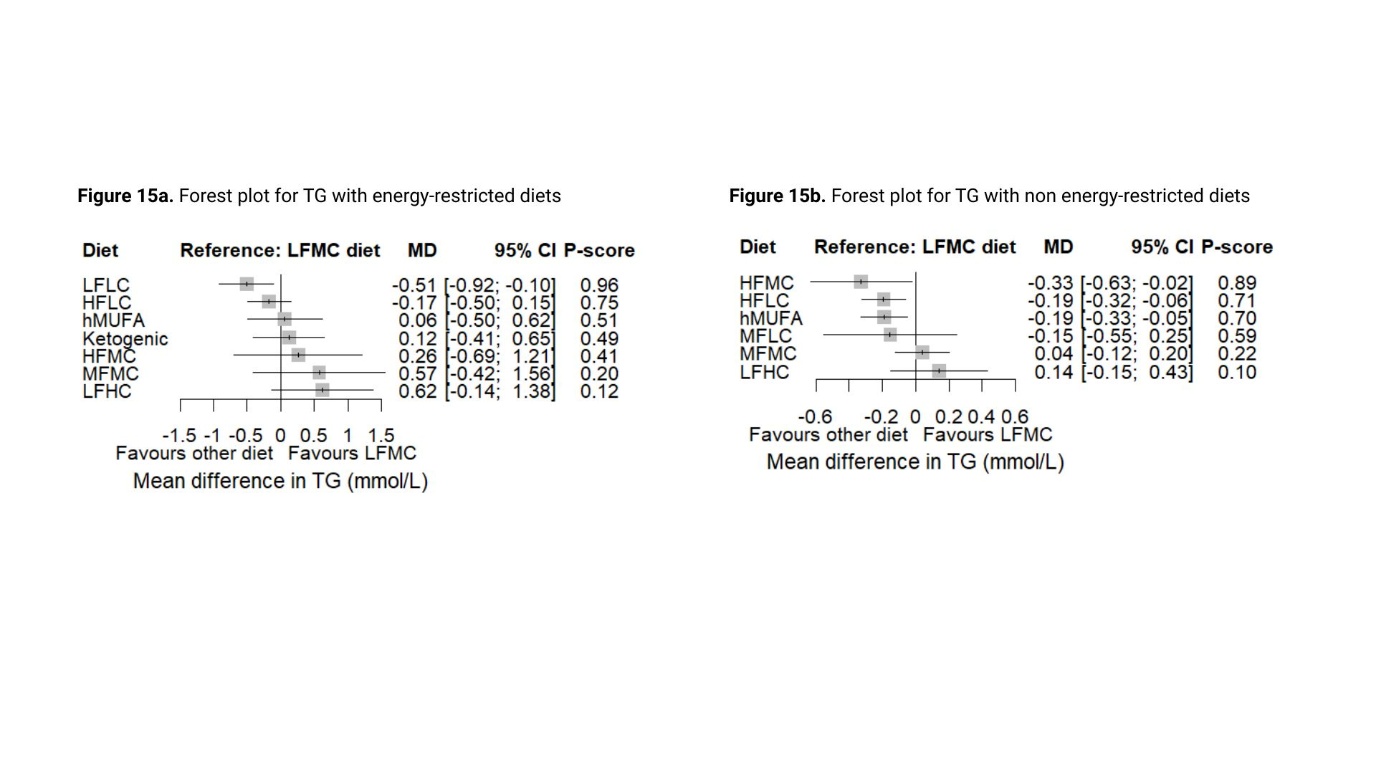


**Supplementary Figure 16.** Effect of dietary interventions on systolic blood presure (SBP) according to results from a sensitivity analysis restricting to energy-restricted (a) and non-energy-restricted (b) trials


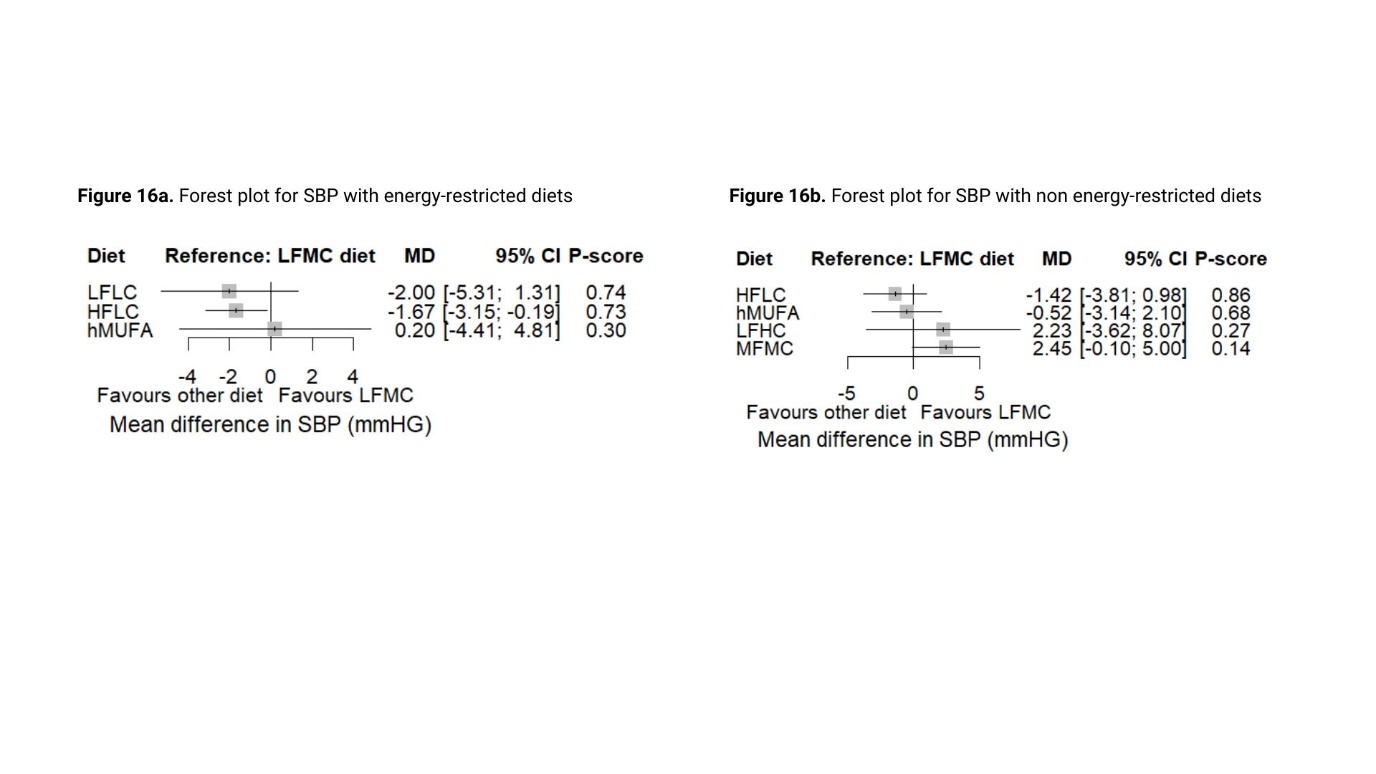


**Supplementary Figure 17.** Effect of dietary interventions on diastolic blood presure (DBP) according to results from a sensitivity analysis restricting to energy-restricted (a) and non-energy-restricted (b) trials


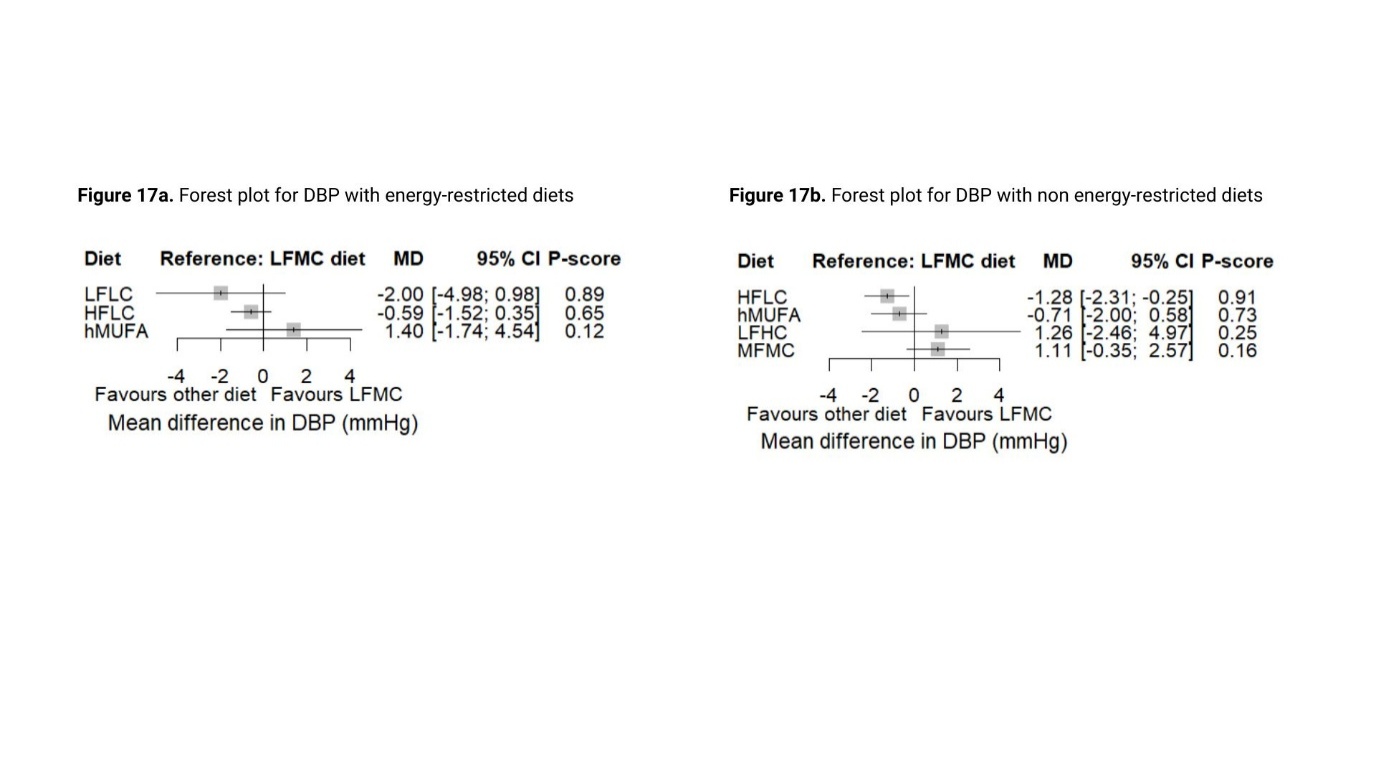


**Supplementary Figure 18.** Effect of dietary interventions on fasting glucose (FG) according to results from a sensitivity analysis restricting to energy-restricted (a) and non-energy-restricted (b) trials


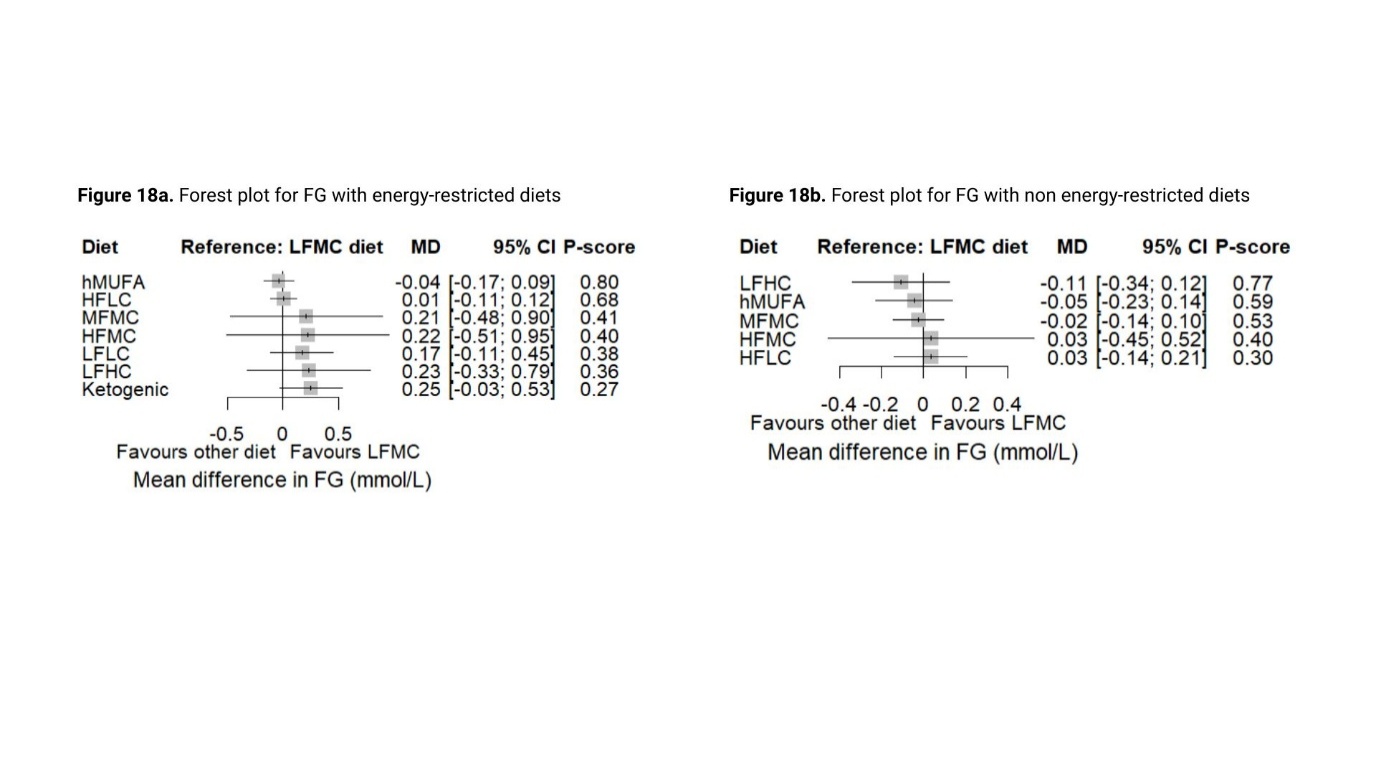


**Supplementary Figure 19.** Effect of dietary interventions on fasting insulin (FI) according to results from a sensitivity analysis restricting to energy-restricted (a) and non-energy-restricted (b) trials


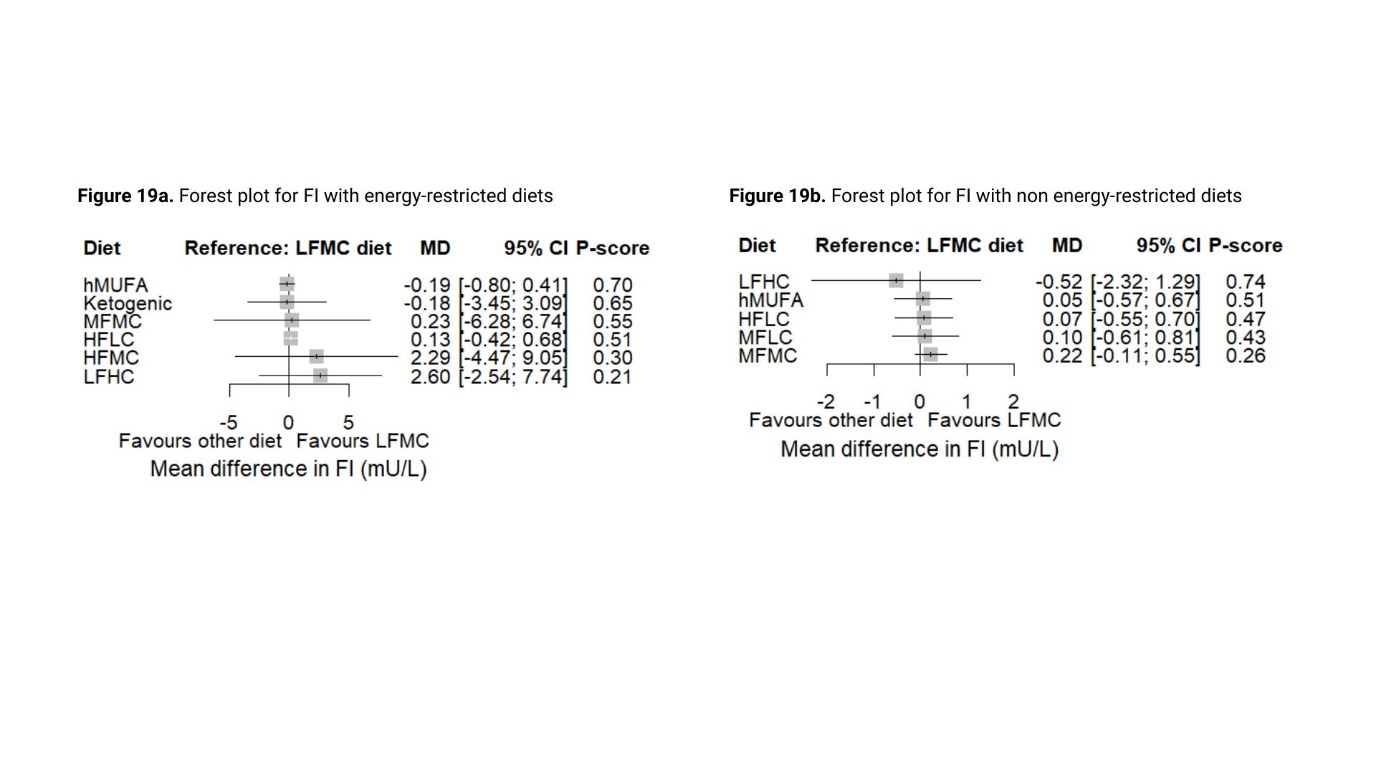


**Supplementary Table 6.** Distribution of potential effect modifiers across dietary intervention nodes to assess the transitivity assumption

| **Study** | **Medication** | **Smoking** | **Excersise** | **Proportion of female** | **Energy-restriction** | **Type of feeding** | **Interv. duration (weeks)** | **Age (years)** | **BMI (kg/m^2^)** | **TC (mmol/L)** | **FG (mmol/L)** | **SBP (mmHg)** | **DBP (mmHg)** |
| --- | --- | --- | --- | --- | --- | --- | --- | --- | --- | --- | --- | --- | --- |
| **HFLC** | | | | | | | | | | | | | |
| Bazzano et al., 2014 | Yes | Yes | Usual | 88% | No | Self-prepared | 52 | 35.4 (4.1) | 5.21 (1.1) | 5.21 (0.55) | 122.6 (13.4) | 78.45 (8.66) | 35.4 (4.1) |
| Boers et al., 2014 | Yes | No | Usual | 73% | No | Provided | 2 | 32 (5.57) | 5.9 (1.2) | 5.95 (0.76) | 132 (14.8) | 86.5 (10.99) | 32 (5.57) |
| Chiu et al., 2017 | No | No | Usual | 17% | No | Half-provided | 3 | 29 (2.72) | 4.6 (0.91) | <7.0 | <150 | <90 | 29 (2.72) |
| Ebbeling et al., 2021 | No | No | Usual | 69% | No | Provided | 20 | 32.2 (4.8) | 4.3 (0.89) | NR | 123.7 (10.7) | 76.6 (7.4) | 32.2 (4.8) |
| Fechner et al., 2020 (a) | No | No | Usual | 53% | No | Provided | 6 | 29.3 (2.63) | 5.4 (0.84) | 5.7 (0.59) | 134 (13.1) | 84 (8.3) | 29.3 (2.63) |
| Fechner et al., 2020 (b) | No | No | Usual | 53% | No | Provided | 6 | 29.3 (2.63) | 5.4 (0.84) | 5.7 (0.59) | 134 (13.1) | 84 (8.3) | 29.3 (2.63) |
| Guo et al., 2022 | NR | Yes | Moderate | 73% | Yes | Self-prepared | 10 | 29.5 (1.86) | 5.26 (0.22) | 5.98 (0.3) | NR | NR | 29.5 (1.86) |
| Jenkins et al., 2014 | Yes | No | Usual | 62% | Yes | Self-prepared | 30 | 31.1 (1.25) | 6.76 (0.51) | 5.2 (0.22) | 125 (6.75) | 76 (3.55) | 31.1 (1.25) |
| Mousavi et al., 2023 | No | No | Usual | 100% | Yes | Self-prepared | 13 | 32.3 (3.61) | 5.25 (0.92) | 5.56 (0.63) | 122.2 (10.8) | 81.55 (7.9) | 32.3 (3.61) |
| Sacks et al., 2014 | No | Yes | Usual | 52% | No | Provided | 5 | 32 (6) | 6.01 (1.09) | 5.8 (0.95) | 132 (9.1) | 80 (7.5) | 32 (6) |
| Tricò et al., 2021 | NR | NR | Usual | 68% | Yes | Self-prepared | 4 | 49 (7.18) | 4.8 (0.95) | 5.6 (0.6) | 137 (13.2) | 81(6.54) | 49 (7.18) |
| **HFMC** | | | | | | | | | | | | | |
| Anderson-Vasquez et al., 2015 | No | NR | Usual | 100% | No | Self-prepared | 4 | 29.8 (3.1) | 5.1 (0.42) | 4.97 (0.34) | NR | NR | 29.8 (3.1) |
| Rajaie et al., 2014 | Yes | No | Usual | 100% | Yes | Self-prepared | 6 | 33 (5) | 5.37 (0.89) | 5.08 (1.71) | 124.9 (13.8) | 80.2 (8.7) | 33 (5) |
| **Ketogenic** | | | | | | | | | | | | | |
| Pinsawas et al., 2024 | Yes | No | Usual | 72% | Yes | Self-prepared | 12 | 40.9 (7.9) | 31.8 (0.87) | 5.02 (0.99) | 5.76 (0.35) | 130 (12.4) | 88.8 (9.3) |
| Ruth et al., 2014 | No | No | Usual | 89% | Yes | Self-prepared | 12 | 42.5 (12.1) | 36.5 (4.7) | 4.71 (0.78) | 4.66 (0.9) | 120 (14.7) | 76.6 (9.35) |
| Veum et al., 2017 | No | NR | Usual | 0% | Yes | Self-prepared | 12 | 40.3 (5.2) | 33.7 (3.03) | 5.39 (1.14) | 5.05 (0.44) | 133 (14.54) | 85 (8.62) |
| **LFHC** | | | | | | | | | | | | | |
| Marina et al., 2014 | No | No | Usual | 30% | No | Provided | 4 | 36 (2.9) | 33.6 (1.3) | 4.45 (0.36) | 5 (0.1) | NR | NR |
| Rajaie et al., 2014 | Yes | No | Usual | 100% | Yes | Self-prepared | 6 | 42.4 (6.4) | 33 (5) | 5.37 (0.89) | 5.08 (1.71) | 125 (13.8) | 80.2 (8.7) |
| Ruth et al., 2014 | No | No | Usual | 89% | Yes | Self-prepared | 12 | 42.5 (12.1) | 36.5 (4.7) | 4.71 (0.78) | 4.66 (0.9) | 120 (14.7) | 76.6 (9.35) |
| Schroeder et al., 2015 | No | No | Usual | 62% | No | Provided | 4 | 55.3 (8.7) | 31.3 (4.4) | 5.89 (0.80) | 5.52 (0.46) | 119.7 (11.7) | 73.6 (9.3) |
| von Frankenberg et al., 2015 | No | No | Usual | 30% | No | Provided | 4 | 36 (2.9) | 33.6 (1.3) | 4.45 (0.36) | 5 (0.1) | NR | NR |
| **LFLC** | | | | | | | | | | | | | |
| Kitabchi et al., 2013 | No | NR | Usual | 100% | Yes | Provided | 26 | 35.7 (2.05) | 39.2 (2.7) | NR | <6.1 | 128 (15.6) | 82.5 (12.1) |
| Waliłko et al., 2021 | NR | NR | Usual | 89% | Yes | Self-prepared | 4 | 41.5 (11) | 33.6 (4.2) | 5.4 (1.0) | 5.4 (0.5) | 119 (10) | 73 (8) |
| **LFMC (reference diet)** | | | | | | | | | | | | | |
| Andersson et al., 2016 | No | No | Usual | 100% | No | Self-prepared | 104 | 60.5 (1.3) | 32.6 (0.9) | 5.7 (0.5) | 5.15 (0.2) | 131.4 (9.6) | 82.8 (3.8) |
| Bajerska et al., 2018 | No | No | Usual | 100% | Yes | Self-prepared | 16 | 60.5 (4.7) | 33.7 (1.03) | 5.98 (1.09) | 5.44 (0.69) | 141.5 (14.5) | 87 (10.2) |
| Bazzano et al., 2014 | Yes | Yes | Usual | 88% | No | Self-prepared | 52 | 46.8 (10.1) | 35.4 (4.1) | 5.21 (1.1) | 5.21 (0.55) | 123 (13.4) | 78.5 (8.66) |
| Boers et al., 2014 | Yes | No | Usual | 73% | No | Provided | 2 | 53 (10) | 32 (5.57) | 5.9 (1.2) | 5.95 (0.76) | 133 (14.8) | 86.5 (11.0) |
| Brassard et al., 2017 | No | No | Usual | 53% | No | Provided | 4 | 39.2 (13.5) | 30.8 (6.05) | 4.94 (0.95) | 5.15  (0.5) | 114.35 (14.13) | 73.25 (11.14) |
| Chiu et al. 2014 | No | No | Usual | 70% | No | Self-prepared | 4 | 38 (12) | 33.9 (3.8) | 4.77 (0.65) | 5.23 (0.46) | 119 (13.4) | 71 (10.1) |
| Ebbeling et al., 2021 | No | No | Usual | 69% | No | Provided | 20 | 35.7 (range  24-52) | 32.2 (4.8) | 4.30 (0.89) | NR | 124 (10.7) | 76.6 (7.40) |
| Gadgil et al., 2013 | No | Yes | Usual | 45% | No | Provided | 6 | 53.6 (10.9) | 30.2 (6.1) | 5.20 (0.84) | 5.15 (0.85) | 131 (9.4) | 77 (8.2) |
| Guo et al., 2022 | NR | Yes | Moderate | 73% | Yes | Self-prepared | 10 | 39.2 (1.6) | 29.5 (1.86) | 5.26 (0.22) | 5.98 (0.3) | NR | NR |
| Jenkins et al., 2014 | Yes | No | Usual | 62% | Yes | Self-prepared | 30 | 56.5 (8.2) | 31.1 (1.25) | 6.76 (0.51) | 5.2 (0.22) | 125 (6.75) | 76 (3.55) |
| Juraschek et al., 2013 | No | 18% | Usual | 45% | No | Provided | 6 | 53.6 (10.9) | 30.2 (6.1) | 5.2 (0.84) | 5.15 (0.85) | 131 (9.4) | 77 (8.2) |
| Kitabchi et al., 2013 | No | NR | Usual | 100% | Yes | Provided | 26 | 35.7 (2.05) | 39.2 (2.7) | NR | <6.1 | 128 (15.6) | 82.5 (12.1) |
| Krishnan et al., 2018 | No | No | Usual | 100% | No | Provided | 8 | 46.9 (12.6) | 32.1 (3.89) | 5.38 (0.87) | 5.09 (0.52) | 122 (12.9) | 71.25 (8.86) |
| Mousavi et al., 2023 | No | No | Usual | 100% | Yes | Self-prepared | 13 | 40 (7.38) | 32.3 (3.61) | 5.25 (0.92) | 5.56 (0.63) | 122 (10.8) | 81.5 (7.9) |
| Poulsen et al., 2014 | Yes | NR | Usual | 71% | No | Self-prepared | 26 | 42 (13.) | 30.2 (4.87) | 4.63 (0.86) | 5.29 (0.47) | 122 (13.6) | 81.5 (9.94) |
| Sacks et al., 2014 | No | Yes | Usual | 52% | No | Provided | 5 | 53 (11) | 32 (6) | 6.01 (1.09) | 5.80 (0.95) | 132 (9.1) | 80 (7.5) |
| Schroeder et al., 2015 | No | No | Usual | 62% | No | Provided | 4 | 55.3 (8.7) | 31.3 (4.4) | 5.89 (0.80) | 5.52 (0.46) | 120 (11.7) | 73.6 (9.3) |
| Tricò et al., 2021 | NR | NR | Usual | 68% | Yes | Self-prepared | 4 | 44 (10.66) | 49 (7.18) | 4.8  (0.95) | 5.6  (0.6) | 137 (13.18) | 81  (6.54) |
| Veum et al., 2017 | No | NR | Usual | 0% | Yes | Self-prepared | 12 | 40.3 (5.2) | 33.7 (3.03) | 5.39 (1.14) | 5.05 (0.44) | 133 (14.54) | 85  (8.62) |
| Waliłko et al., 2021 | NR | NR | Usual | 89% | Yes | Self-prepared | 4 | 41.5 (11) | 33.6 (4.2) | 5.4 (1.0) | 5.4 (0.5) | 119 (10) | 73 (8) |
| **MFLC** | | | | | | | | | | | | | |
| Chiu et al. 2014 | No | No | Usual | 70% | No | Self-prepared | 4 | 38 (12) | 33.9 (3.8) | 4.77 (0.65) | 5.23 (0.46) | 119 (13.4) | 71 (10.1) |
| **MFMC** | | | | | | | | | | | | | |
| Brassard et al., 2017 | No | No | Usual | 53% | No | Provided | 4 | 39.2 (13.5) | 30.8 (6.05) | 4.94 (0.95) | 5.15  (0.5) | 114.35 (14.13) | 73.25 (11.14) |
| Fechner et al., 2020 (a) | No | No | Usual | 53% | No | Provided | 6 | 60.8 (6.3) | 29.3 (2.63) | 5.4  (0.84) | 5.7  (0.59) | 134  (13.1) | 84  (8.3) |
| Fechner et al., 2020 (b) | No | No | Usual | 53% | No | Provided | 6 | 60.8 (6.3) | 29.3 (2.63) | 5.4  (0.84) | 5.7  (0.59) | 134  (13.1) | 84  (8.3) |
| Krishnan et al., 2018 | No | No | Usual | 100% | No | Provided | 8 | 46.9 (12.6) | 32.1 (3.89) | 5.38 (0.87) | 5.09 (0.52) | 122.4 (12.95) | 71.25 (8.86) |
| Pinsawas et al., 2024 | Yes | No | Usual | 72% | Yes | Self-prepared | 12 | 40.9 (7.9) | 31.77 (0.87) | 5.02 (0.99) | 5.76 (0.35) | 130.43 (12.4) | 88.83 (9.3) |
| Poulsen et al., 2014 | Yes | NR | Usual | 71% | No | Self-prepared | 26 | 42 (13.09) | 30.2 (4.87) | 4.63 (0.86) | 5.29 (0.47) | 122.45 (13.63) | 81.45 (9.94) |
| Schroeder et al., 2015 | No | No | Usual | 62% | No | Provided | 4 | 55.3 (8.7) | 31.3 (4.4) | 5.89 (0.80) | 5.52 (0.46) | 119.7 (11.7) | 73.6  (9.3) |
| **hMUFA** | | | | | | | | | | | | | |
| Anderson-Vasquez et al., 2015 | No | NR | Usual | 100% | No | Self-prepared | 4 | 56  (5) | 29.8 (3.1) | 5.1  (0.42) | 4.97 (0.34) | NR | NR |
| Andersson et al., 2016 | No | No | Usual | 100% | No | Self-prepared | 104 | 60.5 (1.3) | 32.6 (0.9) | 5.7  (0.5) | 5.15  (0.2) | 131.4 (9.6) | 82.8  (3.8) |
| Bajerska et al., 2018 | No | No | Usual | 100% | Yes | Self-prepared | 16 | 60.5 (4.7) | 33.7 (1.03) | 5.98 (1.09) | 5.44 (0.69) | 141.5 (14.5) | 87  (10.2) |
| Brassard et al., 2017 | No | No | Usual | 53% | No | Provided | 4 | 39.2 (13.52) | 30.8 (6.05) | 4.94 (0.95) | 5.15  (0.5) | 114.35 (14.13) | 73.25 (11.14) |
| Chiu et al. 2014 | No | No | Usual | 70% | No | Self-prepared | 4 | 38  (12) | 33.9 (3.8) | 4.77 (0.65) | 5.23 (0.46) | 119  (13.4) | 71  (10.1) |
| Chiu et al. 2017 | No | No | Usual | 17% | No | Half-provided | 3 | 43  (13) | 29 (2.72) | 4.6  (0.91) | <7.0 | <150 | <90 |
| Ebbeling et al., 2021 | No | No | Usual | 69% | No | Provided | 20 | 35.7 (range 24-52) | 32.2 (4.8) | 4.3  (0.89) | NR | 123.7 (10.7) | 76.6  (7.4) |
| Gadgil et al., 2013 | No | Yes | Usual | 45% | No | Provided | 6 | 53.6 (10.9) | 30.2 (6.1) | 5.2  (0.84) | 5.15 (0.85) | 131.2 (9.4) | 77  (8.2) |
| Juraschek et al., 2013 | No | Yes | Usual | 45% | No | Provided | 6 | 53.6 (10.9) | 30.2 (6.1) | 5.2  (0.84) | 5.15 (0.85) | 131.2 (9.4) | 77  (8.2) |
| Marina et al., 2014 | No | No | Usual | 30% | No | Provided | 4 | 36  (2.9) | 33.6 (1.3) | 4.45 (0.36) | 5  (0.1) | NR | NR |
| von Frankenberg et al., 2015 | No | No | Usual | 30% | No | Provided | 4 | 36  (2.9) | 33.6 (1.3) | 4.45 (0.36) | 5  (0.1) | NR | NR |

Data for the continuous variables are shown as mean (SD) unless otherwise stated. **Abbreviations:** Interv., intervention; HFLC, high-fat—low-carbohydrate; HFMC, high-fat—moderate-carbohydrate; hMUFA, high-monounsaturated fatty acid; LFHC, low-fat—high-carbohydrate; LFLC, low-fat—low-carbohydrate; LFMC, low-fat—moderate-carbohydrate; MFLC, moderate-fat—low-carbohydrate; MFMC, moderate-fat—moderate-carbohydrate; BMI, body mass index; TC, total cholesterol; FG, fasting glucose; SBP, systolic blood pressure; DBP, diastolic blood pressure; NR, not reported

**Supplementary Table 7.** Network meta-analysis estimates for all outcomes. Mean differences (MD) with 95% confidence intervals (CI) are presented relative to the low-fat—moderate-carbohydrate (LFMC) reference diet. Certainty of evidence assessed using CiNeMA is shown in parentheses.

| **Diet** | **Adiposity** | | **Lipids** | | | | **Blood pressure** | | **Glycaemic** | |
| --- | --- | --- | --- | --- | --- | --- | --- | --- | --- | --- |
|  | **BMI (kg/m²)** | **WC (cm)** | **LDL-C (mmol/L)** | **HDL-C (mmol/L)** | **TC (mmol/L)** | **TG (mmol/L)** | **SBP (mmHg)** | **DBP (mmHg)** | **FG (mmol/L)** | **FI (mU/L)** |
|  | **MD [95% CI] (certainty of evidence)** | | | | | | | | | |
| **LFLC** | -0.50 [-4.13; 3.13] (Low) | n/a | -0.12 [-0.51; 0.27] (Low) | -0.15 [-0.40; 0.10] (Moderate) | -0.50 [-1.33; 0.33] (Low) | **-0.39 [-0.62; -0.16] (High)** | -2.00 [-6.16; 2.16] (Low) | -2.00 [-5.13; 1.13] (Low) | +0.17 [-0.10; 0.44] (Moderate) | n/a |
| **HFLC** | **-0.48 [-0.74; -0.22] (Low)** | -0.37 [-1.30; 0.55] (Low) | -0.04 [-0.15; 0.08] (Low) | **+0.03 [0.02; 0.05] (High)** | -0.01 [-0.20; 0.18] (Low) | **-0.20 [-0.27; -0.12] (Moderate)** | -1.39 [-2.88; 0.10] (Moderate) | **-1.01 [-1.74; -0.27] (Low)** | +0.02 [-0.07; 0.10] (Moderate) | +0.12 [-0.27; 0.51] (Low) |
| **HFMC** | +0.36 [-0.78; 1.51] (Low) | +0.15 [-6.37; 6.67] (Low) | +0.07 [-0.32; 0.45] (Moderate) | -0.04 [-0.09; 0.00] (Moderate) | +0.07 [-0.49; 0.63] (Low) | **-0.24 [-0.46; -0.03] (Moderate)** | n/a | n/a | -0.01 [-0.34; 0.33] (Low) | -0.47 [-5.17; 4.23] (Low) |
| **hMUFA** | +0.10 [-0.23; 0.43] (Moderate) | +0.05 [-1.14; 1.24] (Low) | -0.11 [-0.25; 0.03] (Low) | **+0.05 [0.02; 0.07] (High)** | -0.17 [-0.39; 0.05] (Moderate) | **-0.15 [-0.25; -0.04] (Moderate)** | -0.47 [-2.45; 1.51] (Low) | -0.44 [-1.58; 0.70] (Low) | -0.04 [-0.13; 0.06] (High) | -0.04 [-0.45; 0.36] (Moderate) |
| **MFLC** | +0.02 [-0.30; 0.35] (High) | -1.53 [-4.08; 1.02] (Low) | -0.16 [-0.48; 0.15] (Moderate) | +0.01 [-0.04; 0.07] (High) | -0.06 [-0.61; 0.48] (Low) | -0.13 [-0.50; 0.24] (Moderate) | n/a | n/a | n/a | 0.00 [-0.42; 0.43] (Low) |
| **MFMC** | +0.55 [-0.64; 1.74] (Low) | **+2.38 [1.04; 3.73] (High)** | **+0.24 [0.08; 0.39] (Moderate)** | **+0.06 [0.03; 0.09] (High)** | **+0.32 [0.10; 0.55] (Moderate)** | +0.08 [-0.05; 0.21] (High) | **+2.34 [0.17; 4.51] (Low)** | +1.10 [-0.32; 2.52] (Low) | -0.01 [-0.10; 0.08] (High) | **+0.22 [0.01; 0.44] (Moderate)** |
| **LFHC** | +0.66 [-0.46; 1.79] (Moderate) | +1.48 [-5.24; 8.21] (Low) | -0.08 [-0.35; 0.19] (Moderate) | **-0.09 [-0.16; -0.01] (High)** | -0.03 [-0.50; 0.44] (Low) | **+0.24 [0.04; 0.43] (Moderate)** | +3.64 [-1.22; 8.50] (Low) | +2.47 [-0.88; 5.82] (Low) | -0.06 [-0.24; 0.12] (Moderate) | -0.16 [-1.83; 1.51] (Low) |
| **Ketogenic** | +0.05 [-0.76; 0.85] (Low) | +1.80 [-0.45; 4.05] (Moderate) | **+0.43 [0.13; 0.74] (Moderate)** | **+0.12 [0.06; 0.18] (High)** | **+0.55 [0.13; 0.96] (Moderate)** | -0.08 [-0.29; 0.14] (Low) | -2.75 [-8.44; 2.94] (Low) | -0.15 [-4.05; 3.74] (Low) | +0.16 [-0.07; 0.38] (Moderate) | -1.11 [-3.49; 1.26] (Low) |
| **Literature-supported clinical relevance** | HFLC produced the only significant BMI reduction vs LFMC. Evidence shows ≥5% body weight loss is needed for clinically meaningful cardiometabolic benefit [7]. The ~0.5 kg/m² difference is unlikely to be clinically meaningful in isolation. | MFMC significantly increased WC vs LFMC, with modest clinical significance, as a >5 cm increase in WC has been associated with a higher subsequent CVD mortality risk [8]. | MFMC and Ketogenic significantly raised LDL-C vs LFMC, which corresponds to a modest clinical significance given that each 1.0 mmol/L LDL-C reduction is associated with ~22% lower major vascular event risk [9]. | Ketogenic, MFMC, hMUFA, HFLC significantly raised HDL-C vs LFMC. Epidemiological data indicate that a 0.026 mmol/L (1 mg/dL) increase in HDL is associated with a roughly 3.7-4.7% reduction in CVD mortality [10]. However, the observed increases in HDL-C in this analysis were small (0.03-0.12 mmol/L). | Ketogenic and MFMC significantly increased TC vs LFMC. TC is positively associated with CVD mortality and increases of this magnitude warrant monitoring in individuals at elevated CVD risk [11]. | LFLC, HFMC, HFLC, hMUFA significantly reduced TG vs LFMC, while LFHC significantly raised TG. Reductions in TG levels have been associated with approximately an 11-14% decrease in CVD risk per 1 mmol/L reduction [12], so these results are of modest clinical significance. | MFMC significantly raised SBP vs LFMC. Each 5 mmHg SBP reduction reduces major CV events by ~10% [9]. Hence, our the results show modest clinical significance. | HFLC is the only diet with a significant DBP reduction vs LFMC. A ~5 mmHg DBP reduction is required to produce ~20% lower major CVD events [9]. Hence, a ~1 mmHg reduction in DBP has limited clinical significance. | No diet produces a statistically significant difference in FG vs LFMC. | MFMC significantly raised FI vs LFMC, with only limited clinical significance. |

**Footnote:** All mean differences are estimated relative to LFMC (low-fat moderate-carbohydrate diet), which served as the reference node. A positive mean difference indicates a higher value relative to LFMC; a negative mean difference indicates a lower value. Statistically significant comparisons at the 5% significance level are shown in bold. Mean differences could not be estimated for systolic and diastolic blood pressure in the high-fat—moderate-carbohydrate (HFMC) versus LFMC and moderate-fat—low-carbohydrate (MFLC) versus LFMC comparisons, for fasting glucose in the moderate-fat—low-carbohydrate (MFLC) versus LFMC comparison, and for waist circumference and fasting insulin in the low-fat low—carbohydrate (LFLC) versus LFMC in the due to insufficient network connectivity.

**Abbreviations:** BMI, body mass index; WC, waist circumference; LDL-C, low-density lipoprotein cholesterol; HDL-C, high-density lipoprotein cholesterol; TC, total cholesterol; TG, triglycerides; SBP, systolic blood pressure; DBP, diastolic blood pressure; FG, fasting glucose; FI, fasting insulin; LFLC, low-fat—low-carbohydrate diet; HFLC, high-fat—low-carbohydrate diet; HFMC, high-fat—moderate-carbohydrate diet; hMUFA, high-monounsaturated fatty acid diet; MFLC, moderate-fat—low-carbohydrate diet; MFMC, moderate-fat—moderate-carbohydrate diet; LFHC, low-fat—high-carbohydrate diet; n/a, not available.

**Supplementary Table 8.** PRISMA NMA Checklist of Items to Include When Reporting A Systematic Review Involving a Network Meta-analysis

| **Section/Topic** | **Item #** | **Checklist Item** | **Reported on Page #** |
| --- | --- | --- | --- |
| **TITLE** |  |  |  |
| Title | 1 | Identify the report as a systematic review *incorporating a network meta-analysis (or related form of meta-analysis).* | ***1*** |
|  |  |  |  |
| **ABSTRACT** |  |  | ***2*** |
| Structured summary | 2 | Provide a structured summary including, as applicable:  **Background:** main objectives  **Methods:** data sources; study eligibility criteria, participants, and interventions; study appraisal; and *synthesis methods, such as network meta-analysis.*  **Results:** number of studies and participants identified; summary estimates with corresponding confidence/credible intervals; *treatment rankings may also be discussed. Authors may choose to summarize pairwise comparisons against a chosen treatment included in their analyses for brevity.*  **Discussion/Conclusions:** limitations; conclusions and implications of findings.  **Other:** primary source of funding; systematic review registration number with registry name. |  |
|  |  |  |  |
| **INTRODUCTION** |  |  |  |
| Rationale | 3 | Describe the rationale for the review in the context of what is already known*, including mention of why a network meta-analysis has been conducted.* | ***3*** |
| Objectives | 4 | Provide an explicit statement of questions being addressed, with reference to participants, interventions, comparisons, outcomes, and study design (PICOS). | 3-4 |
|  |  |  |  |
| **METHODS** |  |  |  |
| Protocol and registration | 5 | Indicate whether a review protocol exists and if and where it can be accessed (e.g., Web address); and, if available, provide registration information, including registration number. | 4 |
| Eligibility criteria | 6 | Specify study characteristics (e.g., PICOS, length of follow-up) and report characteristics (e.g., years considered, language, publication status) used as criteria for eligibility, giving rationale. *Clearly describe eligible treatments included in the treatment network, and note whether any have been clustered or merged into the same node (with justification).* | ***4*** |
| Information sources | 7 | Describe all information sources (e.g., databases with dates of coverage, contact with study authors to identify additional studies) in the search and date last searched. | 4 |
| Search | 8 | Present full electronic search strategy for at least one database, including any limits used, such that it could be repeated. | 4 |
| Study selection | 9 | State the process for selecting studies (i.e., screening, eligibility, included in systematic review, and, if applicable, included in the meta-analysis). | 4 |
| Data collection process | 10 | Describe method of data extraction from reports (e.g., piloted forms, independently, in duplicate) and any processes for obtaining and confirming data from investigators. | 5 |
| Data items | 11 | List and define all variables for which data were sought (e.g., PICOS, funding sources) and any assumptions and simplifications made. | 5 |
| **Geometry of the network** | **S1** | Describe methods used to explore the geometry of the treatment network under study and potential biases related to it. This should include how the evidence base has been graphically summarized for presentation, and what characteristics were compiled and used to describe the evidence base to readers. | ***5*** |
| Risk of bias within individual studies | 12 | Describe methods used for assessing risk of bias of individual studies (including specification of whether this was done at the study or outcome level), and how this information is to be used in any data synthesis. | 5 |
| Summary measures | 13 | State the principal summary measures (e.g., risk ratio, difference in means). *Also describe the use of additional summary measures assessed, such as treatment rankings and surface under the cumulative ranking curve (SUCRA) values, as well as modified approaches used to present summary findings from meta-analyses.* | 5 |
| Planned methods of analysis | 14 | Describe the methods of handling data and combining results of studies for each network meta-analysis. This should include, but not be limited to:   1. *Handling of multi-arm trials;* 2. *Selection of variance structure;* 3. *Selection of prior distributions in Bayesian analyses; and* 4. *Assessment of model fit.* | 5 |
| **Assessment of Inconsistency** | **S2** | Describe the statistical methods used to evaluate the agreement of direct and indirect evidence in the treatment network(s) studied. Describe efforts taken to address its presence when found. | 5 |
| Risk of bias across studies | 15 | Specify any assessment of risk of bias that may affect the cumulative evidence (e.g., publication bias, selective reporting within studies). | **5** |
| Additional analyses | 16 | Describe methods of additional analyses if done, indicating which were pre-specified. This may include, but not be limited to, the following:   1. Sensitivity or subgroup analyses; 2. Meta-regression analyses; 3. *Alternative formulations of the treatment network; and* 4. *Use of alternative prior distributions for Bayesian analyses (if applicable).* | ***5*** |
| **RESULTS†** |  |  |  |
| Study selection | 17 | Give numbers of studies screened, assessed for eligibility, and included in the review, with reasons for exclusions at each stage, ideally with a flow diagram. | 6 |
| **Presentation of network structure** | **S3** | Provide a network graph of the included studies to enable visualization of the geometry of the treatment network. | ***6*** |
| **Summary of network geometry** | **S4** | Provide a brief overview of characteristics of the treatment network. This may include commentary on the abundance of trials and randomized patients for the different interventions and pairwise comparisons in the network, gaps of evidence in the treatment network, and potential biases reflected by the network structure. | ***6*** |
| Study characteristics | 18 | For each study, present characteristics for which data were extracted (e.g., study size, PICOS, follow-up period) and provide the citations. | 6 |
| Risk of bias within studies | 19 | Present data on risk of bias of each study and, if available, any outcome level assessment. | 7-8 |
| Results of individual studies | 20 | For all outcomes considered (benefits or harms), present, for each study: 1) simple summary data for each intervention group, and 2) effect estimates and confidence intervals. *Modified approaches may be needed to deal with information from larger networks.* | *8-10* |
| Synthesis of results | 21 | Present results of each meta-analysis done, including confidence/credible intervals. *In larger networks, authors may focus on comparisons versus a particular comparator (e.g. placebo or standard care), with full findings presented in an appendix. League tables and forest plots may be considered to summarize pairwise comparisons.* If additional summary measures were explored (such as treatment rankings), these should also be presented. | *8-10* |
| **Exploration for inconsistency** | **S5** | Describe results from investigations of inconsistency. This may include such information as measures of model fit to compare consistency and inconsistency models, *P* values from statistical tests, or summary of inconsistency estimates from different parts of the treatment network. | ***7*** |
| Risk of bias across studies | 22 | Present results of any assessment of risk of bias across studies for the evidence base being studied. | 7 |
| Results of additional analyses | 23 | Give results of additional analyses, if done (e.g., sensitivity or subgroup analyses, meta-regression analyses*, alternative network geometries studied, alternative choice of prior distributions for Bayesian analyses,* and so forth). | ***10*** |
| **DISCUSSION** |  |  |  |
| Summary of evidence | 24 | Summarize the main findings, including the strength of evidence for each main outcome; consider their relevance to key groups (e.g., healthcare providers, users, and policy-makers). | 11-12 |
| Limitations | 25 | Discuss limitations at study and outcome level (e.g., risk of bias), and at review level (e.g., incomplete retrieval of identified research, reporting bias). *Comment on the validity of the assumptions, such as transitivity and consistency. Comment on any concerns regarding network geometry (e.g., avoidance of certain comparisons).* | 13 |
| Conclusions | 26 | Provide a general interpretation of the results in the context of other evidence, and implications for future research. | 14 |
| **FUNDING** |  |  | 14 |
| Funding | 27 | Describe sources of funding for the systematic review and other support (e.g., supply of data); role of funders for the systematic review. This should also include information regarding whether funding has been received from manufacturers of treatments in the network and/or whether some of the authors are content experts with professional conflicts of interest that could affect use of treatments in the network. |  |

PICOS = population, intervention, comparators, outcomes, study design.

* Text in italics indicateS wording specific to reporting of network meta-analyses that has been added to guidance from the PRISMA statement.

† Authors may wish to plan for use of appendices to present all relevant information in full detail for items in this section.

**REFERENCES**

1. Hutton B, Salanti G, Caldwell DM, Chaimani A, Schmid CH, Cameron C, et al. The PRISMA extension statement for reporting of systematic reviews incorporating network meta-analyses of health care interventions: checklist and explanations. Ann Intern Med 2015;162:777-84. <https://doi.org/10.7326/M14-2385>
2. Schiavo JH. PROSPERO: An International Register of Systematic Review Protocols. Med Ref Serv Q 2019;38:171-180. <https://doi.org/10.1080/02763869.2019.1588072>
3. R Core Team (2025). R: A language and environment for statistical computing. R Foundation for Statistical Computing, Vienna, Austria. URL <https://www.R-project.org/>.
4. Balduzzi S, Rücker G, Nikolakopoulou A, Papakonstantinou T, Salanti G, Efthimiou O, et al. netmeta: An R Package for Network Meta-Analysis Using Frequentist Methods. J Stat Softw 2023;106:1-40. <https://doi.org/10.18637/jss.v106.i02>
5. Higgins JPT, Thomas J, Chandler J, Cumpston M, Li T, Page MJ, et al., editors (2024) Cochrane Handbook for Systematic Reviews of Interventions version 6.5 (updated August 2024). Cochrane. Available from [www.cochrane.org/handbook](http://www.cochrane.org/handbook)
6. Ma Y, Zheng Z, Zhuang L, et al. Dietary Macronutrient Intake and Cardiovascular Disease Risk and Mortality: A Systematic Review and Dose-Response Meta-Analysis of Prospective Cohort Studies. Nutrients. 2024;16(1):152. https://doi.org/10.3390/nu16010152
7. Brown JD, Buscemi J, Milsom V, Malcolm R, O'Neil PM. Effects on cardiovascular risk factors of weight losses limited to 5–10%. Transl Behav Med 2016;6:339-46. <https://doi.org/10.1007/s13142-015-0353-9>
8. Mulligan AA, Lentjes MAH, Luben RN, Wareham NJ, Khaw KT. Changes in waist circumference and risk of all-cause and CVD mortality: results from the European Prospective Investigation into Cancer in Norfolk (EPIC-Norfolk) cohort study. BMC Cardiovasc Disord. 2019;19(1):238. <https://doi.org/10.1186/s12872-019-1223-z>
9. Cholesterol Treatment Trialists C, Baigent C, Blackwell L, Emberson J, Holland LE, Reith C, et al. Efficacy and safety of more intensive lowering of LDL cholesterol: a meta-analysis of data from 170,000 participants in 26 randomised trials. Lancet 2010;376:1670-81. <https://doi.org/10.1016/S0140-6736(10)61350-5>
10. Hamer M, O’Donovan G, Stamatakis E. High-density lipoprotein cholesterol and mortality. Arterioscler Thromb Vasc Biol 2018;38:669-72. <https://doi.org/10.1161/ATVBAHA.117.310587>
11. Jung E, Kong SY, Ro YS, Ryu HH, Shin SD. Serum Cholesterol Levels and Risk of Cardiovascular Death: A Systematic Review and a Dose-Response Meta-Analysis of Prospective Cohort Studies. Int J Environ Res Public Health. 2022;19(14):8272. <https://doi.org/10.3390/ijerph19148272>
12. Moura JPd. Does lowering triglycerides reduce cardiovascular risk? Rev Port Cardiol 2019;38:543-5. <https://doi.org/10.1016/j.repce.2019.11.005>
